# Supplementary material for: An approximate-copula distribution for statistical modeling
Source: PLoS Comput Biol. 2026 Mar 13;22(3):e1013922. doi: 10.1371/journal.pcbi.1013922 (PMC12998956; doi:10.1371/journal.pcbi.1013922)
Supplement: S1 File — (PDF) [file pcbi.1013922.s001.pdf]

Supplement to: An Approximate-Copula Distribution for Statistical Modeling

*Sarah S. Ji[1†], Benjamin B. Chu[2], Hua Zhou[1,4], Kenneth Lange[3,4,5]

[1]Department of Biostatistics, University of California, Los Angeles, CA

[2]Department of Biomedical Data Science, Stanford University, Stanford, CA

[3]Department of Human Genetics, University of California, Los Angeles, CA

[4]Department of Computational Medicine, University of California, Los Angeles, CA

[5]Department of Statistics, University of California, Los Angeles, CA

January 23, 2026

Contents

S1 Supplemental Materials **2**

S1.1 Tonda's Approximation Details 2

S1.2 Generate Random Deviates 3

S1.2.1 Marginal Distribution 4

S1.2.2 Conditional Distribution 5

S1.2.3 Continuous Outcomes 6

S1.2.4 Discrete Outcomes 12

S1.3 Parameter Estimation: 16

S1.3.1 Fisher Scoring to Estimate Beta 16

S1.3.2 MM Algorithm for the VC Model Parameters 19

S1.3.3 Quasi-Newton Algorithm 20

*Address all correspondence to smji@g.ucla.edu.

†K.L is supported by GM141798. H.Z. is supported by NIH R01 DK142026, NSF DMS-2054253, NSF IIS-2205441, and NIH R35 GM141798. B.C. is supported by R01MH113078, R01MH123157, R56HG010812. S.J is supported by HG002536. The funders had no role in study design, data collection and analysis, decision to publish, or preparation of the manuscript.

S1.3.4 Nuisance parameter estimation for Negative Binomial base . . 21
S1.3.5 Compound Symmetric $\mathbf{\Gamma}_i$ 24
S1.3.6 Gradients and Hessians of residual function 25
S1.4 Special case: Gaussian approximate-copulas 26
S1.4.1 Moments 27
S1.4.2 Parameter Estimation 29
S1.5 Additional Simulations for longitudinal model 33
S1.5.1 Additional Run Times 38
S1.6 Additional simulations for multivariate model 43
S1.6.1 Is the SNP screening procedure valid? 43
S1.6.2 QQ plots 44
S1.7 Additional results from the NHANES logntudinal analysis 44
S1.8 Quality control on UK-Biobank genotypes 46
S1.9 Additional simulations for negative binomial longitudinal models . . . 47
S1.10Cauchy combination test as an alternative to multivariate GWAS . . 48

S1 Supplemental Materials

S1.1 Tonda's Approximation Details

Let $\boldsymbol{x}$ be a random vector with exponential density $f(\boldsymbol{x} \mid \boldsymbol{\nu}) = e^{\boldsymbol{T}(\boldsymbol{x})^\top \boldsymbol{\nu} - A(\boldsymbol{\nu})}$. Note that $\boldsymbol{T}(\boldsymbol{x})$ has mean $\boldsymbol{\mu}(\boldsymbol{\nu}) = \nabla A(\boldsymbol{\nu})$ and covariance matrix $d^2 A(\boldsymbol{\nu})$. Let us shift $\boldsymbol{\nu}$ by adding a random Gaussian $\boldsymbol{z}$ with mean $\mathbf{0}$ and covariance $\mathbf{\Sigma}$. The new density $E[e^{\boldsymbol{T}(\boldsymbol{x})^\top(\boldsymbol{\nu}+\boldsymbol{z}) - A(\boldsymbol{\nu}+\boldsymbol{z})}]$ can be approximated by expanding the integrand to second

order around $\boldsymbol{z} = \mathbf{0}$ and integrating. This yields

$$\begin{aligned}
\mathrm{E}[e^{\boldsymbol{T}(\boldsymbol{x})^\top(\boldsymbol{\nu}+\boldsymbol{z})-A(\boldsymbol{\nu}+\boldsymbol{z})}] &\approx \mathrm{E}\Big(e^{\boldsymbol{T}(\boldsymbol{x})^\top(\boldsymbol{\nu})-A(\boldsymbol{\nu})}\{1+[\boldsymbol{T}(\boldsymbol{x})-\nabla A(\boldsymbol{\nu})]^\top\boldsymbol{z} \\
&\quad +\frac{1}{2}\boldsymbol{z}^\top[\boldsymbol{T}(\boldsymbol{x})-\nabla A(\boldsymbol{\nu})][\boldsymbol{T}(\boldsymbol{x})-\nabla A(\boldsymbol{\nu})]^\top\boldsymbol{z} \\
&\quad -\frac{1}{2}\boldsymbol{z}^\top d^2A(\boldsymbol{\nu})\boldsymbol{z}\}\Big) \\
&= e^{\boldsymbol{T}(\boldsymbol{x})^\top(\boldsymbol{\nu})-A(\boldsymbol{\nu})}\Big(1+\frac{1}{2}\operatorname{tr}\{[\boldsymbol{T}(\boldsymbol{x})-\nabla A(\boldsymbol{\nu})][\boldsymbol{T}(\boldsymbol{x})-\nabla A(\boldsymbol{\nu})]^\top\boldsymbol{\Sigma}\} \\
&\quad -\frac{1}{2}\operatorname{tr}[d^2A(\boldsymbol{\nu})\boldsymbol{\Sigma}]\Big) \\
&= e^{\boldsymbol{T}(\boldsymbol{x})^\top(\boldsymbol{\nu})-A(\boldsymbol{\nu})}\Big(1+\frac{1}{2}\operatorname{tr}\{[\boldsymbol{T}(\boldsymbol{x})-\boldsymbol{\mu}(\boldsymbol{\nu})][\boldsymbol{T}(\boldsymbol{x})-\boldsymbol{\mu}(\boldsymbol{\nu})]^\top\boldsymbol{\Sigma}\} \\
&\quad -\frac{1}{2}\operatorname{tr}[d^2A(\boldsymbol{\nu})\boldsymbol{\Sigma}]\Big) \\
&= e^{\boldsymbol{T}(\boldsymbol{x})^\top(\boldsymbol{\nu})-A(\boldsymbol{\nu})}\Big\{1+\frac{1}{2}\boldsymbol{W}^\top\sqrt{d^2A(\boldsymbol{\nu})}\boldsymbol{\Sigma}\sqrt{d^2A(\boldsymbol{\nu})}\boldsymbol{W} \\
&\quad -\frac{1}{2}\operatorname{tr}[\sqrt{d^2A(\boldsymbol{\nu})}\boldsymbol{\Sigma}\sqrt{d^2A(\boldsymbol{\nu})}]\Big\},
\end{aligned}$$

where $\boldsymbol{W}$ is the standardized version $[\boldsymbol{T}(\boldsymbol{x})-\boldsymbol{\mu}(\boldsymbol{\nu})]d^2A(\boldsymbol{\nu})^{-1/2}$ of the base sufficient statistic $\boldsymbol{T}(\boldsymbol{x})$. The condition $1 - -\frac{1}{2}\operatorname{tr}[\sqrt{d^2A(\boldsymbol{\nu})}\boldsymbol{\Sigma}\sqrt{d^2A(\boldsymbol{\nu})}] > 0$ is sufficient but not necessary for the approximate density to be nonnegative. When this condition holds, the approximate density has mass 1. In our approximate-copula density, we drop the offending term $-\frac{1}{2}\operatorname{tr}[\sqrt{d^2A(\boldsymbol{\nu})}\boldsymbol{\Sigma}\sqrt{d^2A(\boldsymbol{\nu})}]$, replace $\sqrt{d^2A(\boldsymbol{\nu})}\boldsymbol{\Sigma}\sqrt{d^2A(\boldsymbol{\nu})}$ by $\boldsymbol{\Gamma}$, assume $T(\boldsymbol{x}) = \boldsymbol{x}$, and normalize.

S1.2 Generate Random Deviates

We can construct the d dimensional multivariate vector, $\mathbf{y}$ from the multivariate density $g_{\mathbf{y}}(\mathbf{y})$ element wise using conditional densities. We recognize the joint density can be represented as a product of conditional densities:

$$g_{\mathbf{y}}(\mathbf{y}) = g_{y_1}(y_1) \times g_{y_2|y_1}(y_2|y_1) \times ... \times g_{y_d|y_1,...,y_{d-1}}(y_d|y_1,...,y_{d-1})$$

Thus we can first sample y_1 from its marginal density $g_{y_1}(y_1)$, and then sample y_2 from the conditional density $g_{y_2|y_1}(y_2|y_1)$. The resulting set is a sample from the joint density of $g_{y_1,y_2}(y_1, y_2)$. Continuing this process for all n values of the multivariate vector, $\mathbf{y}$, we can sample from it's joint density $g_{\mathbf{y}}(\mathbf{y})$. First we derive the form of the marginal densities $g_{y_1}(y_1)$, and then show the derivation of the conditional density $g_{y_d|y_1,...,y_{d-1}}(y_d|y_1,...,y_{d-1})$.

S1.2.1 Marginal Distribution

For every univariate base distribution, the required probability density functions (PDFs) $g_y(y)$ are of the same form, where c_0, c_1 and c_2 are constants that depend on the parameters of the specified base distribution $f_y(y)$.

$$g_y(y) = cf_y(y)[a_0 + a_1(y-\mu) + a_2(y-\mu)^2] \quad (1)$$
$$= cf_y(y) \times \Big[c_0 + c_1 y + c_2 y^2\Big], \quad (2)$$

We can re-arrange the PDF to derive the constants c_0, c_1, c_2 in the marginal PDF $g_y(y)$ as follows:

$$\begin{aligned}
g_y(y) &= \left(1+\frac{1}{2}\mathrm{tr}\mathbf{\Gamma}\right)^{-1} f_y(y)\Big[1+\frac{\gamma_{11}}{2}\Big(\frac{y-\mu}{\sigma}\Big)^2+\frac{1}{2}\sum_{j=2}^{d}\gamma_{jj}\Big] \\
&= \left(1+\frac{1}{2}\mathrm{tr}\mathbf{\Gamma}\right)^{-1} f_y(y)\Big[1+\frac{\gamma_{11}}{2}\left(\frac{y^2-2y\mu+\mu^2}{\sigma^2}\right)+\frac{1}{2}\sum_{j=2}^{d}\gamma_{jj}\Big] \\
&= \left(1+\frac{1}{2}\mathrm{tr}\mathbf{\Gamma}\right)^{-1} f_y(y)\Big[1+\frac{\gamma_{11}}{2}\left(\frac{y^2}{\sigma^2}\right)+\frac{\gamma_{11}}{2}\left(\frac{-2y\mu}{\sigma^2}\right)+\frac{\gamma_{11}}{2}\left(\frac{\mu^2}{\sigma^2}\right)+\frac{1}{2}\sum_{j=2}^{d}\gamma_{jj}\Big] \\
&= \left(1+\frac{1}{2}\mathrm{tr}\mathbf{\Gamma}\right)^{-1} f_y(y)\left[\left(1+\frac{\gamma_{11}}{2}\Big(\frac{\mu^2}{\sigma^2}\Big)+\frac{1}{2}\sum_{j=2}^{d}\gamma_{jj}\right)+\left(\frac{\gamma_{11}}{2}\Big(\frac{-2\mu}{\sigma^2}\Big)\right)y+\left(\frac{\gamma_{11}}{2}\Big(\frac{1}{\sigma^2}\Big)\right)y^2\right] \\
&= c \times f_y(y)\Big[\Big(c_0\Big)+\Big(c_1\Big)y+\Big(c_2\Big)y^2\Big],
\end{aligned}$$

- $c = \left[1+\frac{1}{2}\mathrm{tr}(\mathbf{\Gamma})\right]^{-1}$, for all base distributions $f_y(y)$.
- $c_0 = \left(1+\frac{\gamma_{11}}{2}\left(\frac{\mu^2}{\sigma^2}\right)+\frac{1}{2}\sum_{j=2}^{d}\gamma_{jj}\right)$,
- $c_1 = \left(\frac{\gamma_{11}}{2}\left(\frac{-2\mu}{\sigma^2}\right)\right)$,
- $c_2 = \left(\frac{\gamma_{11}}{2}\left(\frac{1}{\sigma^2}\right)\right)$

Thus, the required marginal Cumulative Distribution Function (CDF) $G_y(x)$ takes the following form. We will derive the CDF by finding the appropriate scaled cumulative distributions of the three terms.

$$\begin{aligned}
G_y(x) &= \int_0^{\infty} g_y(y)\,dy \\
&= c\int_{-\infty}^{x} f(y)[c_0 + c_1 y + c_2 y^2]\,dy \\
&= c \times c_0 \int_{-\infty}^{x} f_y(y)\,dy \\
&+ c \times c_1 \int_{-\infty}^{x} y f_y(y)\,dy \\
&+ c \times c_2 \int_{-\infty}^{x} y^2 f_y(y)\,dy \\
&= \textbf{term1} + \textbf{term2} + \textbf{term3}
\end{aligned}$$

The first term is a scalar multiple of the base distribution CDF, $F_y(y)$, and d_1, d_2 are normalizing constants for random variables v_1, v_2 from named distributions $f_{v_1}(v_1), f_{v_2}(v_2)$ with CDFs $F_{v_1}(x)$ and $F_{v_2}(x)$ in terms 2 and 3, respectively.

- **term1** $= c \times c_0 \int_{-\infty}^{x} f_y(y)\,dy = c \times (c_0) \times F_y(x)$,
- **term2** $= c \times c_1 \int_{-\infty}^{x} y * f_y(y)\,dy = c \times c_1 \times d_1 \times \int_{-\infty}^{x} f_{v_1}(y)\,dy = c \times (c_1) \times d_1 \times F_{v_1}(x)$
- **term3** $= c \times c_2 \int_{-\infty}^{x} y^2 f_y(y)\,dy = c \times c_2 \times d_2 \times \int_{-\infty}^{x} f_{v_2}(y)\,dy = c \times (c_2) \times d_2 \times F_{v_2}(x)$

For every base distribution, to satisfy properties of a proper distribution function we require

$$c \times [c_0 + c_1 \times d_1 + c_2 \times d_2] = 1.$$

S1.2.2 Conditional Distribution

Let $\mathbf{y_{[i-1]}}$ indicate elements $y_1, ..., y_{i-1}, \forall i \in [1, d]$. Then the conditional density of y_i given the previous components $\mathbf{y_{[i-1]}}$ is:

$$
\begin{aligned}
& g_{y_i|\mathbf{y_{[i-1]}}}(y_i|\mathbf{y_{[i-1]}}) \\
= \;& d_{[i-1]}^{-1} f_i(y_i)\Big[d_{[i-1]} + r_i \sum_{j=1}^{i-1} r_j\gamma_{ij} + \frac{\gamma_{ii}}{2}(r_i^2-1)\Big] \\
= \;& d_{[i-1]}^{-1} f_i(y_i)\Big[d_{[i-1]} + \Big(\frac{y_i-\mu_i}{\sigma_i}\Big) \sum_{j=1}^{i-1} r_j\gamma_{ij} + \frac{\gamma_{ii}}{2}(r_i^2-1)\Big] \\
= \;& d_{[i-1]}^{-1} f_i(y_i)\Big[\Big(d_{[i-1]}^{-1} - \frac{\gamma_{ii}}{2}\Big) + \Big(\frac{\sum_{j=1}^{i-1} r_j\gamma_{ij}}{\sigma_i}\Big)y_i - \mu_i\Big(\frac{\sum_{j=1}^{i-1} r_j\gamma_{ij}}{\sigma_i}\Big) + \frac{\gamma_{ii}}{2}\frac{(y_i-\mu_i)^2}{\sigma_i^2}\Big] \\
= \;& d_{[i-1]}^{-1} f_i(y_i)\Big[\Big(d_{[i-1]}^{-1} - \frac{\gamma_{ii}}{2} - \mu_i\Big(\frac{\sum_{j=1}^{i-1} r_j\gamma_{ij}}{\sigma_i}\Big)\Big) + \Big(\frac{\sum_{j=1}^{i-1} r_j\gamma_{ij}}{\sigma_i}\Big)y_i + \frac{\gamma_{ii}}{2}\frac{(y_i-\mu_i)^2}{\sigma_i^2}\Big] \\
= \;& d_{[i-1]}^{-1} f_i(y_i)\Big[\Big(d_{[i-1]}^{-1} - \frac{\gamma_{ii}}{2} - \mu_i\Big(\frac{\sum_{j=1}^{i-1} r_j\gamma_{ij}}{\sigma_i}\Big) + \frac{\gamma_{ii}}{2}\frac{\mu_i^2}{\sigma_i^2}\Big) \\
+ \;& \Big(\frac{\sum_{j=1}^{i-1} r_j\gamma_{ij}}{\sigma_i} + \frac{\gamma_{ii}}{2}\Big(\frac{-2\mu_i}{\sigma_i^2}\Big)\Big)y_i \\
+ \;& \Big(\frac{\gamma_{ii}}{2}\Big(\frac{1}{\sigma_i^2}\Big)\Big)y_i^2\Big] \\
= \;& c f_i(y_i)\Big[c_0 + c_1 y_i + c_2 y_i^2\Big]
\end{aligned}
$$

where $d_{[i-1]} = 1 + \frac{1}{2} r_{[i-1]}^\top \mathbf{\Gamma}_{[i-1]} r_{[i-1]} + \frac{1}{2}\sum_{j=i}^{d} \gamma_{jj}$.

- $c = d_{[i-1]}^{-1}$
- $c_0 = \Big(d_{[i-1]}^{-1} - \frac{\gamma_{ii}}{2} - \mu_i\Big(\frac{\sum_{j=1}^{i-1} r_j\gamma_{ij}}{\sigma_i}\Big) + \frac{\gamma_{ii}}{2}\frac{\mu_i^2}{\sigma_i^2}\Big)$
- $c_1 = \Big(\frac{\sum_{j=1}^{i-1} r_j\gamma_{ij}}{\sigma_i} + \frac{\gamma_{ii}}{2}\Big(\frac{-2\mu_i}{\sigma_i^2}\Big)\Big)$
- $c_2 = \Big(\frac{\gamma_{ii}}{2}\Big(\frac{1}{\sigma_i^2}\Big)\Big)$

We can construct each conditional density given the previously sampled elements as a combination of three constants, just as in the marginal density.

S1.2.3 Continuous Outcomes

When marginal densities $f(y)$ are continuous, each stage of sampling is probably best performed by inverse transform sampling.

Gamma distribution This note considers the special case of Gamma base in the copula framework outlined in Ken's notes, where $f_1(y) \sim \Gamma(\alpha, \theta)$. We will simulate y directly from its marginal density, $g_y(y)$, which can also be represented as a mixture distribution.

$$y \sim \Gamma(\alpha, \theta); f_y(y) = \frac{1}{\Gamma(\alpha)\theta^{\alpha}} y^{\alpha-1} e^{\frac{-y}{\theta}}$$

- $\mu = E[y] = \alpha\theta$, and $\sigma^2 = Var(y) = \alpha\theta^2$.

$$\begin{aligned}
g_y(y) &= \Big[1 + \frac{1}{2}\operatorname{tr}(\mathbf{\Gamma})\Big]^{-1} \Big[\frac{1}{\Gamma(\alpha)\theta^{\alpha}} y^{\alpha-1} e^{\frac{-y}{\theta}}\Big] \Big(1 + \frac{\gamma_{11}}{2}\left[\frac{(y-\mu)^2}{\sigma^2}\right] + \frac{1}{2}\sum_{j=2}^{d}\gamma_{jj}\Big) \\
&= \Big[1 + \frac{1}{2}\operatorname{tr}(\mathbf{\Gamma})\Big]^{-1} \Big[\frac{1}{\Gamma(\alpha)\theta^{\alpha}} y^{\alpha-1} e^{\frac{-y}{\theta}}\Big] \left(\Big(1 + \frac{1}{2}\sum_{j=2}^{d}\gamma_{jj}\Big) + \frac{\gamma_{11}}{2}\left[\frac{y^2 - 2y\mu + \mu^2}{\sigma^2}\right]\right) \\
&= c \times f_y(y)\Big[\Big(c_0\Big) + \Big(c_1\Big)y + \Big(c_2\Big)y_2^2\Big]
\end{aligned}$$

- Here $c_0 = \left(1 + \frac{\gamma_{11}}{2}\left(\frac{\mu^2}{\sigma^2}\right) + \frac{1}{2}\sum_{j=2}^{d}\gamma_{jj}\right) = \left(1 + \frac{\gamma_{11}}{2}\Big(\alpha\Big) + \frac{1}{2}\sum_{j=2}^{d}\gamma_{jj}\right)$
- $c_1 = \left(\frac{\gamma_{11}}{2}\left(\frac{-2\mu}{\sigma^2}\right)\right) = \left(\frac{\gamma_{11}}{2}\left(\frac{-2}{\theta}\right)\right)$
- $c_2 = \left(\frac{\gamma_{11}}{2}\left(\frac{1}{\sigma^2}\right)\right) = \left(\frac{\gamma_{11}}{2}\left(\frac{1}{\alpha\theta^2}\right)\right)$

We will use the given information here: $y \sim \Gamma(\alpha, \theta)$, where $F_Y(x) = P(y \leq x)$ to derive the CDF $G_Y(x)$ term by term.

$$\begin{aligned}
\textbf{term1} &= c \times (c_0)\int_0^x f_y(y)dy \\
&= c \times (c_0) \times F_Y(Y = x) \\
&= c \times \left(1 + \frac{\gamma_{11}}{2}\Big(\alpha\Big) + \frac{1}{2}\sum_{j=2}^{m}\gamma_{jj}\right) \times F_Y(Y = x)
\end{aligned}$$

Define a new random variable $v_1 \sim \Gamma(\alpha+1, \theta)$, where $F_{v_1}(x) = P(v_1 \leq x)$.

$$\begin{aligned}
\textbf{term2} &= c \times (c_1) \times \int_0^x y f_y(y) dy \\
&= c \times \left(\frac{\gamma_{11}}{2}\Big(\frac{-2}{\theta}\Big)\right) \times \int_0^x y f_y(y) dy \\
&= c \times \left(\frac{\gamma_{11}}{2}\Big(\frac{-2}{\theta}\Big)\right) \times \theta \int_0^x \frac{y}{\theta} f_y(y) dy \\
&= c \times \left(\frac{\gamma_{11}}{2}\Big(\frac{-2}{\theta}\Big)\right) \times \theta \times \int_0^x \Big[\frac{1}{\Gamma(\alpha)\theta^{\alpha+1}} y^{(\alpha+1)-1} e^{\frac{-y}{\theta}}\Big] dy \\
&= c \times \left(\frac{\gamma_{11}}{2}\Big(\frac{-2}{\theta}\Big)\right) \times \theta \times \frac{\Gamma(\alpha+1)}{\Gamma(\alpha)} \times \int_0^x \Big[\frac{1}{\Gamma(\alpha+1)\theta^{\alpha+1}} y^{(\alpha+1)-1} e^{\frac{-y}{\theta}}\Big] dy \\
&= c \times \left(\frac{\gamma_{11}}{2}\Big(\frac{-2}{\theta}\Big)\right) \times \theta \times \frac{\Gamma(\alpha+1)}{\Gamma(\alpha)} \times F_{v_1}(x)
\end{aligned}$$

Define another random variable $v_2 \sim \Gamma(\alpha+2, \theta)$; with CDF $F_{v_2}(x) = P(v_2 \leq x)$.

$$\begin{aligned}
\textbf{term 3} &= c \times (c_2) \times \int_0^x y^2 f_y(y)\, dy \\
&= c \times \left(\frac{\gamma_{11}}{2}\Big(\frac{1}{\alpha\theta^2}\Big)\right) \times \int_0^x y^2 f_y(y)\, dy \\
&= c \times \left(\frac{\gamma_{11}}{2}\Big(\frac{1}{\alpha\theta^2}\Big)\right) \times \theta^2 \int_0^x \frac{y^2}{\theta^2} f_y(y)\, dy \\
&= c \times \left(\frac{\gamma_{11}}{2}\Big(\frac{1}{\alpha\theta^2}\Big)\right) \times \theta^2 \times \int_0^x \Big[\frac{1}{\Gamma(\alpha)\theta^{\alpha+2}} y^{(\alpha+2)-1} e^{\frac{-y}{\theta}}\Big] dy \\
&= c \times \left(\frac{\gamma_{11}}{2}\Big(\frac{1}{\alpha\theta^2}\Big)\right) \times \theta^2 \times \frac{\Gamma(\alpha+2)}{\Gamma(\alpha)} \times \int_0^x \Big[\frac{1}{\Gamma(\alpha+2)\theta^{\alpha+2}} y^{(\alpha+2)-1} e^{\frac{-y}{\theta}}\Big] dy \\
&= c \times \left(\frac{\gamma_{11}}{2}\Big(\frac{1}{\alpha\theta^2}\Big)\right) \times \theta^2 \times \frac{\Gamma(\alpha+2)}{\Gamma(\alpha)} \times F_{v_2}(x)
\end{aligned}$$

Exponential distribution Next, we consider when $f_y(y) \sim Exponential(\frac{1}{\theta})$. To find the appropriate CDF function under this exponential base, we make note of the relationship between the exponential and gamma densities.

$$y \sim Exponetial\Big(\frac{1}{\theta}\Big) \iff y \sim \Gamma(\alpha = 1, \theta \geq 0);$$

$$f_y(y) = \frac{1}{\theta} e^{\frac{-y}{\theta}} = \frac{1}{\Gamma(1)\theta^1} y^{1-1} e^{\frac{-y}{\theta}}; y, \theta \geq 0$$

- $\mu = E[y] = \theta$, and $\sigma^2 = Var(y) = \theta^2$.

$$\begin{aligned} g_y(y) &= \Big[1 + \frac{1}{2}\,\mathrm{tr}(\mathbf{\Gamma})\Big]^{-1} \Big[\frac{1}{\Gamma(1)\theta^1} y^{1-1} e^{\frac{-y}{\theta}}\Big] \Big(1 + \frac{\gamma_{11}}{2}\left[\frac{(y-\mu)^2}{\sigma^2}\right] + \frac{1}{2}\sum_{j=2}^{d} \gamma_{jj}\Big) \\ &= \Big[1 + \frac{1}{2}\,\mathrm{tr}(\mathbf{\Gamma})\Big]^{-1} \Big[\frac{1}{\Gamma(1)\theta^1} y^{1-1} e^{\frac{-y}{\theta}}\Big] \left(\Big(1 + \frac{1}{2}\sum_{j=2}^{d} \gamma_{jj}\Big) + \frac{\gamma_{11}}{2}\left[\frac{y^2 - 2y\mu + \mu^2}{\sigma^2}\right]\right) \\ &= c \times f_y(y)\Big[\Big(c_0\Big) + \Big(c_1\Big) y + \Big(c_2\Big) y_2^2\Big] \end{aligned}$$

- Here $c_0 = \left(1 + \frac{\gamma_{11}}{2}\left(\frac{\mu^2}{\sigma^2}\right) + \frac{1}{2}\sum_{j=2}^{d} \gamma_{jj}\right) = \left(1 + \frac{\gamma_{11}}{2} + \frac{1}{2}\sum_{j=2}^{d} \gamma_{jj}\right)$
- $c_1 = \frac{\gamma_{11}}{2}\left(\frac{-2\mu}{\sigma^2}\right) = \frac{\gamma_{11}}{2}\left(\frac{-2}{\theta}\right)$
- $c_2 = \frac{\gamma_{11}}{2}\left(\frac{1}{\sigma^2}\right) = \frac{\gamma_{11}}{2}\left(\frac{1}{\theta^2}\right)$

$y \sim \text{Exponential}(\frac{1}{\theta}) = \Gamma(\alpha = 1, \theta)$, with CDF $F_Y(Y = x) = P(Y \leq x)$

$$\begin{aligned} \textbf{term1} &= c \times (c_0) \int_0^x f_y(y) dy \\ &= c \times (c_0) \times F_y(x) \\ &= c \times \left(1 + \frac{\gamma_{11}}{2} + \frac{1}{2}\sum_{j=2}^{d} \gamma_{jj}\right) \times F_y(x) \end{aligned}$$

Define a new random variable $v_1 \sim \Gamma(\alpha+1, \theta) = \Gamma(2, \theta)$, with CDF $F_{v_1}(x) = P(v_1 \leq x)$.

$$\begin{aligned}
\textbf{term2} &= c \times (c_1) \times \int_0^x y f_y(y) dy \\
&= c \times \left(\frac{\gamma_{11}}{2} \left(\frac{-2}{\theta} \right) \right) \times \int_0^x y f_y(y) dy \\
&= c \times \left(\frac{\gamma_{11}}{2} \left(\frac{-2}{\theta} \right) \right) \times \int_0^x \left[\frac{1}{\Gamma(1)\theta^1} y^{(1+1)-1} e^{\frac{-y}{\theta}} \right] dy \\
&= c \times \left(\frac{\gamma_{11}}{2} \left(\frac{-2}{\theta} \right) \right) \times \frac{\theta^2}{\theta} \times \frac{\Gamma(2)}{\Gamma(1)} \times \int_0^x \left[\frac{1}{\Gamma(2)\theta^2} y^{(2)-1} e^{\frac{-y}{\theta}} \right] dy \\
&= c \times \left(\frac{\gamma_{11}}{2} \left(\frac{-2}{\theta} \right) \right) \times \theta \times \frac{\Gamma(2)}{\Gamma(1)} \times F_{v_1}(x)
\end{aligned}$$

Define another random variable $v_2 \sim \Gamma(1+2, \theta) = \Gamma(3, \theta)$; with CDF $F_{v_2}(x) = P(v_2 \leq x)$.

$$\begin{aligned}
\textbf{term 3} &= c \times (c_2) \times \int_0^x y^2 f_y(y)\, dy \\
&= c \times \left(\frac{\gamma_{11}}{2} \left(\frac{1}{\theta^2} \right) \right) \times \int_0^x y^2 f_y(y)\, dy \\
&= c \times \left(\frac{\gamma_{11}}{2} \left(\frac{1}{\theta^2} \right) \right) \times \int_0^x \left[\frac{1}{\Gamma(1)\theta^1} y^{(1+2)-1} e^{\frac{-y}{\theta}} \right] dy \\
&= c \times \left(\frac{\gamma_{11}}{2} \left(\frac{1}{\theta^2} \right) \right) \times \frac{\theta^3}{\theta} \times \frac{\Gamma(3)}{\Gamma(1)} \times \int_0^x \left[\frac{1}{\Gamma(3)\theta^3} y^{(3)-1} e^{\frac{-y}{\theta}} \right] dy \\
&= c \times \left(\frac{\gamma_{11}}{2} \left(\frac{1}{\theta^2} \right) \right) \times \theta^2 \times \frac{\Gamma(3)}{\Gamma(1)} \times F_{v_2}(x)
\end{aligned}$$

Beta distribution Next, we consider when $f_y(y) \sim \text{Beta}(\alpha, \beta)$.

$$y \sim f(y; \alpha, \beta) = \frac{1}{B(\alpha, \beta)} y^{\alpha-1}(1-y)^{\beta-1} = \frac{\Gamma(\alpha+\beta)}{\Gamma(\alpha)\Gamma(\beta)} y^{\alpha-1}(1-y)^{\beta-1}, \quad y \in [0, 1]$$

- $\mu = E[y] = \frac{\alpha}{\alpha+\beta}$, and $\sigma^2 = Var(y) = \frac{\alpha\beta}{(\alpha+\beta)^2(\alpha+\beta+1)}$,

$$\begin{aligned}
& g_y(y) \\
= \;& \Big[1 + \frac{1}{2}\operatorname{tr}(\mathbf{\Gamma})\Big]^{-1}\left[\frac{\Gamma(\alpha+\beta)}{\Gamma(\alpha)\Gamma(\beta)} y^{\alpha-1}(1-y)^{\beta-1}\right]\Big(1 + \frac{\gamma_{11}}{2}\left[\frac{(y-\mu)^2}{\sigma^2}\right] + \frac{1}{2}\sum_{j=2}^{m}\gamma_{jj}\Big) \\
= \;& \Big[1 + \frac{1}{2}\operatorname{tr}(\mathbf{\Gamma})\Big]^{-1}\left[\frac{\Gamma(\alpha+\beta)}{\Gamma(\alpha)\Gamma(\beta)} y^{\alpha-1}(1-y)^{\beta-1}\right]\left(\Big(1 + \frac{1}{2}\sum_{j=2}^{d}\gamma_{jj}\Big) + \frac{\gamma_{11}}{2}\left[\frac{y^2 - 2y\mu + \mu^2}{\sigma^2}\right]\right) \\
= \;& c \times f_y(y)\Big[\Big(c_0\Big) + \Big(c_1\Big)y + \Big(c_2\Big)y_2^2\Big]
\end{aligned}$$

- Here $c_0 = \left(1 + \frac{\gamma_{11}}{2}\left(\frac{\mu^2}{\sigma^2}\right) + \frac{1}{2}\sum_{j=2}^{d}\gamma_{jj}\right) = \left(1 + \frac{\gamma_{11}}{2}\left(\frac{\alpha(\alpha+\beta+1)}{\beta}\right) + \frac{1}{2}\sum_{j=2}^{d}\gamma_{jj}\right)$
- $c_1 = \frac{\gamma_{11}}{2}\left(\frac{-2\mu}{\sigma^2}\right) = \frac{\gamma_{11}}{2}\left(\frac{-2(\alpha+\beta)(\alpha+\beta+1)}{\beta}\right)$
- $c_2 = \frac{\gamma_{11}}{2}\left(\frac{1}{\sigma^2}\right) = \frac{\gamma_{11}}{2}\left(\frac{(\alpha+\beta)^2(\alpha+\beta+1)}{\alpha\beta}\right)$

$y \sim \text{Beta}(\alpha, \beta)$ with CDF $F_Y(Y = x) = P(Y \leq x)$

$$\begin{aligned}
\textbf{term1} &= c \times (c_0)\int_0^x f_y(y)dy \\
&= c \times (c_0) \times F_y(x) \\
&= c \times \left(1 + \frac{\gamma_{11}}{2}\left(\frac{\alpha(\alpha+\beta+1)}{\beta}\right) + \frac{1}{2}\sum_{j=2}^{d}\gamma_{jj}\right) \times F_y(x)
\end{aligned}$$

Define a new random variable $v_1 \sim \text{Beta}(\alpha+1, \beta)$ with CDF $F_{v_1}(x) = P(v_1 \leq x)$.

$$
\begin{aligned}
\textbf{term2} &= c \times (c_1) \times \int_0^x y f_y(y) dy \\
&= c \times \frac{\gamma_{11}}{2} \Big(\frac{-2(\alpha+\beta)(\alpha+\beta+1)}{\beta} \Big) \times \int_0^x y f_y(y) dy \\
&= c \times \frac{\gamma_{11}}{2} \Big(\frac{-2(\alpha+\beta)(\alpha+\beta+1)}{\beta} \Big) \times \int_0^x \left[\frac{\Gamma(\alpha+\beta)}{\Gamma(\alpha)\Gamma(\beta)} y^{(\alpha+1)-1} (1-y)^{\beta-1} \right] dy \\
&= c \times \frac{\gamma_{11}}{2} \Big(\frac{-2(\alpha+\beta)(\alpha+\beta+1)}{\beta} \Big) \times \frac{\Gamma(\alpha+\beta)}{\Gamma(\alpha)} \frac{\Gamma(\alpha+1)}{\Gamma(\alpha+\beta+1)} \times \int_0^x f_{v_1}(y) dy \\
&= c \times \frac{\gamma_{11}}{2} \Big(\frac{-2(\alpha+\beta)(\alpha+\beta+1)}{\beta} \Big) \times \frac{\Gamma(\alpha+\beta)}{\Gamma(\alpha)} \frac{\Gamma(\alpha+1)}{\Gamma(\alpha+\beta+1)} \times F_{v_1}(x)
\end{aligned}
$$

Define another random variable $v_2 \sim \text{Beta}(\alpha+2, \beta)$ with CDF $F_{v_2}(x) = P(v_2 \leq x)$.

$$
\begin{aligned}
\textbf{term 3} &= c \times (c_2) \times \int_0^x y^2 f_y(y)\, dy \\
&= c \times \frac{\gamma_{11}}{2} \Big(\frac{(\alpha+\beta)^2(\alpha+\beta+1)}{\alpha\beta} \Big) \times \int_0^x y^2 f_y(y)\, dy \\
&= c \times \frac{\gamma_{11}}{2} \Big(\frac{(\alpha+\beta)^2(\alpha+\beta+1)}{\alpha\beta} \Big) \times \int_0^x \left[\frac{\Gamma(\alpha+\beta)}{\Gamma(\alpha)\Gamma(\beta)} y^{(\alpha+2)-1} (1-y)^{\beta-1} \right] dy \\
&= c \times \frac{\gamma_{11}}{2} \Big(\frac{-2(\alpha+\beta)(\alpha+\beta+2)}{\beta} \Big) \times \frac{\Gamma(\alpha+\beta)}{\Gamma(\alpha)} \frac{\Gamma(\alpha+2)}{\Gamma(\alpha+2+\beta)} \times \int_0^x f_{v_2}(y) dy \\
&= c \times \frac{\gamma_{11}}{2} \Big(\frac{(\alpha+\beta)^2(\alpha+\beta+2)}{\alpha\beta} \Big) \times \frac{\Gamma(\alpha+\beta)}{\Gamma(\alpha)} \frac{\Gamma(\alpha+2)}{\Gamma(\alpha+2+\beta)} \times F_{v_2}(x)
\end{aligned}
$$

S1.2.4 Discrete Outcomes

When the densities $f_i(y_i)$ are discrete, it may be necessary to compute infinite sums involving these probabilities. For example, such sums occur naturally in numerical algorithms developed for Poisson, Geometric, negative binomial variate generation. From a practical standpoint, it is necessary to truncate these infinite sums after a finite number of terms.

Consider any random variable Z with nonnegative integer values, discrete density $p_i = \Pr(Z = i)$, and mean ν. The inverse method of random sampling reduces to a sequence of comparisons. We partition the interval $[0, 1]$ into subintervals with the ith subinterval of length p_i. To sample Z, we draw a uniform random deviate U from

[0, 1] and return the deviate j determined by the conditions $\sum_{i=1}^{j-1} p_i \leq U < \sum_{i=1}^{j} p_i$. There is no need to invoke the distribution of Z. The process is most efficient when the largest p_i occur first. This suggests that we let k denote the least integer $\lfloor \nu \rfloor$ and rearrange the probabilities in the order $p_k, p_{k+1}, p_{k-1}, p_{k+2}, p_{k-2}, \ldots$ This tactic is apt put most of the probability mass first and render sampling efficient.

Poisson Distribution A Poisson distribution describes the number of independent events occurring within a unit time interval, given the average rate of occurrence θ.

$$y \sim \text{Poisson}(\theta); f_y(y) = \frac{\theta^y e^{-\theta y}}{y!}, y = 0, 1, 2, 3, ...$$

- $\mu = E[y] = \theta = \sigma^2 = Var(y)$

$$\begin{aligned} g_y(y) &= \left[1 + \frac{1}{2}\operatorname{tr}(\mathbf{\Gamma})\right]^{-1} \left[\frac{\theta^y e^{-\theta y}}{y!}\right] \left(1 + \frac{\gamma_{11}}{2}\left[\frac{(y-\mu)^2}{\sigma^2}\right] + \frac{1}{2}\sum_{j=2}^{d} \gamma_{jj}\right) \\ &= \left[1 + \frac{1}{2}\operatorname{tr}(\mathbf{\Gamma})\right]^{-1} \left[\frac{\theta^y e^{-\theta y}}{y!}\right] \left(\left(1 + \frac{1}{2}\sum_{j=2}^{d} \gamma_{jj}\right) + \frac{\gamma_{11}}{2}\left[\frac{y^2 - 2y\mu + \mu^2}{\sigma^2}\right]\right) \\ &= c \times f_y(y)\left[\left(c_0\right) + \left(c_1\right)y + \left(c_2\right)y_2^2\right] \end{aligned}$$

- Here $c_0 = \left(1 + \frac{\gamma_{11}}{2}\left(\frac{\mu^2}{\sigma^2}\right) + \frac{1}{2}\sum_{j=2}^{m} \gamma_{jj}\right) = \left(1 + \frac{\gamma_{11}}{2}\left(1\right) + \frac{1}{2}\sum_{j=2}^{d} \gamma_{jj}\right)$

- $c_1 = \left(\frac{\gamma_{11}}{2}\left(\frac{-2\mu}{\sigma^2}\right)\right) = \left(\frac{\gamma_{11}}{2}\left(-2\right)\right)$

- $c_2 = \left(\frac{\gamma_{11}}{2}\left(\frac{1}{\sigma^2}\right)\right) = \left(\frac{\gamma_{11}}{2}\left(\frac{1}{\theta}\right)\right)$

Binomial Distribution A Binomial distribution characterizes the number of successes in a sequence of independent trials. It has two parameters: N, the number of trials, and p, the probability of success in an individual trial, with the distribution:

$$y \sim \text{Binomial}(N, p), \quad f_y(y) = \binom{N}{y} p^y (1-p)^{N-y}, \quad y = 0, 1, 2, ..., N$$

- $\mu = E[y] = Np; \sigma^2 = Var(y) = Np(1-p)$

$$\begin{aligned} g_y(y) &= \Big[1+\frac{1}{2}\,\mathrm{tr}(\boldsymbol{\Gamma})\Big]^{-1}\Big[\binom{N}{y}p^y(1-p)^{N-y}\Big]\Big(1+\frac{\gamma_{11}}{2}\left[\frac{(y-\mu)^2}{\sigma^2}\right]+\frac{1}{2}\sum_{j=2}^{m}\gamma_{jj}\Big) \\ &= \Big[1+\frac{1}{2}\,\mathrm{tr}(\boldsymbol{\Gamma})\Big]^{-1}\Big[\binom{N}{y}p^y(1-p)^{N-y}\Big]\left(\Big(1+\frac{1}{2}\sum_{j=2}^{m}\gamma_{jj}\Big)+\frac{\gamma_{11}}{2}\left[\frac{y^2-2y\mu+\mu^2}{\sigma^2}\right]\right) \\ &= c\times f_y(y)\Big[\Big(c_0\Big)+\Big(c_1\Big)y+\Big(c_2\Big)y^2_2\Big] \end{aligned}$$

- Here $c_0 = \left(1+\frac{\gamma_{11}}{2}\left(\frac{\mu^2}{\sigma^2}\right)+\frac{1}{2}\sum_{j=2}^{m}\gamma_{jj}\right) = \left(1+\frac{\gamma_{11}}{2}\left(\frac{(Np)^2}{Np(1-p)}\right)+\frac{1}{2}\sum_{j=2}^{m}\gamma_{jj}\right)$
- $c_1 = \left(\frac{\gamma_{11}}{2}\left(\frac{-2\mu}{\sigma^2}\right)\right) = \left(\frac{\gamma_{11}}{2}\left(\frac{-2Np}{Np(1-p)}\right)\right)$
- $c_2 = \left(\frac{\gamma_{11}}{2}\left(\frac{1}{\sigma^2}\right)\right) = \left(\frac{\gamma_{11}}{2}\left(\frac{1}{Np(1-p)}\right)\right)$

Geometric Distribution A Geometric distribution characterizes the number of failures before the first success in a sequence of independent Bernoulli trials with success rate ‘p‘.

$$y \sim \text{Geometric}(p); f_y(y) = (1-p)^y p, y = 0, 1, 2, ...$$

- $\mu = E[y] = \frac{1}{p}; \sigma^2 = Var(y) = \frac{1-p}{p^2}$

$$\begin{aligned} g_y(y) &= \Big[1+\frac{1}{2}\,\mathrm{tr}(\boldsymbol{\Gamma})\Big]^{-1}\Big[(1-p)^y p\Big]\Big(1+\frac{\gamma_{11}}{2}\left[\frac{(y-\mu)^2}{\sigma^2}\right]+\frac{1}{2}\sum_{j=2}^{d}\gamma_{jj}\Big) \\ &= \Big[1+\frac{1}{2}\,\mathrm{tr}(\boldsymbol{\Gamma})\Big]^{-1}\Big[(1-p)^y p\Big]\left(\Big(1+\frac{1}{2}\sum_{j=2}^{d}\gamma_{jj}\Big)+\frac{\gamma_{11}}{2}\left[\frac{y^2-2y\mu+\mu^2}{\sigma^2}\right]\right) \\ &= c\times f_y(y)\Big[\Big(c_0\Big)+\Big(c_1\Big)y+\Big(c_2\Big)y^2_2\Big] \end{aligned}$$

- Here $c_0 = \left(1 + \frac{\gamma_{11}}{2}\left(\frac{\mu^2}{\sigma^2}\right) + \frac{1}{2}\sum_{j=2}^{d}\gamma_{jj}\right) = \left(1 + \frac{\gamma_{11}}{2}\left(\frac{\frac{1}{p}}{\frac{1-p}{p^2}}\right) + \frac{1}{2}\sum_{j=2}^{d}\gamma_{jj}\right)$

- $c_1 = \left(\frac{\gamma_{11}}{2}\left(\frac{-2\mu}{\sigma^2}\right)\right) = \left(\frac{\gamma_{11}}{2}\left(\frac{\frac{-2}{p}}{\frac{1-p}{p^2}}\right)\right)$

- $c_2 = \left(\frac{\gamma_{11}}{2}\left(\frac{1}{\sigma^2}\right)\right) = \left(\frac{\gamma_{11}}{2}\left(\frac{p^2}{1-p}\right)\right)$

Negative Binomial Distribution A negative binomial distribution describes the number of failures before the 'r'th success in a sequence of independent Bernoulli trials. It is parameterized by 'r', the number of successes, and 'p', the probability of success in an individual trial.

$$y \sim \text{Negative Binomial}(r, p); f_y(y) = \binom{y+r-1}{y} p^r (1-p)^y, y = 0, 1, 2, ...$$

- $\mu = E[y] = \frac{pr}{1-p}; \sigma^2 = Var(y) = \frac{pr}{(1-p)^2}$

$$\begin{aligned} g_y(y) &= \left[1 + \frac{1}{2}\operatorname{tr}(\mathbf{\Gamma})\right]^{-1}\left[\binom{y+r-1}{y} p^r (1-p)^y\right]\left(1 + \frac{\gamma_{11}}{2}\left[\frac{(y-\mu)^2}{\sigma^2}\right] + \frac{1}{2}\sum_{j=2}^{d}\gamma_{jj}\right) \\ &= \left[1 + \frac{1}{2}\operatorname{tr}(\mathbf{\Gamma})\right]^{-1}\left[\binom{y+r-1}{y} p^r (1-p)^y\right]\left(\left(1 + \frac{1}{2}\sum_{j=2}^{d}\gamma_{jj}\right) + \frac{\gamma_{11}}{2}\left[\frac{y^2 - 2y\mu + \mu^2}{\sigma^2}\right]\right) \\ &= c \times f_y(y)\left[\left(c_0\right) + \left(c_1\right)y + \left(c_2\right)y_2^2\right] \end{aligned}$$

- Here $c_0 = \left(1 + \frac{\gamma_{11}}{2}\left(\frac{\mu^2}{\sigma^2}\right) + \frac{1}{2}\sum_{j=2}^{d}\gamma_{jj}\right) = \left(1 + \frac{\gamma_{11}}{2}\left(\frac{\frac{pr}{1-p}}{\frac{pr}{(1-p)^2}}\right) + \frac{1}{2}\sum_{j=2}^{d}\gamma_{jj}\right)$

- $c_1 = \left(\frac{\gamma_{11}}{2}\left(\frac{-2\mu}{\sigma^2}\right)\right) = \left(\frac{\gamma_{11}}{2}\left(\frac{\frac{-2pr}{1-p}}{\frac{pr}{(1-p)^2}}\right)\right)$

- $c_2 = \left(\frac{\gamma_{11}}{2}\left(\frac{1}{\sigma^2}\right)\right) = \left(\frac{\gamma_{11}}{2}\left(\frac{(1-p)^2}{pr}\right)\right)$

S1.3 Parameter Estimation:

We extend the Gaussian Base Model to accommodate densities in exponential family of distributions under the generalized linear model (GLM) framework. In this note, we pay close attention to the density-specific quantities which facilitate parameter estimation, and illustrate using the Poisson and Bernoulli density.

S1.3.1 Fisher Scoring to Estimate Beta

The loglikelihood is

$$\begin{aligned}\mathcal{L}(\boldsymbol{\beta}) &= -\sum_{i=1}^{n} \ln\left[1+\frac{1}{2}\,\mathrm{tr}(\boldsymbol{\Gamma}_i)\right] + \sum_{i=1}^{n} \ln\left\{1+\frac{1}{2}\boldsymbol{r}_i(\boldsymbol{\beta})^\top \boldsymbol{\Gamma}_i \boldsymbol{r}_i(\boldsymbol{\beta})\right\} \\ &\quad + \sum_{i=1}^{n}\sum_{j=1}^{n_i} \ln f_{ij}(y_{ij} \mid \boldsymbol{\beta}).\end{aligned} \tag{3}$$

For each distribution, the objective function is the loglikelihood (3), and can be viewed as three separate pieces. The last term of the loglikelihood is specific to the hypothesized density, and has first derivative, $\sum_{i=1}^{n}\sum_j \nabla \ln f_{ij}(y_{ij} \mid \boldsymbol{\beta})$, and second derivative $d^2L_n(\boldsymbol{\beta})$, that generalize to the exponential family of distributions.

The score (gradient of the loglikelihood) with respect to $\boldsymbol{\beta}$ is:

$$\nabla L_n(\boldsymbol{\beta}) = \sum_{i=1}^{n}\sum_{j} \nabla \ln f_{ij}(y_{ij} \mid \boldsymbol{\beta}) + \sum_{i=1}^{n} \frac{\nabla r_i(\boldsymbol{\beta})\boldsymbol{\Gamma}_i r_i(\boldsymbol{\beta})}{1+\frac{1}{2}r_i(\boldsymbol{\beta})^\top \boldsymbol{\Gamma}_i r_i(\boldsymbol{\beta})}, \tag{4}$$

The first term in the gradient, $\sum_{i=1}^{n}\sum_j \nabla \ln f_{ij}(y_{ij} \mid \boldsymbol{\beta})$, corresponds to the first derivative of the piece of the loglikelihood, specific to the hypothesized density. We can write this first term as a function of $\mathbf{W_{1i}}$, a diagonal matrix of "working weights".

$$\sum_{i=1}^{n}\sum_{j} \nabla \ln f_{ij}(y_{ij} \mid \boldsymbol{\beta}) \;=\; \sum_{i=1}^{n}\sum_{j=1}^{d_i} \frac{(y_{ij}-\mu_{ij})\mu'_{ij}(\eta_{ij})}{\sigma^2_{ij}}\mathbf{x}_{ij} = \sum_{i=1}^{n} \mathbf{X_i}^\top \mathbf{W_{1i}}(\mathbf{Y_i}-\boldsymbol{\mu_i})$$

$$\boldsymbol{W_{1i}} = \mathbf{Diagonal}\left(\frac{\mathbf{g}'(\mathbf{X_i}^\top\boldsymbol{\beta})}{\mathbf{var}(\mathbf{Y_i}|\mu_\mathbf{i})}\right) = \begin{pmatrix} \frac{\mu'_{i1}(\eta_{i1})}{\sigma^2_{i1}} & 0 & \cdots & 0 \\ 0 & \frac{\mu'_{i2}(\eta_{i2})}{\sigma^2_{i2}} & \cdots & 0 \\ \vdots & \vdots & \ddots & \vdots \\ 0 & 0 & \cdots & \frac{\mu'_{in_i}(\eta_{in_i})}{\sigma^2_{in_i}} \end{pmatrix}$$

As discussed in the main text, we can use the expected Fisher Information to get an approximation of the Hessian, which is clearly negative semi-definite.

$$-\sum_{i=1}^{n} \mathbf{X_i}^\top \mathbf{W_{2i}} \mathbf{X_i} - \sum_{i=1}^{n} \frac{[\nabla \boldsymbol{r}_i(\boldsymbol{\beta})^\top \boldsymbol{\Gamma}_i \boldsymbol{r}_i(\boldsymbol{\beta})][\nabla \boldsymbol{r}_i(\boldsymbol{\beta})^\top \boldsymbol{\Gamma}_i \boldsymbol{r}_i(\boldsymbol{\beta})]^\top}{\left[1 + \frac{1}{2}\boldsymbol{r}_i(\boldsymbol{\beta})^\top \boldsymbol{\Gamma}_i \boldsymbol{r}_i(\boldsymbol{\beta})\right]^2} \tag{5}$$

Specifically, we approximate the second derivative of the piece of the loglikelihood particular to the hypothesized density, $d^2 L_n(\beta)$. Using the Expected Fisher Information Matrix, we present this term as a function of another diagonal weight matrix, $\mathbf{W_{2i}}$.

$$\begin{aligned} d^2 L_n(\beta) &= \sum_{i=1}^{n}\sum_{j=1}^{n} \frac{[\mu'_{ij}(\eta_{ij})]^2}{\sigma_{ij}^2} \mathbf{x}_{ij}\mathbf{x}_{ij}^\top - \sum_{i=1}^{n}\sum_{j=1}^{n} \frac{(y_{ij}-\mu_{ij})\mu''_{ij}(\eta_{ij})}{\sigma_{ij}^2} \mathbf{x}_{ij}\mathbf{x}_{ij}^\top \\ &\quad + \sum_{i=1}^{n}\sum_{j=1}^{n} \frac{(y_{ij}-\mu_{ij})[\mu'_{ij}(\eta_{ij})]^2 (d\sigma_{ij}^2/d\mu_{ij})}{\sigma_{ij}^4} \mathbf{x}_{ij}\mathbf{x}_{ij}^\top \\ \mathbf{FIM}_n(\beta) &= \mathbf{E}[-d^2 L_n(\beta)] = -\sum_{i=1}^{n}\sum_{j=1}^{d_i} \frac{[\mu'_{ij}(\eta_{ij})]^2}{\sigma_{ij}^2} \mathbf{x}_{ij}\mathbf{x}_{ij}^\top = -\sum_{i=1}^{n} \mathbf{X_i}^\top \mathbf{W_{2i}} \mathbf{X_i}. \end{aligned}$$

$$\boldsymbol{W_{2i}} = \mathbf{Diagonal}\left(\frac{\mathbf{g'(X_i^\top \boldsymbol{\beta})^2}}{\mathbf{var(Y_i|\mu_i)}}\right) = \begin{pmatrix} \frac{(\mu'_{i1}(\eta_{i1}))^2}{\sigma_{i1}^2} & 0 & \cdots & 0 \\ 0 & \frac{(\mu'_{i2}(\eta_{i2}))^2}{\sigma_{i2}^2} & \cdots & 0 \\ \vdots & \vdots & \ddots & \vdots \\ 0 & 0 & \cdots & \frac{(\mu'_{in_i}(\eta_{in_i}))^2}{\sigma_{in_i}^2} \end{pmatrix}$$

The score and approximate Hessian provide the ingredients for a kind of scoring algorithm for improving $\boldsymbol{\beta}$ in our model. For each Newton update of the fixed effect parameter, $\boldsymbol{\beta}$, in addition to updating the residual vector, $r_i(\boldsymbol{\beta})$, we must also update these weight matrices, $\boldsymbol{W_{1i}}$ and $\boldsymbol{W_{2i}}$, in the update of the Score and Hessian. We can find these quantities easily by making the appropriate calls to the GLM package, GLM.jl.

Let y_{ij} represent the jth outcome for person i, hypothesized to come from a non-normal density in the exponential family of distributions, $f_{ij}(y_{ij} \mid \boldsymbol{\beta})$. For each hypothesized density under the GLM framework, we have mean parameter $\mu_{ij}(\boldsymbol{\beta}) = g^{[-1]}(\eta_{ij}(\boldsymbol{\beta})) = g^{[-1]}(\mathbf{x_{ij}}\boldsymbol{\beta})$, and variance parameter $\sigma_{ij}^2(\boldsymbol{\beta})$. Using these quantities, we define $r_{ij}(\boldsymbol{\beta}), j \in [1, d_i]$ as the jth entry in the standardized residual vector for observation or group i.

$$r_{ij}(\boldsymbol{\beta}) = \sqrt{\tau}(y_{ij} - \mu_{ij}(\boldsymbol{\beta})) = \frac{(y_{ij} - \mu_{ij}(\boldsymbol{\beta}))}{\sqrt{\sigma^2_{ij}(\boldsymbol{\beta})}} \in \mathbb{R} \tag{6}$$

Let $\nabla r_i(\boldsymbol{\beta}) \in \mathbb{R}^{d_i \times p}$ denote the matrix of differentials of all d_i observations for the ith individuals standardized residual vector $r_i(\boldsymbol{\beta})$. This quantity is important for our score and hessian computation, which really helps the optimization algorithm to speed up convergence.

$$\nabla r_i(\boldsymbol{\beta})^\top \quad = \begin{pmatrix} \nabla r_{i1}(\boldsymbol{\beta}) & \nabla r_{i2}(\boldsymbol{\beta}) & ... & \nabla r_{id_i}(\boldsymbol{\beta}) & \end{pmatrix}$$

For each of the $j \in [1, d_i]$ observations for the ith individual, $\nabla r_i(\boldsymbol{\beta})^\top$ can be formed column by column, where $\nabla r_{ij}(\boldsymbol{\beta})$ denotes the jth column. $\nabla \mu_{ij}(\boldsymbol{\beta})$ and $\nabla \sigma^2_{ij}(\boldsymbol{\beta})$ respectively reflect the derivative of the mean and variance of the hypothesized density, with respect to $\boldsymbol{\beta}$.

$$\nabla r_{ij}(\boldsymbol{\beta}) \quad = \quad -\frac{1}{\sigma_{ij}(\boldsymbol{\beta})}\nabla \mu_{ij}(\boldsymbol{\beta}) - \frac{1}{2}\frac{y_{ij} - \mu_{ij}(\boldsymbol{\beta})}{\sigma^3_{ij}(\boldsymbol{\beta})}\nabla \sigma^2_{ij}(\boldsymbol{\beta}) \in \mathbb{R}^p \tag{7}$$

$$\nabla \mu_{ij}(\boldsymbol{\beta}) = \frac{\partial \mu_{ij}(\boldsymbol{\beta})}{\partial \eta_{ij}(\boldsymbol{\beta})} * \frac{\partial \eta_{ij}(\boldsymbol{\beta})}{\partial \boldsymbol{\beta}} = \begin{pmatrix} \frac{\partial \mu_{ij}(\boldsymbol{\beta})}{\partial \eta_{ij}(\boldsymbol{\beta})} * \frac{\partial \mathbf{x_{ij}}\boldsymbol{\beta}}{\partial \beta_1} \\ \vdots \\ \frac{\partial \mu_{ij}(\boldsymbol{\beta})}{\partial \eta_{ij}(\boldsymbol{\beta})} * \frac{\partial \mathbf{x_{ij}}\boldsymbol{\beta}}{\partial \beta_p} \end{pmatrix} = \frac{\partial \mu_{ij}(\boldsymbol{\beta})}{\partial \eta_{ij}(\boldsymbol{\beta})} * \begin{pmatrix} x_{ij_1} \\ x_{ij_2} \\ \vdots \\ x_{ij_p} \end{pmatrix} = \frac{\partial \mu_{ij}(\boldsymbol{\beta})}{\partial \eta_{ij}(\boldsymbol{\beta})} * \mathbf{x_{ij}} \in \mathbb{R}^p$$

$$\nabla \sigma^2_{ij}(\boldsymbol{\beta}) = \frac{\partial \sigma^2_{ij}(\boldsymbol{\beta})}{\partial \mu_{ij}(\boldsymbol{\beta})}\frac{\partial \mu_{ij}(\boldsymbol{\beta})}{\partial \eta_{ij}(\boldsymbol{\beta})}\frac{\partial \eta_{ij}(\boldsymbol{\beta})}{\partial \boldsymbol{\beta}} = \frac{\partial \sigma^2_{ij}(\boldsymbol{\beta})}{\partial \mu_{ij}(\boldsymbol{\beta})}\frac{\partial \mu_{ij}(\boldsymbol{\beta})}{\partial \eta_{ij}(\boldsymbol{\beta})} * \mathbf{x_{ij}} \in \mathbb{R}^p$$

For the Gaussian base model, since the identity function is the appropriate canonical link, we have that $\mu_{ij}(\boldsymbol{\beta}) = \eta_{ij}(\boldsymbol{\beta}) = \mathbf{x_{ij}}\boldsymbol{\beta} = x_{ij_1} * \beta_1 + ... + x_{ij_p} * \beta_p$ where $\mathbf{x_{ij}}$ denotes the vector of p covariate values for j^{th} measurement of the ith person.

$$\nabla \mu_{ij}(\boldsymbol{\beta}) = \frac{\partial \eta_{ij}(\boldsymbol{\beta})}{\partial \boldsymbol{\beta}} = \begin{pmatrix} \frac{\partial \mathbf{x_{ij}}\boldsymbol{\beta}}{\partial \beta_1} \\ \vdots \\ \frac{\partial \mathbf{x_{ij}}\boldsymbol{\beta}}{\partial \beta_p} \end{pmatrix} = \begin{pmatrix} X_{ij_1} \\ X_{ij_2} \\ \vdots \\ X_{ij_p} \end{pmatrix} = \mathbf{x_{ij}} \in \mathbb{R}^p$$

$$\nabla \sigma^2_{ij}(\boldsymbol{\beta}) = \frac{\partial \sigma^2_{ij}(\boldsymbol{\beta})}{\partial \mu_{ij}(\boldsymbol{\beta})}\frac{\partial \mu_{ij}(\boldsymbol{\beta})}{\partial \eta_{ij}(\boldsymbol{\beta})}\frac{\partial \eta_{ij}(\boldsymbol{\beta})}{\partial \boldsymbol{\beta}} = 0 * 1 * \mathbf{x_{ij}} = \mathbf{0} \in \mathbb{R}^p$$

In the table below, we derive the same quantities for the Normal, Poisson, Bernoulli and negative binomial distributions, under the appropriate canonical link function. The details of the derivation for the above table is below.

Distribution	$g(\mu_{ij}(\boldsymbol{\beta})) = \eta_{ij}(\boldsymbol{\beta})$	$\mu_{ij}(\boldsymbol{\beta}) \in \mathbb{R}$	$\sigma^2_{ij}(\boldsymbol{\beta}) \in \mathbb{R}$	$\nabla \mu_{ij}(\boldsymbol{\beta}) \in \mathbb{R}^p$	$\nabla \sigma^2_{ij}(\boldsymbol{\beta}) \in \mathbb{R}^p$
Normal	Identity Link	$\eta_{ij}(\boldsymbol{\beta})$	σ^2_{ij}	$\mathbf{x_i}$	$\mathbf{0}$
Poisson	Log Link	$e^{\eta_{ij}(\boldsymbol{\beta})}$	$\mu_{ij}(\boldsymbol{\beta})$	$e^{\eta_{ij}(\boldsymbol{\beta})} * \mathbf{x_i}$	$e^{\eta_{ij}(\boldsymbol{\beta})} * \mathbf{x_{ij}}$
Bernoulli	Logit Link	$\frac{e^{\eta_{ij}(\boldsymbol{\beta})}}{1+e^{\eta_{ij}(\boldsymbol{\beta})}}$	$\frac{e^{\eta_{ij}(\boldsymbol{\beta})}}{(1+e^{\eta_{ij}(\boldsymbol{\beta})})^2}$	$\frac{e^{\eta_{ij}(\boldsymbol{\beta})})}{(1+e^{\eta_{ij}(\boldsymbol{\beta})})^2} * \mathbf{x_{ij}}$	$\frac{e^{\eta_{ij}(\boldsymbol{\beta})}(1-e^{\eta_{ij}(\boldsymbol{\beta})})^2}{(1+e^{\eta_{ij}(\boldsymbol{\beta})})^2} * \mathbf{x_i}$
Negative Binomial	Log Link	$e^{\eta_{ij}(\boldsymbol{\beta})}$	$e^{\eta_{ij}(\boldsymbol{\beta})} * (1 + \frac{e^{\eta_{ij}(\boldsymbol{\beta})}}{r})$	$e^{\eta_{ij}(\boldsymbol{\beta})} * \mathbf{x_i}$	$(\frac{e^{\eta_{ij}(\boldsymbol{\beta})}}{r} + (1 + \frac{e^{\eta_{ij}(\boldsymbol{\beta})}}{r})) * e^{\eta_{ij}(\boldsymbol{\beta})} * \mathbf{x_i}$

S1.3.2 MM Algorithm for the VC Model Parameters

One can construct an iterative MM algorithm for updating $\boldsymbol{\theta}$ holding $\boldsymbol{\beta}$ fixed. There exists a substantial literature on the MM principle for optimization (Lange et al., 2000; Lange, 2016; Zhou et al., 2019). The idea in maximization is to concoct a surrogate function $g(\boldsymbol{\theta} \mid \boldsymbol{\theta}_r)$ that is easy to maximize and hugs the objective $f(\boldsymbol{\theta})$ tightly. Here $\boldsymbol{\theta}_r$ is the current value of $\boldsymbol{\theta}$. Construction of the surrogate is guided by two minorization requirements:

$$\begin{aligned} f(\boldsymbol{\theta}) &\geq g(\boldsymbol{\theta} \mid \boldsymbol{\theta}_r) \quad \forall \ \boldsymbol{\theta} \quad \text{(dominance condition)} \\ f(\boldsymbol{\theta}_r) &= g(\boldsymbol{\theta}_r \mid \boldsymbol{\theta}_r) \quad \text{(tangent condition)}. \end{aligned}$$

The next iterate is determined by $\boldsymbol{\theta}_{r+1} = \text{argmax } g(\boldsymbol{\theta} \mid \boldsymbol{\theta}_r)$. The MM principle guarantees that $f(\boldsymbol{\theta}_{r+1}) \geq f(\boldsymbol{\theta}_r)$, with strict inequality being the rule. In practice, minorization is carried out piecemeal on a sum of terms defining the objective.

To update the variance components $\boldsymbol{\theta} = \{\theta_k, k \in [1, m]\}$, the relevant part of the loglikelihood is

$$f(\boldsymbol{\theta}) = \sum_{i=1}^{n} \ln(1 + \boldsymbol{\theta}^\top \boldsymbol{b}_i) - \sum_{i=1}^{n} \ln(1 + \boldsymbol{\theta}^\top \boldsymbol{c}_i) \tag{8}$$

by defining the vectors $\boldsymbol{b}_i$ and $\boldsymbol{c}_i$ with nonnegative components

$$\begin{aligned} \boldsymbol{b}_{ik} &= \frac{1}{2} r_i(\boldsymbol{\beta})^\top \boldsymbol{\Omega}_{ik} r_i(\boldsymbol{\beta}) \\ \boldsymbol{c}_{ik} &= \frac{1}{2} \operatorname{tr}(\boldsymbol{\Omega}_{ik}). \end{aligned}$$

Jensen's inequality gives the minorization for the first term

$$\begin{aligned} \sum_{i=1}^{n} \ln(1 + \boldsymbol{\theta}^\top \boldsymbol{b}_i) &\geq \sum_{i=1}^{n} \frac{1}{1 + \boldsymbol{\theta}_r^\top \boldsymbol{b}_i} \ln\left(\frac{1 + \boldsymbol{\theta}_r^\top \boldsymbol{b}_i}{1} 1\right) \\ &\quad + \sum_{i=1}^{n} \sum_{j} \frac{\theta_{rj} b_{ij}}{1 + \boldsymbol{\theta}_r^\top \boldsymbol{b}_i} \ln\left(\frac{1 + \boldsymbol{\theta}_r^\top \boldsymbol{b}_i}{\theta_{rj} b_{ij}} \theta_j b_{ij}\right). \end{aligned}$$

For the second term, we capitalize on the convexity of $-\ln(s)$. The supporting hyperplane inequality implies the linear minorization

$$-\sum_{i=1}^{n} \ln(1+\boldsymbol{\theta}^\top \boldsymbol{c}_i) \quad \geq \quad -\sum_{i=1}^{n} \frac{1}{1+\boldsymbol{\theta}_r^\top \boldsymbol{c}_i}(1+\boldsymbol{\theta}^\top \boldsymbol{c}_i - 1 - \boldsymbol{\theta}_r^\top \boldsymbol{c}_i).$$

The sum of these two minorizations constitutes the overall minorization $g(\boldsymbol{\theta} \mid \boldsymbol{\theta}_r)$. The stationary condition $\nabla g(\boldsymbol{\theta} \mid \boldsymbol{\theta}_r) = \mathbf{0}$ can be solved to yield the updates

$$\theta_{r+1,j} \quad = \quad \theta_{rj} \frac{\sum_{i=1}^{n} \frac{b_{ri}}{1+\boldsymbol{\theta}_r^\top \boldsymbol{b}_i}}{\sum_{i=1}^{n} \frac{c_{ij}}{1+\boldsymbol{\theta}_r^\top \boldsymbol{c}_i}}.$$

Note that the update $\theta_{r+1,j}$ remains nonnegative if θ_{rj} is nonnegative and equals 0 if and only if $\theta_{rj} = 0$. However, convergence of θ_{rj} to 0 is possible. More importantly, the MM updates drive the loglikelihood uphill.

S1.3.3 Quasi-Newton Algorithm

Alternatively, we can estimate the mean and variance parameters joinly using the Quasi-Newton algorithm.

Score and Hessian For the AR(1) model, $\boldsymbol{\theta} = \{\sigma^2, \rho\}$, the score (gradient of loglikelihood function) is

$$\begin{aligned}
\nabla_{\sigma^2}\mathcal{L} &= -\sum_{i=1}^{n} \frac{\frac{d_i}{2}}{1+\frac{d_i\sigma^2}{2}} + \sum_{i=1}^{n} \frac{\frac{1}{2}r_i(\boldsymbol{\beta})^\top \boldsymbol{V}_i(\rho) r_i(\boldsymbol{\beta})}{1+\frac{\sigma^2}{2}r_i(\boldsymbol{\beta})^\top \boldsymbol{V}_i(\rho) r_i(\boldsymbol{\beta})} \\
\nabla_{\rho}\mathcal{L} &= \sum_{i=1}^{n} \frac{1}{1+\frac{\sigma^2}{2}r_i(\boldsymbol{\beta})^\top \boldsymbol{V}_i(\rho) r_i(\boldsymbol{\beta})} * \frac{\sigma^2}{2} r_i(\boldsymbol{\beta})^\top \nabla \boldsymbol{V}_i(\rho) r_i(\boldsymbol{\beta}).
\end{aligned}$$

The approximate Hessian is

$$\begin{aligned}
d^2_{\sigma^2}\mathcal{L} &= \sum_{i=1}^{n} \frac{(\frac{d_i}{2})^2}{(1+\frac{d_i}{2}\sigma^2)^2} - \sum_{i=1}^{n} \frac{(\frac{1}{2}r_i(\boldsymbol{\beta})^\top \boldsymbol{V}_i(\rho) r_i(\boldsymbol{\beta}))^2}{(1+\frac{\sigma^2}{2}r_i(\boldsymbol{\beta})^\top \boldsymbol{V}_i(\rho) r_i(\boldsymbol{\beta}))^2} \\
d^2_{\rho}\mathcal{L} &= \sum_{i=1}^{n} \frac{1}{1+\frac{\sigma^2}{2}r_i(\boldsymbol{\beta})^\top \boldsymbol{V}_i(\rho) r_i(\boldsymbol{\beta})} * \frac{\sigma^2}{2} r_i(\boldsymbol{\beta})^\top d^2 \boldsymbol{V}_i(\rho) r_i(\boldsymbol{\beta}) \\
&- \sum_{i=1}^{n} \frac{1}{(1+\frac{\sigma^2}{2}r_i(\boldsymbol{\beta})^\top \boldsymbol{V}_i(\rho) r_i(\boldsymbol{\beta}))^2} * \Big(\frac{\sigma^2}{2} r_i(\boldsymbol{\beta})^\top \nabla \boldsymbol{V}_i(\rho) r_i(\boldsymbol{\beta})\Big)^2,
\end{aligned}$$

For the CS model, $\boldsymbol{\theta} = (\sigma^2, \rho)$, and the gradient is

$$\begin{aligned}
\nabla_{\sigma^2}\mathcal{L} &= -\sum_{i=1}^{n} \frac{\frac{d_i}{2}}{1+\frac{d_i\sigma^2}{2}} + \sum_{i=1}^{n} \frac{\frac{1}{2}r_i(\boldsymbol{\beta})^\top \boldsymbol{V}_i(\rho) r_i(\boldsymbol{\beta})}{1+\frac{\sigma^2}{2}r_i(\boldsymbol{\beta})^\top \boldsymbol{V}_i(\rho) r_i(\boldsymbol{\beta})} \\
\nabla_{\rho}\mathcal{L} &= \sum_{i=1}^{n} \frac{1}{1+\frac{\sigma^2}{2}r_i(\boldsymbol{\beta})^\top \boldsymbol{V}_i(\rho) r_i(\boldsymbol{\beta})} * \frac{\sigma^2}{2} r_i(\boldsymbol{\beta})^\top \nabla \boldsymbol{V}_i(\rho) r_i(\boldsymbol{\beta}).
\end{aligned}$$

The approximate Hessian is

$$\begin{aligned}
d^2_{\sigma^2}\mathcal{L} &= \sum_{i=1}^{n} \frac{(\frac{d_i}{2})^2}{(1+\frac{d_i}{2}\sigma^2)^2} - \sum_{i=1}^{n} \frac{(\frac{1}{2}r_i(\boldsymbol{\beta})^\top \boldsymbol{V}_i(\rho) r_i(\boldsymbol{\beta}))^2}{(1+\frac{\sigma^2}{2}r_i(\boldsymbol{\beta})^\top \boldsymbol{V}_i(\rho) r_i(\boldsymbol{\beta}))^2} \\
d^2_{\rho}\mathcal{L} &= -\sum_{i=1}^{n} \frac{1}{(1+\frac{\sigma^2}{2}r_i(\boldsymbol{\beta})^\top \boldsymbol{V}_i(\rho) r_i(\boldsymbol{\beta}))^2} * \Big(\frac{\sigma^2}{2} r_i(\boldsymbol{\beta})^\top \nabla \boldsymbol{V}_i(\rho) r_i(\boldsymbol{\beta})\Big)^2,
\end{aligned}$$

where $\nabla \boldsymbol{V}_i(\rho)$ and $d^2\boldsymbol{V}_i(\rho)$ are, respectively, the element-wise first and second derivatives of the matrix $\boldsymbol{V}_i(\rho)$ with respect to ρ.

For the VM model the gradient is

$$\nabla_{\boldsymbol{\theta}} f(\boldsymbol{\theta}) = \sum_{i=1}^{n} \frac{1}{(1+\boldsymbol{\theta}^\top \boldsymbol{b}_i)} * \boldsymbol{b}_i - \sum_{i=1}^{n} \frac{1}{1+\boldsymbol{\theta}^\top \boldsymbol{c}_i} * \boldsymbol{c}_i.$$

The approximate Hessian is

$$d^2_{\boldsymbol{\theta},\boldsymbol{\theta}} f(\boldsymbol{\theta}) = -\sum_{i=1}^{n} \frac{1}{(1+\boldsymbol{\theta}^\top \boldsymbol{b}_i)} * \boldsymbol{b}_i \boldsymbol{b}_i^\top + \sum_{i=1}^{n} \frac{1}{1+\boldsymbol{\theta}^\top \boldsymbol{c}_i} * \boldsymbol{c}_i \boldsymbol{c}_i^\top.$$

S1.3.4 Nuisance parameter estimation for Negative Binomial base

To estimate the nuisance parameter r in a Negative Binomial model, we use maximum likelihood. Because we are dealing with 1 parameter optimization, Newton's method is a good candidate due to its quadratic rate of convergence. The full loglikelihood is

$$-\sum_{i=1}^{n} \ln\left(1+\frac{1}{2}\operatorname{tr}(\boldsymbol{\Gamma}_i)\right) + \sum_{i=1}^{n}\sum_{i=j}^{d_i} \ln f_{ij}(y_{ij} \mid \boldsymbol{\beta}) + \sum_{i=1}^{n} \ln\left(1+\frac{1}{2}r_i(\boldsymbol{\beta})^\top \boldsymbol{\Gamma}_i r_i(\boldsymbol{\beta})\right)$$

where only the 2nd and 3rd term depends on r. First consider the 2nd term. Because $\mu_{ij} = \frac{r(1-p_{ij})}{p_{ij}}, p_{ij} = \frac{r}{r+\mu_{ij}}$, the 2nd term of the loglikelihood is

$$\begin{aligned}
&\sum_{i=1}^{n}\sum_{j=1}^{d_i} \ln\left[\binom{y_{ij}+r-1}{y_{ij}} p_{ij}^{r}(1-p_{ij})^{y_{ij}}\right] \\
=&\sum_{i=1}^{n}\sum_{j=1}^{d_i} \ln\binom{y_{ij}+r-1}{y_{ij}} + r\ln\left(\frac{r}{\mu_{ij}+r}\right) + y_{ij}\ln\left(\frac{\mu_{ij}}{\mu_{ij}+r}\right) \\
=&\sum_{i=1}^{n}\sum_{j=1}^{d_i} \ln((y_{ij}+r-1)!) - \ln(y_{ij}!) - \ln((r-1)!) + r\ln(r) - (r+y_{ij})\ln(\mu_{ij}+r) + y_{ij}\ln(\mu_{ij})
\end{aligned}$$

Let $\Psi^{(0)}$ be the digamma function and $\Psi^{(1)}$ the trigamma function, then the first and second derivative is

$$\begin{aligned}
&\sum_{i=1}^{n}\sum_{j=1}^{d_i} \Psi^{(0)}(y_{ij}+r) - \Psi^{(0)}(r) + 1 + \ln(r) - \frac{r+y_{ij}}{\mu_{ij}+r} - \ln(\mu_{ij}+r), \\
&\sum_{i=1}^{n}\sum_{j=1}^{d_i} \Psi^{(1)}(y_{ij}+r) - \Psi^{(0)}(r) + \frac{1}{r} - \frac{2}{\mu_{ij}+r} + \frac{r+y_{ij}}{(\mu_{ij}+r)^2}.
\end{aligned}$$

Now consider the 3rd term of the full loglikelihood. First recall

$$\begin{aligned}
\boldsymbol{D}_i &= diagonal(\sqrt{var(\boldsymbol{y}_i)}) \\
var(y_{ij}) &= \frac{r(1-p_{ij})}{p_{ij}^2} = \frac{e^{\eta_{ij}}(e^{\eta_{ij}}+r)}{r}.
\end{aligned}$$

Repeated application of the chain rule leads to

$$\begin{aligned}
\frac{d}{dr}\ln\left(1+\frac{1}{2}r_i(\boldsymbol{\beta})^\top\boldsymbol{\Gamma}_i r_i(\boldsymbol{\beta})\right) &= \sum_{i=1}^{n} \frac{r_i(\boldsymbol{\beta})^\top\boldsymbol{\Gamma}_i dr_i}{1+\frac{1}{2}r_i(\boldsymbol{\beta})^\top\boldsymbol{\Gamma}_i r_i(\boldsymbol{\beta})} \\
\frac{d^2}{dr^2}\ln\left(1+\frac{1}{2}r_i(\boldsymbol{\beta})^\top\boldsymbol{\Gamma}_i r_i(\boldsymbol{\beta})\right) &= \sum_{i=1}^{n} \frac{-[r_i(\boldsymbol{\beta})^\top\boldsymbol{\Gamma}_i dr_i]^2}{[1+\frac{1}{2}r_i(\boldsymbol{\beta})^\top\boldsymbol{\Gamma}_i r_i(\boldsymbol{\beta})]^2} + \frac{d\boldsymbol{r}(\boldsymbol{\beta})^\top\boldsymbol{\Gamma}_i dr_i(\boldsymbol{\beta}) + \boldsymbol{r}(\boldsymbol{\beta})^\top\boldsymbol{\Gamma}_i dr_i^2(\boldsymbol{\beta})}{1+\frac{1}{2}r_i(\boldsymbol{\beta})^\top\boldsymbol{\Gamma}_i r_i(\boldsymbol{\beta})}
\end{aligned}$$

where

$$
\begin{aligned}
r_i(\boldsymbol{\beta}) &= \boldsymbol{D}_i^{-1}(\boldsymbol{y}_i - \boldsymbol{\mu}_i)\\
dr_i(\boldsymbol{\beta}) &= -\boldsymbol{D}_i^{-1} d\boldsymbol{D}_i \boldsymbol{D}_i^{-1}(\boldsymbol{y}_i - \boldsymbol{\mu}_i)\\
dr_i^2(\boldsymbol{\beta}) &= [2\boldsymbol{D}_i^{-1} d\boldsymbol{D}_i \boldsymbol{D}_i^{-1} d\boldsymbol{D}_i \boldsymbol{D}_i^{-1} - \boldsymbol{D}_i^{-1} d^2\boldsymbol{D}_i \boldsymbol{D}_i^{-1}](\boldsymbol{y}_i - \boldsymbol{\mu}_i)\\
d\boldsymbol{D}_i &= diagonal\left(\frac{d}{dr}\sqrt{\frac{e^{\eta_{ij}}(e^{\eta_{ij}}+r)}{r}}\right) = diagonal\left(\frac{-e^{2\eta_{ij}}}{2r^{1.5}\sqrt{e^{\eta_{ij}}(e^{\eta_{ij}}+r)}}\right)\\
d^2\boldsymbol{D}_i &= diagonal\left(\frac{e^{3\eta}}{4r^{1.5}(e^{\eta}(e^{\eta}+r))^{1.5}} + \frac{3e^{2\eta}}{4r^{2.5}(e^{\eta}(e^{\eta}+r))^{0.5}}\right).
\end{aligned}
$$

Note we used the identity $df(\boldsymbol{X})^{-1} = -f(\boldsymbol{X})^{-1}df(\boldsymbol{X})f(\boldsymbol{X})^{-1}$ for obtaining $dr_i(\boldsymbol{\beta})$ and for obtaining $d\boldsymbol{r}_i^2(\boldsymbol{\beta})$, chain rule implies

$$
\begin{aligned}
& d(f(\boldsymbol{X})^{-1}df(\boldsymbol{X})f(\boldsymbol{X})^{-1})\\
=& [-f(\boldsymbol{X})^{-1}df(\boldsymbol{X})f(\boldsymbol{X})^{-1}]df(\boldsymbol{X})f(\boldsymbol{X})^{-1} + f(\boldsymbol{X})^{-1}d(df(\boldsymbol{X})f(\boldsymbol{X})^{-1})\\
=& -f(\boldsymbol{X})^{-1}df(\boldsymbol{X})f(\boldsymbol{X})^{-1}df(\boldsymbol{X})f(\boldsymbol{X})^{-1} +\\
& f(\boldsymbol{X})^{-1}\left[df(\boldsymbol{X})(-f(\boldsymbol{X})^{-1}df(\boldsymbol{X})f(\boldsymbol{X})^{-1}) + d^2f(\boldsymbol{X})f(\boldsymbol{X})^{-1}\right]\\
=& -2f(\boldsymbol{X})^{-1}df(\boldsymbol{X})f(\boldsymbol{X})^{-1}df(\boldsymbol{X})f(\boldsymbol{X})^{-1} + f(\boldsymbol{X})^{-1}d^2f(\boldsymbol{X})f(\boldsymbol{X})^{-1}
\end{aligned}
$$

In summary, we update the nuisance parameter r using Newton's update

$$
r_{n+1} = r_n - \frac{\frac{d}{dr}L(r \mid \boldsymbol{\mu}, \boldsymbol{\Gamma}, \boldsymbol{y})}{\frac{d^2}{dr^2}L(r \mid \boldsymbol{\mu}, \boldsymbol{\Gamma}, \boldsymbol{y})}
$$

where

$$
\begin{aligned}
\frac{d}{dr}L(r \mid \boldsymbol{\mu}, \boldsymbol{\Gamma}, \boldsymbol{y}) &= \sum_{i=1}^{n}\sum_{j=1}^{d_i} \Psi^{(0)}(y_{ij}+r) - \Psi^{(0)}(r) + 1 + \ln(r) - \frac{r+y_{ij}}{\mu_{ij}+r} - \ln(\mu_{ij}+r)\\
&\quad + \sum_{i=1}^{n} \frac{r_i(\boldsymbol{\beta})^\top \boldsymbol{\Gamma}_i dr_i}{1+\frac{1}{2}r_i(\boldsymbol{\beta})^\top \boldsymbol{\Gamma}_i r_i(\boldsymbol{\beta})}\\
\frac{d^2}{dr^2}L(r \mid \boldsymbol{\mu}, \boldsymbol{\Gamma}, \boldsymbol{y}) &= \sum_{i=1}^{n}\sum_{j=1}^{d_i} \Psi^{(1)}(y_{ij}+r) - \Psi^{(0)}(r) + \frac{1}{r} - \frac{2}{\mu_{ij}+r} + \frac{r+y_{ij}}{(\mu_{ij}+r)^2}\\
&\quad - \sum_{i=1}^{n} \frac{[r_i(\boldsymbol{\beta})^\top \boldsymbol{\Gamma}_i dr_i]^2}{[1+\frac{1}{2}r_i(\boldsymbol{\beta})^\top \boldsymbol{\Gamma}_i r_i(\boldsymbol{\beta})]^2} + \frac{dr_i(\boldsymbol{\beta})^\top \boldsymbol{\Gamma}_i dr_i(\boldsymbol{\beta}) + r_i(\boldsymbol{\beta})^\top \boldsymbol{\Gamma}_i dr_i^2(\boldsymbol{\beta})}{1+\frac{1}{2}r_i(\boldsymbol{\beta})^\top \boldsymbol{\Gamma}_i r_i(\boldsymbol{\beta})}
\end{aligned}
$$

For stability, we need to (1) perform line-search and (2) set the second derivative equal to 1 if it is negative. By default, we allow for a maximum of 10 block iterations; In each block iteration, we allow for a maximum of 15 iterations for the quasi-newton update of $\boldsymbol{\beta}$ and $\boldsymbol{\theta}$, and a maximum of 10 newton iterations for the update of r.

S1.3.5 Compound Symmetric $\boldsymbol{\Gamma}_i$

Under the Compound Symmetric (CS) parameterization of $\boldsymbol{\Gamma}_i$,

$$\begin{aligned}\boldsymbol{\Gamma}_i &= \sigma^2 \times \Big[\rho \mathbf{1}_{d_i}\mathbf{1}_{d_i}^\top + (1-\rho)\boldsymbol{I}_{\boldsymbol{d_i}}\Big] \\ &= \sigma^2 \times \boldsymbol{V}_i(\rho)\end{aligned}$$

Bounding Correlation Parameter To ensure that $\boldsymbol{\Gamma}_i$ is positive semi-definite, we will focus on $\boldsymbol{V}_i(\rho)$ and use an eigenvalue argument to bound $\rho \in (-\frac{1}{d_i-1}, 1)$. Let $\boldsymbol{v}$ be a vector of dimension d_i such that $<\boldsymbol{v}, \boldsymbol{v}>= 1$. We will find the conditions on ρ such that $\boldsymbol{v}^\top \boldsymbol{V}_i(\rho)\boldsymbol{v} \geq 0$.

$$\begin{aligned}\boldsymbol{v}^\top \boldsymbol{V}_i(\rho)\boldsymbol{v} &= \boldsymbol{v}^\top\Big[\rho \mathbf{1}_{d_i}\mathbf{1}_{d_i}^\top + (1-\rho)\boldsymbol{I}_{\boldsymbol{d_i}}\Big]\boldsymbol{v} \\ &= \rho \boldsymbol{v}^\top \mathbf{1}_{d_i}\mathbf{1}_{d_i}^\top \boldsymbol{v} + (1-\rho)\boldsymbol{v}^\top\boldsymbol{v} \\ &= \rho(\mathbf{1}_{d_i}^\top \boldsymbol{v})^2 + 1 - \rho \\ &= \rho\Big((\mathbf{1}_{d_i}^\top \boldsymbol{v})^2 - 1\Big) + 1 \\ &\geq 0\end{aligned}$$

Now solving for ρ and using the Cauchy-Schwartz Inequality, we get

$$\begin{aligned}\rho &\geq \frac{-1}{\Big((\mathbf{1}_{d_i}^\top \boldsymbol{v})^2 - 1\Big)} \\ &\geq \frac{-1}{(\mathbf{1}_{d_i}^\top \mathbf{1}_{d_i}) * (\boldsymbol{v}^\top \boldsymbol{v}) - 1} \\ &= \frac{-1}{d_i - 1}\end{aligned}$$

In summary, the CS longitudinal model has an additional constraint on the parameter space: $\rho \geq \frac{-1}{d_i-1}$.

S1.3.6 Gradients and Hessians of residual function

Here we give an example to calculate the explicit expressions for $\nabla r_{ij}(\boldsymbol{\beta})$ and $\nabla^2 r_{ij}(\boldsymbol{\beta})$. For simplicity, we consider the longitudinal Bernoulli model with logit link function. In the main text, we saw that the chain and product rules gives the following expressions

$$
\begin{aligned}
r_{ij}(\boldsymbol{\beta}) &= \frac{y_{ij} - \mu_{ij}}{\sqrt{\sigma_{ij}^2(\boldsymbol{\beta})}}, \quad (r_{ij}(\boldsymbol{\beta}) \in \mathbb{R} \text{ denotes sample } i\text{'s residual at time } j, \boldsymbol{r}_i(\boldsymbol{\beta}) \in \mathbb{R}^{d_i}) \\
\nabla r_{ij}(\boldsymbol{\beta}) &= -\frac{1}{\sqrt{\sigma_{ij}^2(\boldsymbol{\beta})}} \nabla \mu_{ij}(\boldsymbol{\beta}) - \frac{1}{2} \frac{y_{ij} - \mu_{ij}(\boldsymbol{\beta})}{\sigma_{ij}^3(\boldsymbol{\beta})} \nabla \sigma_{ij}^2(\boldsymbol{\beta}) \\
& \quad \big(\nabla r_{ij}(\boldsymbol{\beta}) \in \mathbb{R}^p \text{ is } j\text{th column of } \nabla \boldsymbol{r}_i(\boldsymbol{\beta}) \in \mathbb{R}^{p \times d_i}\big) \\
\nabla^2 r_{ij}(\boldsymbol{\beta}) &= \frac{-1}{\sqrt{\sigma_{ij}^2(\boldsymbol{\beta})}} \nabla^2 \mu_{ij}(\boldsymbol{\beta}) + \frac{1}{2} \frac{1}{\sigma_{ij}^3(\boldsymbol{\beta})} \nabla \sigma_{ij}^2(\boldsymbol{\beta}) \nabla \mu_{ij}(\boldsymbol{\beta})^\top - \left(\frac{1}{2} \frac{y_{ij} - \mu_{ij}(\boldsymbol{\beta})}{\sigma_{ij}^3(\boldsymbol{\beta})} \nabla^2 \sigma_{ij}^2(\boldsymbol{\beta}) \right) \\
& \quad - \nabla \left\{ \frac{1}{2} \frac{y_{ij} - \mu_{ij}(\boldsymbol{\beta})}{(\sigma_{ij}^2(\boldsymbol{\beta}))^{3/2}} \right\} \nabla \sigma_{ij}^2(\boldsymbol{\beta})^\top \\
&= \frac{-1}{\sqrt{\sigma_{ij}^2(\boldsymbol{\beta})}} \nabla^2 \mu_{ij}(\boldsymbol{\beta}) + \frac{1}{2} \frac{1}{\sigma_{ij}^3(\boldsymbol{\beta})} \nabla \sigma_{ij}^2(\boldsymbol{\beta}) \nabla \mu_{ij}(\boldsymbol{\beta})^\top - \left(\frac{1}{2} \frac{y_{ij} - \mu_{ij}(\boldsymbol{\beta})}{\sigma_{ij}^3(\boldsymbol{\beta})} \nabla^2 \sigma_{ij}^2(\boldsymbol{\beta}) \right) \\
& \quad - \frac{1}{2} \left\{ \frac{-1}{(\sigma_{ij}^2(\boldsymbol{\beta}))^{3/2}} \nabla \mu_{ij}(\boldsymbol{\beta}) - \frac{3}{2} \frac{y_{ij} - \mu_{ij}(\boldsymbol{\beta})}{(\sigma_{ij}^2(\boldsymbol{\beta}))^{5/2}} \nabla \sigma_{ij}^2(\boldsymbol{\beta}) \right\} \nabla \sigma_{ij}^2(\boldsymbol{\beta})^\top
\end{aligned}
$$

To calculate these quantities, we need expressions for $\nabla \mu_{ij}(\boldsymbol{\beta}) \in \mathbb{R}^p, \nabla^2 \mu_{ij}(\boldsymbol{\beta}) \in \mathbb{R}^{p \times p}, \nabla \sigma_{ij}^2(\boldsymbol{\beta}) \in \mathbb{R}^p$, and $\nabla^2 \sigma_{ij}^2(\boldsymbol{\beta}) \in \mathbb{R}^{p \times p}$ (note these gradients are evaluated with respect to $\boldsymbol{\beta}$). Since $\boldsymbol{\mu}_i = \boldsymbol{g}^{[-1]}(\boldsymbol{\eta}_i) = \boldsymbol{g}^{[-1]}(\boldsymbol{X}_i \boldsymbol{\beta})$, by chain rule

$$
\begin{aligned}
\nabla \mu_{ij}(\boldsymbol{\beta}) &= \frac{\partial \mu_{ij}}{\partial \eta_{ij}} \frac{\partial \eta_{ij}}{\partial \boldsymbol{\beta}} = \frac{\partial \mu_{ij}}{\partial \eta_{ij}} \boldsymbol{x}_{ij} \quad (\boldsymbol{x}_{ij} \in \mathbb{R}^p \text{ are covariates for sample } i \text{ at time } j) \\
\nabla^2 \mu_{ij}(\boldsymbol{\beta}) &= \frac{\partial}{\partial \boldsymbol{\beta}} \left(\frac{\partial \mu_{ij}}{\partial \eta_{ij}} \boldsymbol{x}_{ij} \right) = \frac{\partial^2 \mu_{ij}^2}{\partial \eta_{ij}^2} \frac{\partial \eta_{ij}}{\partial \boldsymbol{\beta}} \boldsymbol{x}_{ij} = \frac{\partial^2 \mu_{ij}^2}{\partial \eta_{ij}^2} \boldsymbol{x}_{ij} \boldsymbol{x}_{ij}^\top \in \mathbb{R}^{p \times p}
\end{aligned}
$$

Here $\frac{\partial \mu_{ij}}{\partial \eta_{ij}} = \frac{\partial [g^{[-1]}(\boldsymbol{x}_{ij}^\top \boldsymbol{\beta})]}{\partial [\boldsymbol{x}_{ij}^\top \boldsymbol{\beta}]} \in \mathbb{R}$ is just the derivative of the inverse link function evaluated at the linear predictor $\eta_{ij} = \boldsymbol{x}_{ij}^\top \boldsymbol{\beta} \in \mathbb{R}$. This is implemented for various link functions as mueta in GLM.jl which we call internally. Following its definition, we also implement `mueta2` for evaluating $\frac{\partial^2 \mu_{ij}}{\partial \eta_{ij}^2} \in \mathbb{R}$.

To compute $\nabla\sigma_{ij}^2(\boldsymbol{\beta})$ and $\nabla^2\sigma_{ij}^2(\boldsymbol{\beta})$, note variance is typically a function of the mean, that is,

$$\begin{aligned}
\nabla\sigma_{ij}^2(\boldsymbol{\beta}) &= \frac{\partial\sigma_{ij}^2}{\partial\mu_{ij}}\frac{\partial\mu_{ij}}{\partial\eta_{ij}}\frac{\partial\eta_{ij}}{\partial\boldsymbol{\beta}} = \frac{\partial\sigma_{ij}^2}{\partial\mu_{ij}}\frac{\partial\mu_{ij}}{\partial\eta_{ij}}\boldsymbol{x}_{ij} \in \mathbb{R}^p \\
\nabla^2\sigma_{ij}^2(\boldsymbol{\beta}) &= \frac{\partial^2\sigma_{ij}^2}{\partial\mu_{ij}^2}\frac{\partial\mu_{ij}}{\partial\eta_{ij}}\frac{\partial\eta_{ij}}{\partial\boldsymbol{\beta}}\frac{\partial\mu_{ij}}{\partial\eta_{ij}}\boldsymbol{x}_{ij} + \frac{\partial\sigma_{ij}^2}{\partial\mu_{ij}}\frac{\partial^2\mu_{ij}}{\partial\eta_{ij}^2}\frac{\partial\eta_{ij}}{\partial\boldsymbol{\beta}}\boldsymbol{x}_{ij} \\
&= \left(\frac{\partial^2\sigma_{ij}^2}{\partial\mu_{ij}^2}\left(\frac{\partial\mu_{ij}}{\partial\eta_{ij}}\right)^2 + \frac{\partial\sigma_{ij}^2}{\partial\mu_{ij}}\frac{\partial^2\mu_{ij}}{\partial\eta_{ij}^2}\right)\boldsymbol{x}_{ij}\boldsymbol{x}_{ij}^\top \in \mathbb{R}^{p\times p}
\end{aligned}$$

Terms $\frac{\partial\sigma_{ij}^2}{\partial\mu_{ij}}$ and $\frac{\partial^2\sigma_{ij}^2}{\partial\mu_{ij}^2}$ are distribution-dependent, and we implement them as `sigmamu` and `sigmamu2` in our software.

For Bernoulli distribution, if μ is the mean, then $\mu(1-\mu)$ is its variance. Given the logit link, we have

$$\begin{aligned}
\nabla\mu_{ij} &= \frac{\partial\mu_{ij}}{\partial\eta_{ij}}\frac{\partial\eta_{ij}}{\partial\boldsymbol{\beta}} = \frac{e^{-\eta_{ij}}}{(1+e^{-\eta_{ij}})^2}\boldsymbol{x}_{ij} \quad \left(\text{expression for } \frac{\partial\mu_{ij}}{\partial\eta_{ij}} \text{ uses the logit link}\right) \\
\nabla\sigma_{ij}^2 &= \frac{\partial\sigma_{ij}^2}{\partial\mu_{ij}}\frac{\partial\mu_{ij}}{\partial\eta_{ij}}\frac{\partial\eta_{ij}}{\partial\boldsymbol{\beta}} = \frac{\partial(\mu_{ij}(1-\mu_{ij}))}{\partial\mu_{ij}}\frac{e^{-\eta_{ij}}}{(1+e^{-\eta_{ij}})^2}\boldsymbol{x}_{ij} = (1-2\mu_{ij})\frac{e^{-\eta_{ij}}}{(1+e^{-\eta_{ij}})^2}\boldsymbol{x}_{ij}.
\end{aligned}$$

Differentiating again yields the expressions

$$\begin{aligned}
\nabla^2\mu_{ij}^2 &= \left(\frac{-e^{-\eta_{ij}}}{(1+e^{-\eta_{ij}})^2} + \frac{2e^{-2\eta_{ij}}}{(1+e^{-\eta_{ij}})^3}\right)\boldsymbol{x}_{ij}\boldsymbol{x}_{ij}^\top \\
\nabla^2\sigma_{ij}^2 &= -2\boldsymbol{x}_{ij}\boldsymbol{x}_{ij}^\top.
\end{aligned}$$

For the special Gaussian base, the variance is parametrized by $\tau = \sigma^{-2}$ (see section S1.4) which is a separate parameter from the mean. Thus, $\frac{\partial\sigma^2}{\partial\mu} = 0$. Given the identity link $\boldsymbol{\mu}_i(\boldsymbol{\beta}) = \boldsymbol{\eta}_i = \boldsymbol{X}_i\boldsymbol{\beta}$,

$$[\nabla\boldsymbol{r}_i(\boldsymbol{\beta})]_j = \frac{-1}{\sigma}\boldsymbol{x}_{ij} = -\sqrt{\tau}\boldsymbol{x}_{ij}$$

S1.4 Special case: Gaussian approximate-copulas

This section considers the special case of Gaussian base in the approximate-copula framework, and presents detailed derivations of data generation and estimation methods. The joint density of $\mathbf{y} \in \mathbb{R}^d$ is

$$\left(c + \frac{1}{2}\mathrm{tr}\boldsymbol{\Gamma}\right)^{-1}\left(\frac{1}{\sqrt{2\pi}\sigma_0}\right)^d e^{-\frac{\|\mathbf{y}-\boldsymbol{\mu}\|_2^2}{2\sigma_0^2}}\left[c + \frac{1}{2\sigma_0^2}(\mathbf{y}-\boldsymbol{\mu})^\top\boldsymbol{\Gamma}(\mathbf{y}-\boldsymbol{\mu})\right]. \tag{9}$$

The parameter $c \geq 0$ tips the balance between the independent and dependent components.

S1.4.1 Moments

In the Gaussian case, we have

$$\begin{aligned}
\mathbb{E}(y_i) &= \mu_i \\
\mathbf{Var}(y_i) &= \sigma_0^2 \left(1 + \frac{\gamma_{ii}}{c + \frac{1}{2}\text{tr}(\boldsymbol{\Gamma})}\right) \\
\mathbf{Cov}(y_i, y_j) &= \sigma_0^2 \frac{\gamma_{ij}}{c + \frac{1}{2}\text{tr}(\boldsymbol{\Gamma})}, \\
\mathbf{Cor}(y_i, y_j) &= \frac{\gamma_{ij}}{\sqrt{(c + \frac{1}{2}\text{tr}(\boldsymbol{\Gamma}) + \gamma_{ii})(c + \frac{1}{2}\text{tr}(\boldsymbol{\Gamma}) + \gamma_{jj})}}.
\end{aligned}$$

In summary,

$$\mathbf{Cov}(\mathbf{y}) = \sigma_0^2 \left[\mathbf{I} + \left(\frac{1}{c + \frac{1}{2}\text{tr}\boldsymbol{\Gamma}}\right)\boldsymbol{\Gamma}\right].$$

In the special case of $\boldsymbol{\Gamma} = \sigma_1^2 \mathbf{I}$, we have

$$\begin{aligned}
\mathbf{Var}(y_i) &= \sigma_0^2 \left(1 + \frac{\sigma_1^2}{c + \frac{n}{2}\sigma_1^2}\right)\mathbf{I} \\
\mathbf{Cov}(y_i, y_j) &= 0, \quad i \neq j.
\end{aligned}$$

In the regression model, we would keep the variance σ_0^2 parameter for more flexibility in modeling the variance.

Random number generation If we are able to generate a residual vector $\mathbf{R}$ from the (standardized) Gaussian copula model

$$\left[1 + \frac{1}{2}\text{tr}(\boldsymbol{\Gamma})\right]^{-1} \left(\frac{1}{\sqrt{2\pi}}\right)^d e^{-\frac{\|\mathbf{r}\|_2^2}{2}} \left(1 + \frac{1}{2}\mathbf{r}^\top \boldsymbol{\Gamma} \mathbf{r}\right),$$

then $\mathbf{Y} = \sigma_0 \mathbf{R} + \boldsymbol{\mu}$ is a desired sample from density (9).

To generate a sample from the standardized Gaussian copula model, we first sample R_1 from its marginal distribution and then generate remaining components sequentially from the conditional distributions $R_k \mid R_1, \ldots, R_{k-1}$ for $k = 2, \ldots, d$.

- To generate R_1 from its marginal density

$$\left[1+\frac{1}{2}\mathrm{tr}(\mathbf{\Gamma})\right]^{-1}\frac{1}{\sqrt{2\pi}}e^{-\frac{r_1^2}{2}}\left[1+\frac{\gamma_{11}}{2}r_1^2+\frac{1}{2}\sum_{i=2}^{d}\gamma_{ii}\right], \tag{10}$$

  we recognize it as a mixture of three distributions Normal$(0,1)$, $\sqrt{\chi_3^2}$ and $-\sqrt{\chi_3^2}$ with mixing probabilities $\frac{1+0.5\sum_{i=2}^{d}\gamma_{ii}}{1+0.5\sum_{i=1}^{d}\gamma_{ii}}$, $\frac{0.25\gamma_{11}}{1+0.5\sum_{i=1}^{d}\gamma_{ii}}$ and $\frac{0.25\gamma_{11}}{1+0.5\sum_{i=1}^{d}\gamma_{ii}}$ respectively.

- Next we consider generating R_2 from the conditional distribution $R_2 \mid R_1$. Dividing the marginal distribution of (R_1, R_2)

$$\left[1+\frac{1}{2}\mathrm{tr}(\mathbf{\Gamma})\right]^{-1}\left(\frac{1}{\sqrt{2\pi}}\right)^2 e^{-\frac{r_1^2+r_2^2}{2}}\left(1+\frac{\gamma_{22}}{2}r_2^2+\gamma_{12}r_1r_2+\frac{\gamma_{11}}{2}r_1^2+\frac{1}{2}\sum_{i=3}^{d}\gamma_{ii}\right)$$

  by the marginal distribution of R_1 (10) yields the conditional density

$$\frac{\frac{1}{\sqrt{2\pi}}e^{-\frac{r_2^2}{2}}\left(1+\frac{\gamma_{11}}{2}r_1^2+\frac{1}{2}\sum_{i=3}^{d}\gamma_{ii}+\gamma_{12}r_1r_2+\frac{\gamma_{22}}{2}r_2^2\right)}{1+\frac{\gamma_{11}}{2}r_1^2+\frac{1}{2}\sum_{i=2}^{d}\gamma_{ii}},$$

  which unfortunately is not a mixture of standard distributions. However we can evaluate its cumulative distribution function (CDF)

$$F(x)=\frac{\left(1+\frac{\gamma_{11}}{2}r_1^2+\frac{1}{2}\sum_{i=3}^{d}\gamma_{ii}\right)\Phi(x)-\gamma_{12}r_1\phi(x)+\frac{\gamma_{22}}{2}\left[\frac{1}{2}+\frac{\mathrm{sgn}(x)}{2}F_{\chi_3^2}(x^2)\right]}{1+\frac{\gamma_{11}}{2}r_1^2+\frac{1}{2}\sum_{i=2}^{d}\gamma_{ii}}$$

  in terms of the density ϕ and CDF Φ of standard normal and the CDF $F_{\chi_3^2}$ of chi-squared distribution with degree of freedom 3. This suggests the inverse CDF approach. To generate one sample from $R_2 \mid R_1$, we draw a uniform variate U and use nonlinear root finding to locate R_2 such that $F(R_2) = U$.

- In general, the conditional distribution $R_k \mid R_1, \ldots, R_{k-1}$ has density

$$\frac{\frac{1}{\sqrt{2\pi}}e^{-\frac{r_k^2}{2}}\left(1+\frac{1}{2}\mathbf{r}_{[k-1]}^\top\mathbf{\Gamma}_{[k-1],[k-1]}\mathbf{r}_{[k-1]}+\frac{1}{2}\sum_{i=k+1}^{n}\gamma_{ii}+(\sum_{i=1}^{k-1}r_i\gamma_{ik})r_k+\frac{\gamma_{kk}}{2}r_k^2\right)}{1+\frac{1}{2}\mathbf{r}_{[k-1]}^\top\mathbf{\Gamma}_{[k-1],[k-1]}\mathbf{r}_{[k-1]}+\frac{1}{2}\sum_{i=k}^{d}\gamma_{ii}}$$

  and CDF

$$\frac{\left(1+\frac{1}{2}\mathbf{r}_{[k-1]}^\top\mathbf{\Gamma}_{[k-1],[k-1]}\mathbf{r}_{[k-1]}+\frac{1}{2}\sum_{i=k+1}^{d}\gamma_{ii}\right)\Phi(x)-(\sum_{i=1}^{k-1}r_i\gamma_{ik})\phi(x)+\frac{\gamma_{kk}}{2}\left[\frac{1}{2}+\frac{\mathrm{sgn}(x)}{2}F_{\chi_3^2}(x^2)\right]}{1+\frac{1}{2}\mathbf{r}_{[k-1]}^\top\mathbf{\Gamma}_{[k-1],[k-1]}\mathbf{r}_{[k-1]}+\frac{1}{2}\sum_{i=k}^{d}\gamma_{ii}}.$$

  We apply the inverse CDF approach to sample R_k given $R_1, \ldots, R_{k-1}$.

For a general GLM model, we need to sample from conditional densities of form $cf(y)(a_0 + a_1 y + a_2 y^2)$ where a_i, $i = 1, 2, 3$, are constants and c is a normalizing constant. For most continuous distributions, e.g., exponential, gamma, beta, chi-squared, and beta, the CDF can be expressed conveniently using special functions.

S1.4.2 Parameter Estimation

Suppose we have n independent realizations $\boldsymbol{y}_i$ from the approximate-copula density. Each of these may be of different dimensions, d_i. Assuming the component distribution $\mathbf{y}_i \sim \text{Normal}(\mathbf{X}_i\boldsymbol{\beta}, \sigma_0^2 \mathbf{I}_{d_i})$, the component densities take form

$$\ln f_i(\mathbf{y}_i \mid \boldsymbol{\beta}, \sigma_0^2) = -\frac{d_i}{2} \ln 2\pi - \frac{d_i}{2} \ln \sigma_0^2 - \frac{1}{2} \frac{\|\mathbf{y}_i - \mathbf{X}_i\boldsymbol{\beta}\|_2^2}{\sigma_0^2}$$

and the joint loglikelihood of the sample is

$$\begin{aligned}
& -\sum_i \ln\left(c + \frac{1}{2}\text{tr}(\boldsymbol{\Gamma}_i)\right) - \frac{\sum_i d_i}{2} \ln 2\pi - \frac{\sum_i d_i}{2} \ln \sigma_0^2 - \frac{1}{2} \frac{\sum_i \|\mathbf{y}_i - \mathbf{X}_i\boldsymbol{\beta}\|_2^2}{\sigma_0^2} \\
& + \sum_i \ln\left[c + \frac{1}{2\sigma_0^2}(\mathbf{y}_i - \mathbf{X}_i\boldsymbol{\beta})^\top \boldsymbol{\Gamma}_i (\mathbf{y}_i - \mathbf{X}_i\boldsymbol{\beta})\right] \\
= \; & -\sum_i \ln\left(c + \frac{1}{2}\text{tr}(\boldsymbol{\Gamma}_i)\right) - \frac{\sum_i d_i}{2} \ln 2\pi + \frac{\sum_i d_i}{2} \ln \tau - \frac{\tau}{2} \sum_i \|\mathbf{y}_i - \mathbf{X}_i\boldsymbol{\beta}\|_2^2 \\
& + \sum_i \ln\left[c + \frac{\tau}{2}(\mathbf{y}_i - \mathbf{X}_i\boldsymbol{\beta})^\top \boldsymbol{\Gamma}_i (\mathbf{y}_i - \mathbf{X}_i\boldsymbol{\beta})\right]
\end{aligned}$$

where $\boldsymbol{\Gamma}_i = \sum_{k=1}^m \theta_k \mathbf{V}_{ik}$ are parameterized via variance components $\boldsymbol{\theta} = (\theta_1, \ldots, \theta_m)$. We work with the parameterization $\tau = \sigma_0^{-2}$ because the loglikelihood is concave in τ.

Score and Hessian The score (gradient of loglikelihood function) is

$$
\begin{aligned}
\nabla_{\boldsymbol{\beta}} &= \sigma_0^{-2} \sum_i \mathbf{X}_i^\top (\mathbf{y}_i - \mathbf{X}_i \boldsymbol{\beta}) - \sum_i \frac{\mathbf{X}_i^\top \boldsymbol{\Gamma}_i (\mathbf{y}_i - \mathbf{X}_i \boldsymbol{\beta})}{c\sigma_0^2 + \frac{1}{2}(\mathbf{y}_i - \mathbf{X}_i \boldsymbol{\beta})^\top \boldsymbol{\Gamma}_i (\mathbf{y}_i - \mathbf{X}_i \boldsymbol{\beta})} \\
&= \tau \sum_i \mathbf{X}_i^\top (\mathbf{y}_i - \mathbf{X}_i \boldsymbol{\beta}) - \tau \sum_i \frac{\mathbf{X}_i^\top \boldsymbol{\Gamma}_i (\mathbf{y}_i - \mathbf{X}_i \boldsymbol{\beta})}{c + \frac{\tau}{2}(\mathbf{y}_i - \mathbf{X}_i \boldsymbol{\beta})^\top \boldsymbol{\Gamma}_i (\mathbf{y}_i - \mathbf{X}_i \boldsymbol{\beta})} \\
\nabla_\tau &= \frac{\sum_i d_i}{2\tau} - \frac{1}{2} \sum_i \|\mathbf{y}_i - \mathbf{X}_i \boldsymbol{\beta}\|_2^2 + \sum_i \frac{\frac{1}{2}(\mathbf{y}_i - \mathbf{X}_i \boldsymbol{\beta})^\top \boldsymbol{\Gamma}_i (\mathbf{y}_i - \mathbf{X}_i \boldsymbol{\beta})}{c + \frac{\tau}{2}(\mathbf{y}_i - \mathbf{X}_i \boldsymbol{\beta})^\top \boldsymbol{\Gamma}_i (\mathbf{y}_i - \mathbf{X}_i \boldsymbol{\beta})} \\
\nabla_c &= \sum_i \frac{1}{c + \frac{\tau}{2}(\mathbf{y}_i - \mathbf{X}_i \boldsymbol{\beta})^\top \boldsymbol{\Gamma}_i (\mathbf{y}_i - \mathbf{X}_i \boldsymbol{\beta})} \\
\nabla_{\boldsymbol{\theta}} &= -\sum_i \left(c + \sum_k \theta_k t_{ik} \right)^{-1} \mathbf{t}_i + \tau \sum_i \left(c + \tau \sum_k \theta_k q_{ik} \right)^{-1} \mathbf{q}_i
\end{aligned}
$$

where

$$
\begin{aligned}
t_{ik} &= \frac{1}{2} \mathrm{tr}(\boldsymbol{\Omega}_{ik}), \quad \mathbf{t}_i = (t_{i1}, \ldots, t_{im})^\top \\
q_{ik} &= \frac{1}{2} (\mathbf{y}_i - \mathbf{X}_i \boldsymbol{\beta})^\top \boldsymbol{\Omega}_{ik} (\mathbf{y}_i - \mathbf{X}_i \boldsymbol{\beta}), \quad \mathbf{q}_i = (q_{i1}, \ldots, q_{im})^\top.
\end{aligned}
$$

The Hessian is

$$
\begin{aligned}
d^2_{\boldsymbol{\beta},\boldsymbol{\beta}} &= -\tau \sum_i \mathbf{X}_i^\top \mathbf{X}_i + \sum_i \frac{\tau \mathbf{X}_i^\top \boldsymbol{\Gamma}_i \mathbf{X}_i}{c + \frac{\tau}{2}(\mathbf{y}_i - \mathbf{X}_i\boldsymbol{\beta})^\top \boldsymbol{\Gamma}_i (\mathbf{y}_i - \mathbf{X}_i\boldsymbol{\beta})} \\
&\quad - \sum_i \frac{\tau^2 [\mathbf{X}_i^\top \boldsymbol{\Gamma}_i (\mathbf{y}_i - \mathbf{X}_i\boldsymbol{\beta})][\mathbf{X}_i^\top \boldsymbol{\Gamma}_i (\mathbf{y}_i - \mathbf{X}_i\boldsymbol{\beta})]^\top}{\left[c + \frac{\tau}{2}(\mathbf{y}_i - \mathbf{X}_i\boldsymbol{\beta})^\top \boldsymbol{\Gamma}_i (\mathbf{y}_i - \mathbf{X}_i\boldsymbol{\beta})\right]^2} \\
&\approx -\tau \sum_i \mathbf{X}_i^\top \mathbf{X}_i - \tau \sum_i \frac{\tau [\mathbf{X}_i^\top \boldsymbol{\Gamma}_i (\mathbf{y}_i - \mathbf{X}_i\boldsymbol{\beta})][\mathbf{X}_i^\top \boldsymbol{\Gamma}_i (\mathbf{y}_i - \mathbf{X}_i\boldsymbol{\beta})]^\top}{\left[c + \frac{\tau}{2}(\mathbf{y}_i - \mathbf{X}_i\boldsymbol{\beta})^\top \boldsymbol{\Gamma}_i (\mathbf{y}_i - \mathbf{X}_i\boldsymbol{\beta})\right]^2} \\
d^2_{\boldsymbol{\beta},\tau} &= \sum_i \mathbf{X}_i^\top (\mathbf{y}_i - \mathbf{X}_i\boldsymbol{\beta}) - \sum_i \frac{\mathbf{X}_i^\top \boldsymbol{\Gamma}_i (\mathbf{y}_i - \mathbf{X}_i\boldsymbol{\beta})}{\left[c + \frac{\tau}{2}(\mathbf{y}_i - \mathbf{X}_i\boldsymbol{\beta})^\top \boldsymbol{\Gamma}_i (\mathbf{y}_i - \mathbf{X}_i\boldsymbol{\beta})\right]^2} \\
d^2_{\boldsymbol{\beta},\boldsymbol{\theta}} &= \sum_i \frac{\mathbf{X}_i^\top \boldsymbol{\Gamma}_i (\mathbf{y}_i - \mathbf{X}_i\boldsymbol{\beta}) \mathbf{q}_i^\top}{\left[c + \frac{\tau}{2}(\mathbf{y}_i - \mathbf{X}_i\boldsymbol{\beta})^\top \boldsymbol{\Gamma}_i (\mathbf{y}_i - \mathbf{X}_i\boldsymbol{\beta})\right]^2} \\
d^2_{\tau,\tau} &= -\frac{\sum_i d_i}{2\tau^2} - \sum_i \left[\frac{\frac{1}{2}(\mathbf{y}_i - \mathbf{X}_i\boldsymbol{\beta})^\top \boldsymbol{\Gamma}_i (\mathbf{y}_i - \mathbf{X}_i\boldsymbol{\beta})}{c + \frac{\tau}{2}(\mathbf{y}_i - \mathbf{X}_i\boldsymbol{\beta})^\top \boldsymbol{\Gamma}_i (\mathbf{y}_i - \mathbf{X}_i\boldsymbol{\beta})}\right]^2 \\
d^2_{\tau,\boldsymbol{\theta}} &= -\sum_i \frac{\mathbf{X}_i^\top \boldsymbol{\Gamma}_i (\mathbf{y}_i - \mathbf{X}_i\boldsymbol{\beta})}{\left[c + \frac{\tau}{2}(\mathbf{y}_i - \mathbf{X}_i\boldsymbol{\beta})^\top \boldsymbol{\Gamma}_i (\mathbf{y}_i - \mathbf{X}_i\boldsymbol{\beta})\right]^2} \mathbf{q}_i^\top \\
d^2_{\boldsymbol{\theta},\boldsymbol{\theta}} &= \sum_i \left(c + \sum_k \theta_k t_{ik}\right)^{-2} \mathbf{t}_i \mathbf{t}_i^\top - \tau \sum_i \left(c + \sum_k \theta_k q_{ik}\right)^{-2} \mathbf{q}_i \mathbf{q}_i^\top.
\end{aligned}
$$

Note $\mathbb{E}[d^2_{\boldsymbol{\beta},\tau}]$, $\mathbb{E}[d^2_{\boldsymbol{\beta},\boldsymbol{\theta}}]$, and $\mathbb{E}[d^2_{\tau,\boldsymbol{\theta}}]$ are approximately zero.

MM algorithm Because the MM update of $\boldsymbol{\theta}$ and τ is cheap, we maximize the profiled likelihood. That is, after each Newton update of $\boldsymbol{\beta}$, we update $(\tau, \boldsymbol{\theta})$ conditional on current $\boldsymbol{\beta}$ using the MM algorithm and evaluate the gradient and (approximate) Hessian using the newest $(\tau, \boldsymbol{\theta})$. To update τ and $\boldsymbol{\theta}$ given $\boldsymbol{\beta}$, the relevant objective function is

$$
-\sum_i \ln\left(c + \sum_k \theta_k t_{ik}\right) + \frac{\sum_i d_i}{2} \ln \tau - \frac{\sum_i r_i^2}{2}\tau + \sum_i \ln\left(c + \tau \sum_k \theta_k q_{ik}\right),
$$

which is minorized by

$$\begin{aligned}
- & \sum_i \sum_k \frac{t_{ik}}{c^{(t)} + \sum_k \theta_k^{(t)} t_{ik}} \theta_k - \sum_i \frac{1}{c^{(t)} + \sum_k \theta_k^{(t)} t_{ik}} c \\
+ & \frac{\sum_i d_i}{2} \ln \tau - \frac{\sum_i r_i^2}{2} \tau \\
+ & \sum_i \sum_k \frac{\tau^{(t)} \theta_k^{(t)} q_{ik}}{c^{(t)} + \tau^{(t)} \sum_k \theta_k^{(t)} q_{ik}} (\ln \tau + \ln \theta_k) \\
+ & \sum_i \frac{c^{(t)}}{c^{(t)} + \tau^{(t)} \sum_k \theta_k^{(t)} q_{ik}} \ln c \\
+ & \text{const.}
\end{aligned}$$

The resultant updates are

$$\begin{aligned}
\tau^{(t+1)} &= \frac{\sum_i d_i + 2 \sum_i \frac{\tau^{(t)} q_i^{(t)}}{c^{(t)} + \tau^{(t)} q_i^{(t)}}}{\sum_i r_i^2} \\
c^{(t+1)} &= c^{(t)} \frac{\sum_i \frac{1}{c^{(t)} + \tau^{(t)} q_i^{(t)}}}{\sum_i \frac{1}{c^{(t)} + t^{(t)}}} \qquad (11) \\
\theta_k^{(t+1)} &= \theta_k^{(t)} \frac{\sum_i \frac{\tau^{(t)} q_{ik}}{c^{(t)} + \tau^{(t)} q_i^{(t)}}}{\sum_i \frac{t_{ik}}{c^{(t)} + t_i^{(t)}}}, \quad k = 1, \ldots, m, \qquad (12)
\end{aligned}$$

where $q_i^{(t)} = \sum_k \theta_k^{(t)} q_{ik}$ and $t_i^{(t)} = \sum_k \theta_k^{(t)} t_{ik}$.

If we opt to use the optimal quadratic minorization

$$\ln(1 + x) \geq \ln(1 + x^{(t)}) + (x - x^{(t)}) - \frac{x^2 - x^{2(t)}}{2(1 + x^{(t)})},$$

the minorization function becomes

$$-\sum_i \sum_k \frac{t_{ik}}{1 + \sum_k \theta_k^{(t)} t_{ik}} \theta_k + \frac{\sum_i d_i}{2} \ln \tau + \left(\sum_i \sum_k \theta_k q_{ik} - \frac{\sum_i r_i^2}{2} \right) \tau - \frac{\tau^2}{2} \sum_i \frac{(\sum_k \theta_k q_{ik})^2}{1 + \tau^{(t)} \sum_k \theta_k^{(t)} q_{ik}} + c^{(t)}.$$

To update τ given θ_k, let

$$\begin{aligned} a^{(t)} &= \sum_i \frac{(\sum_k \theta_k^{(t)} q_{ik})^2}{1+\tau^{(t)}\sum_k \theta_k^{(t)} q_{ik}} \\ b^{(t)} &= \sum_i \sum_k \theta_k^{(t)} q_{ik} - \frac{\sum_i r_i^2}{2} \\ c^{(t)} &= \frac{\sum_i d_i}{2} \end{aligned}$$

then

$$\tau^{(t+1)} = \frac{b^{(t)} + \sqrt{b^{2(t)} + 4a^{(t)}c^{(t)}}}{2a^{(t)}}.$$

To update θ_k given τ, we minimize quadratic function

$$\frac{1}{2}\boldsymbol{\sigma}^{2T}\mathbf{Q}^\top\mathbf{W}^{(\mathbf{t})}\mathbf{Q}\boldsymbol{\sigma}^2 - \mathbf{c}^{(t)T}\boldsymbol{\sigma}^2$$

subject to nonnegativity constraint $\theta_k \geq 0$, where $\mathbf{W}^{(t)} = \text{diag}(w_1^{(t)}, \ldots, w_n^{(t)})$ with

$$w_i^{(t)} = \frac{\tau^{2(t)}}{1+\tau^{(t)}\sum_k \theta_k^{(t)} q_{ik}}$$

and $\mathbf{c}^{(t)}$ has entries

$$c_k^{(t)} = \tau^{(t)}\sum_i q_{ik} - \sum_i \frac{t_{ik}}{1+\sum_k \theta_k^{(t)} t_{ik}}.$$

It turns out this update based on quadratic minorization converges slower than the update (12) based on Jensen's inequality.

S1.5 Additional Simulations for longitudinal model

In each simulation scenario, the non-intercept entries of the predictor matrix $\boldsymbol{X}_i$ are independent standard normal deviates. When simulating under our model for the CS and AR(1) models, the true regression coefficients $\boldsymbol{\beta}_{\text{true}} \sim \text{Uniform}(-2, 2)$. When comparing estimates with MixedModels.jl under the random intercept model for the Poisson, Bernoulli and negative binomial base, smaller regression coefficients $\boldsymbol{\beta}_{\text{true}} \sim \text{Uniform}(-0.2, 0.2)$ hold. For Gaussian base, all precisions $\tau_{\text{true}} = 100$. For

the negative binomial base, all dispersion parameters are $r_{\text{true}} = 10$. Under both CS and AR(1) parameterizations of $\mathbf{\Gamma}_i$, $\sigma^2_{\text{true}} = 0.5$ and $\rho_{\text{true}} = 0.5$. Each simulation scenario was run on 100 replicates for each sample size $n \in \{100, 1000, 10000\}$ and number of observations $d_i \in \{2, 5, 10, 15, 20, 25\}$ per independent sampling unit. By default, convergence tolerances are set to 10^{-6}.

Under the VC parameterization of $\mathbf{\Gamma}_i$, the choice $\mathbf{\Gamma}_{i,\text{true}} = \theta_{\text{true}} \times \mathbf{1}_{d_i}\mathbf{1}_{d_i}^\top$ allows us to compare to the random intercept GLMM fit using MixedModels.jl. When the random effect term is a scalar, MixedModels.jl uses Gaussian quadrature for parameter estimation. We compare estimates and run-times to the random intercept GLMM fit of MixedModels.jl with 25 Gaussian quadrature points. We conduct simulation studies under two scenarios (simulation I and II). In simulation I, it is assumed that the data are generated by the approximate-copula model with $\theta_{\text{true}} = 0.1$, and in simulation II, it is assumed that the true distribution is the random intercept GLMM with $\theta_{\text{true}} = 0.01, 0.05$.

Figures 1-4 summarize the performance of the MLEs using mean squared errors (MSE) under the AR(1) parameterization of $\mathbf{\Gamma}_i$. Figures 5-8 summarize the same under the CS parameterization of $\mathbf{\Gamma}_i$. Figures 9-10 help us assess estimation accuracy and how well the GLMM density approximates the approximate-copula density under simulation I for the Bernoulli and Gaussian base. Under simulation II, Figures 11-14 shed light on how well the approximate-copula density approximates the GLMM density under different magnitudes of variance components. Figure 11 shows that for the Bernoulli base distribution with $\theta_{\text{true}} = 0.05$, the approximate-copula estimates of the variance component has average MSE of about 10^{-3}. Figure 12 shows that for the Bernoulli base distribution with $\theta_{\text{true}} = 0.01$, the approximate-copula estimates of the variance component improves to an average MSE of about 10^{-4}. Figure 13 shows for the Gaussian base distribution with $\theta_{\text{true}} = 0.05$, the approximate-copula estimates of the variance component has an average MSE around 10^{-4}, and the approximate-copula estimates of the precision has an average MSE around 10^{-2}. Figure 14 shows that for the Gaussian base distribution with $\theta_{\text{true}} = 0.01$, the approximate-copula estimates of the variance component improves to an average MSE of about 10^{-6}, and the approximate-copula estimates of the precision improves to an average MSE of about 10^{-4}. The AC model accurately estimates the mean components even with large cluster sizes ($d_i = 25$) and small sample sizes ($n = 100$), even when the true density is that of the GLMM and LMM.

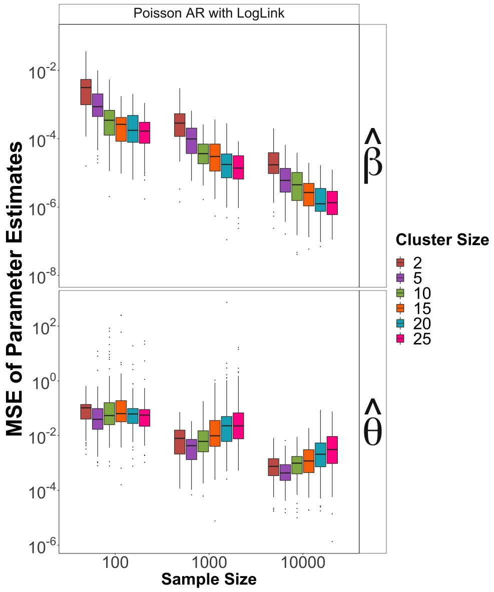

Figure A: MSE $\boldsymbol{\beta}$ and $\boldsymbol{\theta}$ under the AR(1) model for the Poisson base.

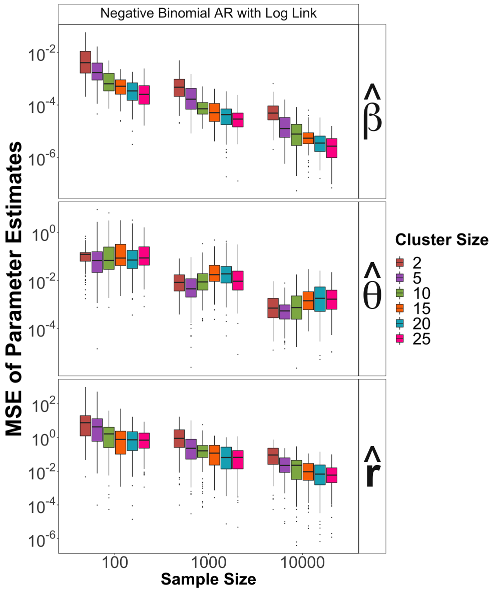

Figure B: MSE of $\boldsymbol{\beta}$ and $\boldsymbol{\theta}$ under the AR(1) model for the negative binomial base.

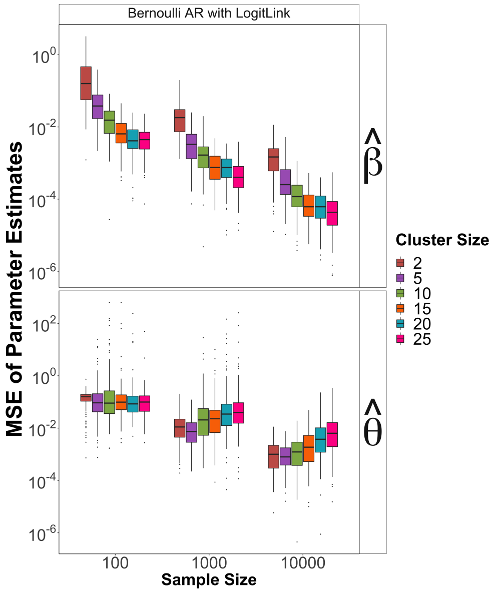

Figure C: MSE of $\boldsymbol{\beta}$ and $\boldsymbol{\theta}$ under the AR(1) model for the Bernoulli base.

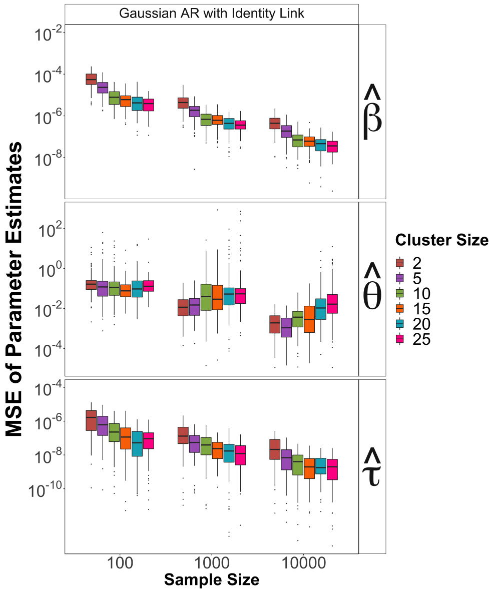

Figure D: MSE of $\boldsymbol{\beta}$ and $\boldsymbol{\theta}$ under the AR(1) model for Gaussian base.

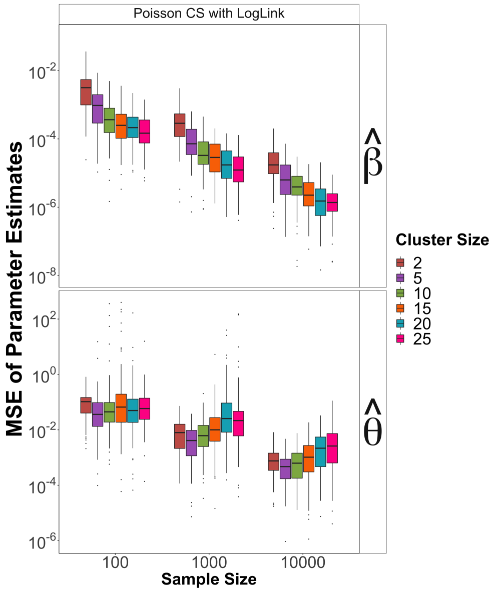

Figure E: MSE $\boldsymbol{\beta}$ and $\boldsymbol{\theta}$ under the CS model for Poisson base.

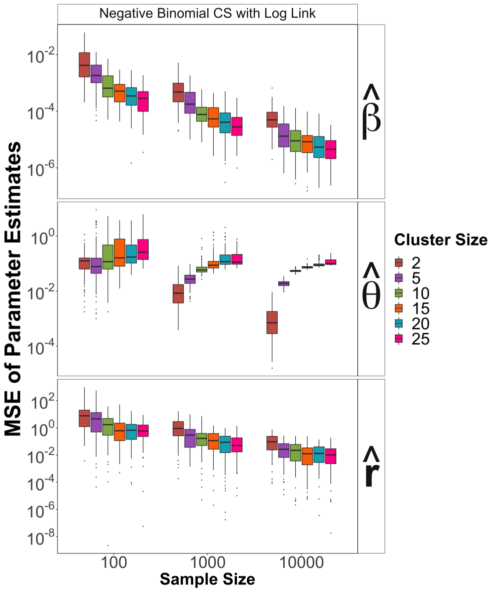

Figure F: MSE of $\boldsymbol{\beta}$ and $\boldsymbol{\theta}$ under the CS model for negative binomial base.

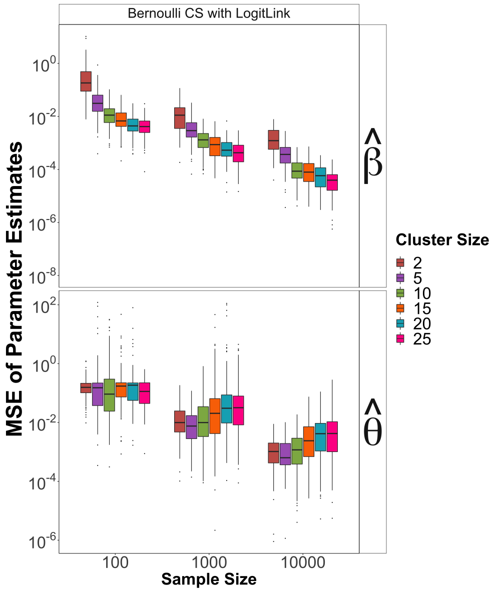

Figure G: MSE of $\boldsymbol{\beta}$ and $\boldsymbol{\theta}$ under the CS model for Bernoulli base.

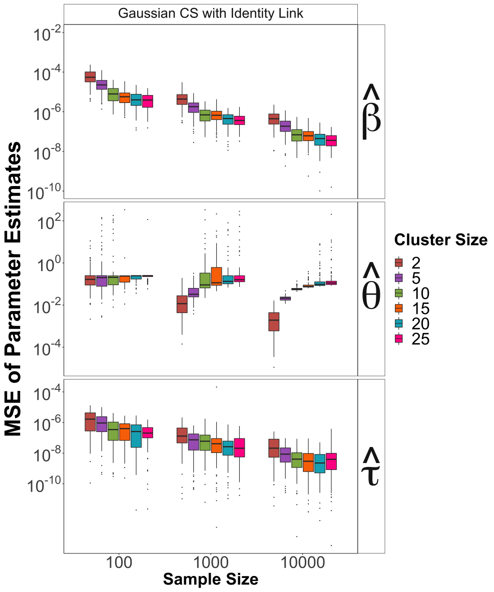

Figure H: MSE $\boldsymbol{\beta}$ and $\boldsymbol{\theta}$ under the CS model for Normal base.

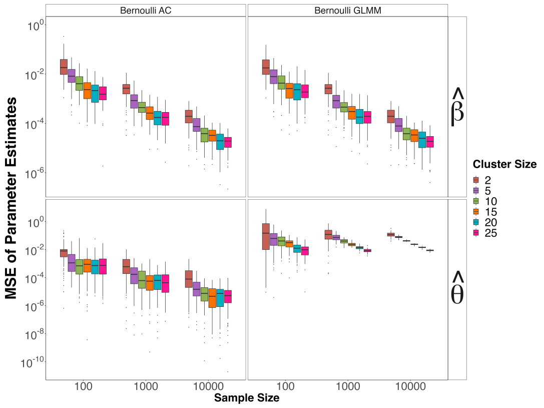

Figure I: Simulation I: MSE of $\boldsymbol{\beta}$ and $\boldsymbol{\theta}$ under Bernoulli base and a single VC.

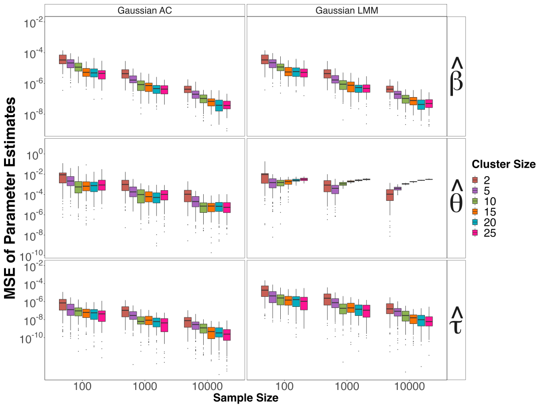

Figure J: Simulation I: MSE of $\boldsymbol{\beta}$ and $\boldsymbol{\theta}$ under the Normal base and a single VC.

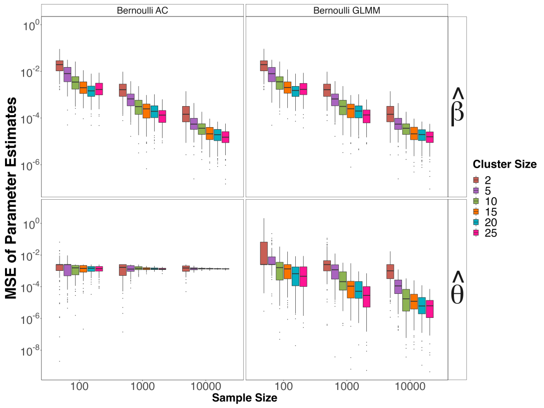

Figure K: Simulation I: MSE of $\boldsymbol{\beta}$ and $\boldsymbol{\theta}$ under the Normal base with a single VC.

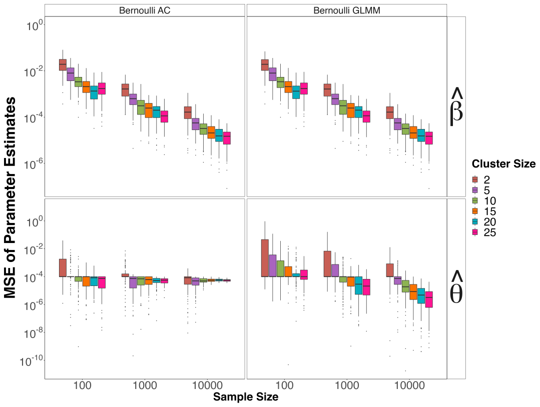

Figure L: Simulation II: MSE of $\boldsymbol{\beta}$ and $\boldsymbol{\theta} = 0.01$ under Bernoulli base and a single VC.

S1.5.1 Additional Run Times

Run times under simulation I and II are comparable. Tables 1-4 presents average run times and their standard errors in seconds, for 100 replicates under the AR(1) and CS models. Tables 5-6 present average run times and their standard errors in seconds, for 100 replicates under simulation II with $\theta_{\text{true}} = 0.01$. All computer runs were performed on a standard 2.3 GHz Intel i9 CPU with 8 cores. Runtimes for the approximate-copula model are presented using multi-threading across 8 cores.

**n**	$\mathbf{d_i}$	**Poisson AR(1) time**	**Poisson CS time**
100	2	0.057 (0.001)	0.065 (0.002)
100	5	0.069 (0.002)	0.079 (0.002)
100	10	0.112 (0.008)	0.133 (0.011)
100	15	0.214 (0.021)	0.212 (0.019)
100	20	0.238 (0.023)	0.234 (0.020)
100	25	0.307 (0.025)	0.289 (0.023)
1000	2	0.060 (0.001)	0.066 (0.001)
1000	5	0.074 (0.001)	0.081 (0.001)
1000	10	0.096 (0.001)	0.108 (0.002)
1000	15	0.112 (0.002)	0.125 (0.002)
1000	20	0.153 (0.012)	0.158 (0.008)
1000	25	0.153 (0.003)	0.180 (0.011)
10000	2	0.201 (0.002)	0.199 (0.002)
10000	5	0.271 (0.002)	0.302 (0.003)
10000	10	0.358 (0.002)	0.446 (0.004)
10000	15	0.447 (0.004)	0.564 (0.006)
10000	20	0.543 (0.005)	0.651 (0.006)
10000	25	0.703 (0.008)	0.757 (0.007)

Table A: Run times and (standard error of run times) in seconds based on 100 replicates for Poisson Base under AR(1) and CS models with sampling unit size d_i and sample size n.

n	d_i	NB AR(1) time	NB CS time
100	2	0.323 (0.009)	0.300 (0.009)
100	5	0.339 (0.007)	0.311 (0.008)
100	10	0.320 (0.008)	0.337 (0.012)
100	15	0.334 (0.011)	0.391 (0.016)
100	20	0.364 (0.013)	0.372 (0.015)
100	25	0.376 (0.016)	0.362 (0.016)
1000	2	0.445 (0.004)	0.381 (0.004)
1000	5	0.499 (0.003)	0.429 (0.004)
1000	10	0.564 (0.004)	0.520 (0.009)
1000	15	0.654 (0.010)	0.700 (0.021)
1000	20	0.798 (0.019)	0.864 (0.030)
1000	25	0.938 (0.022)	0.864 (0.030)
10000	2	2.656 (0.012)	2.297 (0.017)
10000	5	3.161 (0.013)	2.706 (0.012)
10000	10	3.875 (0.015)	4.001 (0.059)
10000	15	4.924 (0.016)	5.302 (0.140)
10000	20	6.353 (0.028)	6.073 (0.142)
10000	25	7.449 (0.109)	6.987 (0.144)

Table B: Run times and (standard error of run times) in seconds based on 100 replicates for negative binomial (NB) Base under AR(1) and CS models with sampling unit size d_i and sample size n.

n	d_i	Bernoulli AR(1) time	Bernoulli CS time
100	2	0.052 (0.002)	0.051 (0.002)
100	5	0.062 (0.002)	0.069 (0.003)
100	10	0.176 (0.019)	0.123 (0.012)
100	15	0.218 (0.021)	0.213 (0.017)
100	20	0.253 (0.022)	0.310 (0.021)
100	25	0.299 (0.024)	0.339 (0.021)
1000	2	0.080 (0.002)	0.056 (0.002)
1000	5	0.081 (0.001)	0.069 (0.002)
1000	10	0.096 (0.006)	0.088 (0.001)
1000	15	0.121 (0.006)	0.119 (0.007)
1000	20	0.179 (0.016)	0.179 (0.015)
1000	25	0.226 (0.020)	0.232 (0.020)
10000	2	0.183 (0.002)	0.171 (0.003)
10000	5	0.256 (0.002)	0.264 (0.003)
10000	10	0.304 (0.002)	0.356 (0.003)
10000	15	0.432 (0.004)	0.450 (0.004)
10000	20	0.507 (0.005)	0.535 (0.007)
10000	25	0.614 (0.005)	0.673 (0.007)

Table C: Run times and (standard error of run times) in seconds based on 100 replicates for Bernoulli Base under AR(1) and CS models with sampling unit size d_i and sample size n.

n	d_i	Gaussian AR(1) time	Gaussian CS time
100	2	0.213 (0.008)	0.214 (0.008)
100	5	0.305 (0.021)	0.338 (0.022)
100	10	0.392 (0.025)	0.432 (0.027)
100	15	0.507 (0.028)	0.441 (0.029)
100	20	0.533 (0.027)	0.448 (0.031)
100	25	0.590 (0.027)	0.429 (0.030)
1000	2	0.236 (0.006)	0.236 (0.006)
1000	5	0.272 (0.005)	0.309 (0.006)
1000	10	0.365 (0.011)	0.415 (0.010)
1000	15	0.461 (0.024)	0.547 (0.021)
1000	20	0.548 (0.028)	0.628 (0.026)
1000	25	0.561 (0.030)	0.669 (0.026)
10000	2	0.604 (0.013)	0.582 (0.011)
10000	5	0.753 (0.016)	0.793 (0.017)
10000	10	0.871 (0.015)	1.053 (0.015)
10000	15	1.032 (0.018)	1.300 (0.022)
10000	20	1.233 (0.030)	1.718 (0.025)
10000	25	1.437 (0.033)	2.191 (0.042)

Table D: Run times and (standard error of run times) in seconds based on 100 replicates for Gaussian Base under AR(1) and CS models with sampling unit size d_i and sample size n.

**n**	$\mathbf{d_i}$	**Bernoulli AC time**	**Bernoulli GLMM time**
100	2	0.048 (<0.001)	0.022 (0.002)
100	5	0.049 (0.001)	0.041 (0.001)
100	10	0.050 (0.001)	0.086 (0.004)
100	15	0.049 (0.001)	0.125 (0.005)
100	20	0.047 (0.001)	0.167 (0.005)
100	25	0.047 (0.001)	0.203 (0.008)
1000	2	0.045 (0.001)	0.166 (0.003)
1000	5	0.045 (0.001)	0.446 (0.013)
1000	10	0.043 (0.001)	0.899 (0.022)
1000	15	0.044 (0.001)	1.435 (0.038)
1000	20	0.054 (0.002)	1.888 (0.041)
1000	25	0.077 (0.002)	2.461 (0.057)
10000	2	0.138 (0.003)	1.726 (0.034)
10000	5	0.160 (0.003)	4.711 (0.099)
10000	10	0.189 (0.003)	10.389 (0.221)
10000	15	0.232 (0.003)	15.958 (0.327)
10000	20	0.276 (0.003)	21.609 (0.313)
10000	25	0.349 (0.003)	28.723 (0.494)

Table E: Run times and (standard error of run times) in seconds based on 100 replicates under simulation II with Bernoulli Base, $\theta_{\text{true}} = 0.01$, sampling unit size d_i and sample size n.

n	d_i	Gaussian AC time	LMM time
100	2	0.112 (0.002)	0.003 (0.003)
100	5	0.106 (0.003)	0.001 (<0.001)
100	10	0.097 (0.002)	0.001 (<0.001)
100	15	0.099 (0.004)	0.001 (<0.001)
100	20	0.105 (0.008)	0.001 (<0.001)
100	25	0.109 (0.008)	0.003 (0.002)
1000	2	0.110 (0.002)	0.004 (0.002)
1000	5	0.103 (0.002)	0.002 (<0.001)
1000	10	0.100 (0.002)	0.006 (0.002)
1000	15	0.095 (0.001)	0.006 (0.001)
1000	20	0.094 (0.002)	0.008 (0.001)
1000	25	0.099 (0.002)	0.011 (0.002)
10000	2	0.200 (0.005)	0.018 (0.003)
10000	5	0.192 (0.004)	0.029 (0.003)
10000	10	0.216 (0.004)	0.050 (0.004)
10000	15	0.219 (0.003)	0.067 (0.003)
10000	20	0.239 (0.003)	0.091 (0.003)
10000	25	0.258 (0.002)	0.099 (0.003)

Table F: Run times and (standard error of run times) in seconds based on 100 replicates under simulation II with Gaussian Base, $\theta_{\text{true}} = 0.01$, sampling unit size d_i and sample size n.

S1.6 Additional simulations for multivariate model

S1.6.1 Is the SNP screening procedure valid?

Recall that Algorithm 1 only runs likelihood ratio tests on the most promising SNPs and employs an early stopping criteria when the current likelihood ratio p-value falls below some early termination threshold. The screening procedure relies on computing SNP gradients under the null model, given the intuition that a causal SNP should have large non-zero effect. Figure M verifies this intuition in simulated data, where the simulation procedure follows Simulation III of the main paper except we choose $k = 0$ causal SNPs. As shown in the plot, a "larger" gradient (as measured by average absolute value) tend to ultimately produce a large $-\log_{10}$(p-value) i.e. be more significant under likelihood ratio test, with correlation coefficient 0.885.

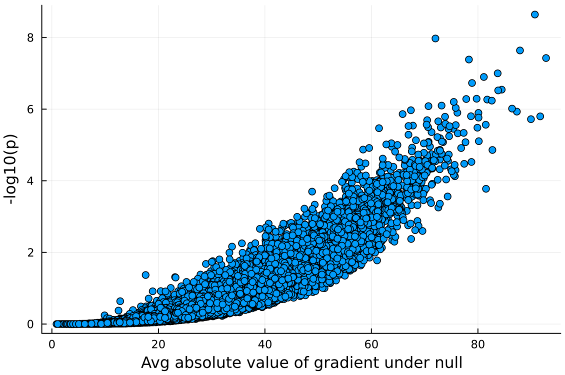

Figure M: A comparison of the gradient of the SNP effect under the null model to their final p-values. Larger gradients tend to produce smaller p-values.

S1.6.2 QQ plots

To verify that p-values from testing the null (equation (5) in the main text) are valid, we conducted a simulation study with $n = 5000$ samples, $d = 4$ phenotypes (2 Gaussian, 1 Bernoulli, and 1 Poisson), $p = 15$ non-genetic covariates, and $q = 20000$ SNPs. The matrix $\mathbf{\Gamma}$ was defined as the AR1 model with $\rho = 0.5$ and $\sigma^2 = 1$. Genotype value x_{ij} for sample i at SNP j were simulated as the sum of 2 independent Bernoulli(0.3) draws. The non-genetic covariate were simulated from $N(0, 1)$ and the first column set to 1. Non-genetic effects were simulated from Uniform$(-0.5, 0.5)$. All genetic effects are 0. The mean of each sample $\boldsymbol{\mu}_i$ was formed by applying the inverse canonical link (identity, logit, log) for each base distribution to the linear predictors $\boldsymbol{\eta}_i = \boldsymbol{X}_i\boldsymbol{\beta}$. Finally, sample phenotypes $\boldsymbol{y}_i$ were simulated under the approximate-copula model for each subject i independently.

Under this setup, we ran Algorithm 1 on the resulting SNPs with early stopping criteria set to infinity (i.e. no early stopping). The resulting p-values are displayed in a QQ plot in Figure N. The plot meets our expectations and suggests that our estimation procedure is working properly.

S1.7 Additional results from the NHANES logntudinal analysis

In Table 3 of the main text, we compared parameter estimates for negative binomial bases under approximate copulas, GLMM, and GEE. In Table G, we additionally include comparisons for same analysis but using Poisson base distribution.

	Estimate	Std. Error	z	Pr(> $\|z\|$)	Lower 95%	Upper 95%
**AC**						
(Intercept)	2.509	0.205	12.24	<1e-99	2.107	2.910
sex	-0.210	0.050	-4.232	<2.32e-5	-0.307	-0.113
age	-0.009	0.046	-0.193	0.845	-0.10	0.966
price	0.434	0.271	1.60	0.110	-0.010	0.966
θ	7.080	0.787	8.993	<1e-99	5.537	8.623
**GLMM**						
(Intercept)	2.040	0.084	24.22	<1e-99	1.727	2.351
sex	-0.225	0.036	-6.24	<1e-9	-0.359	-0.091
age	-0.009	0.002	-6.19	<1e-9	-0.015	-0.004
price	0.596	0.023	26.42	<1e-99	0.513	0.681
θ	0.458	NA	NA	NA	NA	NA
**GEE**						
(Intercept)	2.361	0.108	21.85	<1e-99	2.149	2.573
sex	-0.194	0.030	-6.39	<1e-9	-0.253	-0.134
age	-0.007	0.001	-6.3	<1e-9	-0.010	-0.005
price	0.486	0.044	11.14	<1e-28	0.40	0.571
θ	NA	NA	NA	NA	NA	NA

Table G: Comparisons of parameter estimates on the NHANES data under Poisson approximate-copula (AC) model, GLMM, and GEE. All $n = 1537$ sampling units are of size $d_i = 2$. Here θ is the variance component.

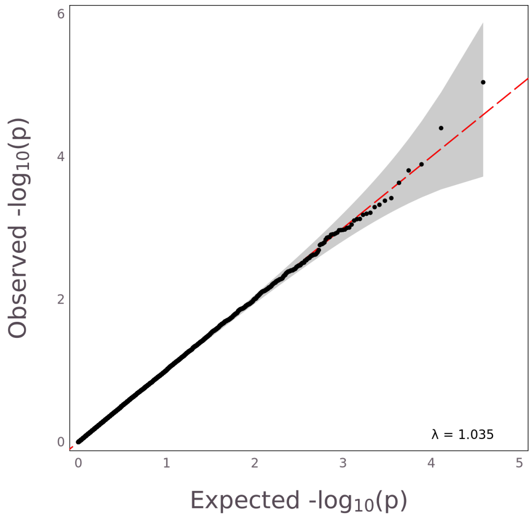

Figure N: QQ plot of p-values resulting from Algorithm 1 without early exiting.

S1.8 Quality control on UK-Biobank genotypes

We jointly analyzed BMI, SBP, and DBP on the UK-Biobank. For illustrative purposes, SBP and DBP were dichotomized as outlined in Section 3.5. BMI was log-transformed to minimize the impact of outliers and then standardized to mean 0 and variance 1.

Following our past analyses (German et al., 2020; Chu et al., 2020, 2023; Ko et al., 2022), we filtered out subjects exhibiting sex discordance, high heterozygosity, or high SNP missingness according to recommendation by UK-Biobank. We then excluded subjects of non-European ancestry and first and second-degree relatives based on empirical kinship coefficients. We also excluded subjects on hypertension medicine at baseline. Finally, we excluded subjects with $< 98\%$ genotyping success rate and SNPs with $< 99\%$ genotyping success rate and imputed the remaining missing genotypes by the corresponding sample-mean genotypes.

Given these reduced data and ignoring the Biobank's precomputed principal components, we computed afresh the top 5 principal components of the genotype matrix via `FlashPCA2` (Abraham et al., 2017). These principal components serve as predictors to adjust for hidden ancestry. We also designated sex and age as non-genetic predictors. The final data included $470,228$ SNPs and $185,656$ subjects. For faster computation time, we randomly selected $n = 80000$ samples to perform the analysis.

S1.9 Additional simulations for negative binomial longitudinal models

Finally, we also compared our negative binomial fits with those delivered by the three popular R packages for GLMM estimation in Tables H. On a single dataset with $d_i = 5$, and $n = 10,000$ simulated under simulation II, the lme4 package (Bates et al., 2015) takes an inordinately long time to fit the model. Obtaining confidence intervals takes a significant amount of additional time, and inference of r is impossible. The glmmTMB package (Brooks et al., 2017) allows for inference of r and takes much less time to form confidence intervals than lme4, but it is still significantly slower than approximate-copula fitting. Both lme4 and glmmTMB fit the negative binomial GLMM using Laplace Approximation, while the GLMMadaptive package (Pinheiro and Bates, 1995) uses adaptive Gaussian quadrature. In Tables H and I, we use GLMMadaptive to fit the data with 25 Gaussian quadrature points. GLMMadaptive allows for inference of r and takes no additional time to form confidence intervals, but is still significantly slower than approximate-copula fitting. Run times in seconds for obtaining the estimates and confidence intervals in Table H appear in Table I.

**Parameter**	**Truth**	**AC fit**	**lme4 fit**	**glmmTMB fit**	**GLMMadaptive fit**
β_1	0.036	0.033	0.032	0.033	0.032
		(0.028, 0.037)	(0.022, 0.042)	(0.023, 0.043)	(0.023, 0.042)
β_2	0.107	0.106	0.106	0.106	0.106
		(0.101, 0.111)	(0.097, 0.115)	(0.097, 0.115)	(0.097, 0.115)
β_3	0.026	0.026	0.026	0.026	0.026
		(0.017, 0.035)	(0.017, 0.035)	(0.017, 0.035)	(0.017, 0.035)
θ	0.01	0.007	0.009	0.008	0.009
		(0.003, 0.011)	(0.002, 0.015)	(0.003, 0.019)	(0.005, 0.018)
r	10	10.002	10.147	10.101	9.996
		(9.094, 10.910)	(NA, NA)	(8.640, 11.809)	(8.612, 11.602)

Table H: MLE's and (confidence intervals) based on a single replicate under simulation II with negative binomial Base, $\theta_{\text{true}} = 0.01$, sampling unit size $d_i = 5$ and sample size $n = 10000$.

**n**	**d_i**	**AC time**	**lme4 time**	**glmmTMB time**	**GLMMadaptive time**
10000	5	1.247 (0.046)	75.774 (158.034)	98.944 (0.472)	84.471 (<0.001)

Table I: Run times and (confidence interval run times) in seconds based on a single replicate under simulation II with negative binomial Base, $\theta_{\text{true}} = 0.01$, sampling unit size $d_i = 5$ and sample size $n = 10000$.

S1.10 Cauchy combination test as an alternative to multivariate GWAS

Here we provide a complementary analysis to the multivariate GWAS application in our main paper. An alternative approach to jointly modeling the 3 traits would be to conduct a univariate GWAS for each outcome separately and then aggregate the results using the Cauchy combination test (Liu and Xie, 2020). Here we explore this alternative analysis option. Specifically, we used the PLINK2 software, with BMI treated as a continuous trait and SBP/DBP both dichotomized in the same way as featured in our paper. Then we combined the p-values using Cauchy's combination tests, with all weights set equal to 1/3. The resulting Manhattan plot is shown in Figure O.

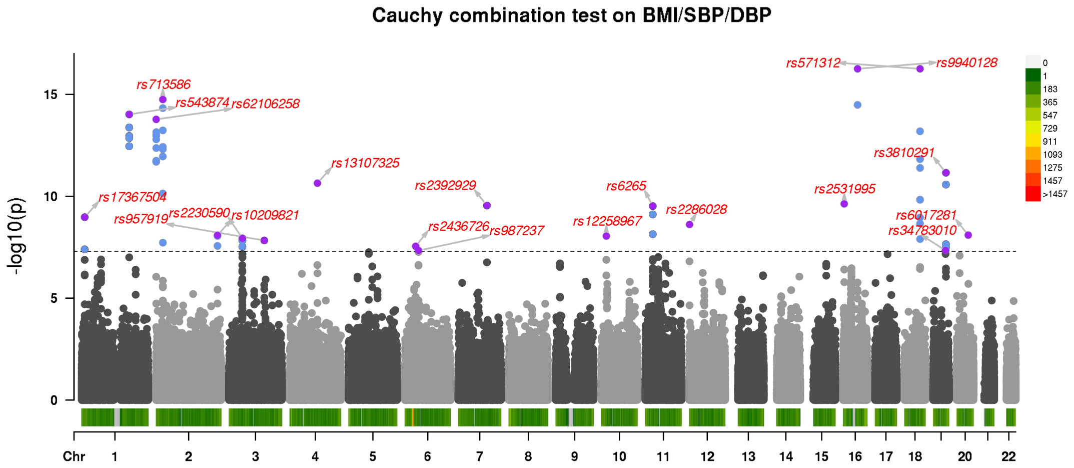

Figure O: Applying Cauchy combination test to merge 3 univariate GWAS results.

Comparing this result to our approximate copula based GWAS (Figure 3 in main paper), we observe that:

- The total number of independent discoveries is higher in the approximate copula model (24 discoveries) than in the Cauchy combination test (20 discoveries).
- Some discoveries overlap (e.g. rs17367504 and rs173586 in chromosome 1), while a couple of SNPs do not. This is expected, since we label the most significant p-value in a 1Mb window, and the 2 methods could have picked different SNPs in the same genomic neighborhood. Inspecting the location of

the significant regions suggest that the two results agree generally where the significant regions are located.

Although this result is promising, note that the comparison itself is not perfect, since we are comparing a **standard GWAS pipeline** (standard linear/logistic regression with Wald-test p-values aggregated via Cauchy combination test) to that of our approximate copula model (which utilizes likelihood ratio tests). Therefore, the results presented here is a simple proof-of-principle work. Nevertheless, it shows that explicitly modeling the joint-trait covariance within the approximate copula framework has the potential to bring additional power for association.

References

Abraham, G., Qiu, Y., and Inouye, M. (2017). FlashPCA2: principal component analysis of biobank-scale genotype datasets. *Bioinformatics*, 33(17):2776–2778.

Bates, D., Mächler, M., Bolker, B., and Walker, S. (2015). Fitting linear mixed-effects models using lme4. *Journal of Statistical Software*, 67(1):1–48.

Brooks, M. E., Kristensen, K., van Benthem, K. J., Magnusson, A., Berg, C. W., Nielsen, A., Skaug, H. J., Maechler, M., and Bolker, B. M. (2017). glmmTMB balances speed and flexibility among packages for zero-inflated generalized linear mixed modeling. *The R Journal*, 9(2):378–400.

Chu, B. B., Keys, K. L., German, C. A., Zhou, H., Zhou, J. J., Sobel, E. M., Sinsheimer, J. S., and Lange, K. (2020). Iterative hard thresholding in genome-wide association studies: Generalized linear models, prior weights, and double sparsity. *GigaScience*, 9(6):giaa044.

Chu, B. B., Ko, S., Zhou, J. J., Jensen, A., Zhou, H., Sinsheimer, J. S., and Lange, K. (2023). Multivariate genome-wide association analysis by iterative hard thresholding. *Bioinformatics*, 39(4):btad193.

German, C. A., Sinsheimer, J. S., Klimentidis, Y. C., Zhou, H., and Zhou, J. J. (2020). Ordered multinomial regression for genetic association analysis of ordinal phenotypes at biobank scale. *Genetic Epidemiology*, 44:248–260.

Ko, S., German, C., Jensen, A., Shen, J., Wang, A., Mehrotra, D., Sun, Y. V., Sinsheimer, J. S., Zhou, H., and Zhou, J. (2022). GWAS of longitudinal trajectories at biobank scale. *The American Journal of Human Genetics*, 109(3):433–445.

Lange, K. (2016). *MM Optimization Algorithms*. SIAM.

Lange, K., Hunter, D. R., and Yang, I. (2000). Optimization transfer using surrogate objective functions. *Journal of Computational and Graphical Statistics*, 9(1):1–20.

Liu, Y. and Xie, J. (2020). Cauchy combination test: a powerful test with analytic p-value calculation under arbitrary dependency structures. *Journal of the American Statistical Association*, 115(529):393–402.

Pinheiro, J. C. and Bates, D. M. (1995). Approximations to the log-likelihood function in the nonlinear mixed-effects model. *Journal of Computational and Graphical Statistics*, 4(1):12–35.

Zhou, H., Hu, L., Zhou, J., and Lange, K. (2019). MM algorithms for variance components models. *Journal of Computational and Graphical Statistics*, 28(2):350–361.
