# Supplementary material for: An approximate-copula distribution for statistical modeling
Source: PLoS Comput Biol. 2026 Mar 13;22(3):e1013922. doi: 10.1371/journal.pcbi.1013922 (PMC12998956; doi:10.1371/journal.pcbi.1013922)
Supplement: Attachment — Submitted filename: Response to the Editors.pdf [file pcbi.1013922.s002.pdf]

# Response to the Editors

We thank the editors for our email correspondences, and in particular, for pointing out that we should avoid the term "quasi-copula" in our paper. The Editor specifically commented:

> *"The proposed density does not appear to be a quasi-copula. Please use a suitable term to prevent it from being evaluated as a new quasi-copula."*

We have therefore renamed the proposed distribution into "An Approximate Copula Distribution". We have modified all text, figures, and tables in our paper to reflect this change. We also made similar adjustments to our software and reproducibility scripts, although the package renaming from "QuasiCopula.jl" to "ApproxCopula.jl" will take some time to be approved since it currently resides within the larger OpenMendel ecosystem.

We hope the updated manuscript now clearly conveys that the proposed distribution is a *new* class of multivariate distribution with unique properties, which we have thoroughly evaluated in terms of its advantages and limitations.
