# Supplementary material for: An approximate-copula distribution for statistical modeling
Source: PLoS Comput Biol. 2026 Mar 13;22(3):e1013922. doi: 10.1371/journal.pcbi.1013922 (PMC12998956; doi:10.1371/journal.pcbi.1013922)
Supplement: Attachment — Submitted filename: Review.pdf [file pcbi.1013922.s003.pdf]

Referee report on: "An Approximate-Copula Distribution for Statistical Modeling"'

The authors proposed a new statistical distribution called "approximate copula distribution" to model multivariate correlated continuous and/or categorical data. Compared to currently available tools (GLMM, GEE, and copula), the proposed distribution has computational advantage as it allows explicit calculation of moments, marginal and conditional distributions, and the score and observed information needed in maximum likelihood estimation. However, I have major concerns about the proposed method especially on its comparison with existing methods and usage in real applications.

1. It is useful to clarify the relationship between the approximate copula distribution and the copula. The authors commented that "$\mathbf{g}(x)$ is technically not a copula because it only approximately preserves the marginal distributions." More details or explanations will be useful for readers to better understand the similarity and difference between the approximate copula distribution and the copula.

2. In the numerical studies, the authors only compared their approach with GLMM. It is known that GEE is computationally more efficient than GLMM. In addition, the approximate copula distribution is also related to copula. The authors should consider to include GEE and copula in their numerical studies (both simulation and real data) for a more comprehensive comparison.

3. In Section 3.4, the authors compared the estimated coefficients, log-likelihood, and run time between the approximate copula distribution and the GLMM. The estimated coefficients are comparable. It is also interesting to compare the uncertainty of the estimates, such as the standard deviation and p-value, especially as the authors commented that the proposed distribution "tends to inflate marginal variances."

4. In Section 3.5, the authors only used the approximate copula distribution to analyze the data. Similar to previous comments, comparison with existing methods are needed. In addition, the analysis is less appealing given that the authors purposely dichotomized two continuous traits. Are there more natural choices of continuous and categorical traits for the proposed distribution in the real application?
