# Supplementary material for: An approximate-copula distribution for statistical modeling
Source: PLoS Comput Biol. 2026 Mar 13;22(3):e1013922. doi: 10.1371/journal.pcbi.1013922 (PMC12998956; doi:10.1371/journal.pcbi.1013922)
Supplement: Attachment — Submitted filename: report.pdf [file pcbi.1013922.s004.pdf]

Review of "An Approximate-Copula Distribution for Statistical Modeling" (PCOMPBIOL-D-25-00101R1)

This paper introduces a new family of multivariate distributions for the joint modeling of non-Gaussian data, such as those arising in longitudinal data and multi-trait GWAS. The family of distributions is derived from a Gaussian approximation of Tonda (2005) to the likelihood in GLMMs. The paper gives explicit expressions for the moments and marginal and conditional distributions, and develops maximum likelihood procedures for the family, extending the theory and inference procedures of Tonda (2005).

While this family of distributions can be a useful alternative to GLMMs, I have major concerns about its connection with existing methods, motivations, and usefulness.

1. The idea is in fact similar to the Laplace approximation for inference in GLMMs. The difference is about which point the Taylor expansion is obtained. The Laplace approximation expands around the conditional mode of the random effect, while the current method expands around the mean of the random effect; see the derivations in Tonda (2005, page 335). It is not immediately clear to me which approximation strategy would be more accurate, although the latter is slightly easier to implement. It would be helpful to see a detailed and thoughtful discussion on the connection and comparison of the two methods.

2. The NHANES data for longitudinal analysis and the UK Biobank data for multi-trait GWAS have dimensions $d = 2$ and $d = 3$, respectively. With such a small d, a GLMM can be easily fit. What is the motivation for adopting the new modeling approach?

3. Page 3, "we find that the model reflects reality best when the number of components of each independent sampling units is low or the correlations between responses within a unit are small": This seems contrastive to the motivation discussed in Section 1.1, in particular, scalability in high dimensions.

4. The simulation study fits GLMMs using MixedModels.jl, mentioning only that "[w]hen the random effect term is a scalar, MixedModels.jl uses Gaussian quadrature for parameter estimation." How about the multivariate case? More information about the algorithm implemented by MixedModels.jl should be provided. In particular, it would be more interesting to compare the proposed method with GLMM fitting with Laplace approximation rather than Gaussian quadrature.

5. Please comment on the convexity and convergence of the proposed algorithm. I doubt if the optimization problem is convex when both the mean and covariance parameters need to be estimated.

6. The derivations in Sections 2.3–2.5 are lengthy and straightforward, and it is unclear how they are used in the estimation procedures. This part seems to be mainly of theoretical interest and can be greatly shortened.
