# Supplementary material for: An approximate-copula distribution for statistical modeling
Source: PLoS Comput Biol. 2026 Mar 13;22(3):e1013922. doi: 10.1371/journal.pcbi.1013922 (PMC12998956; doi:10.1371/journal.pcbi.1013922)
Supplement: Attachment — Submitted filename: response.pdf [file pcbi.1013922.s007.pdf]

Approximate Copula Revision round 2

November 2025

We thank reviewer 3 for carefully going through our response and for providing helpful comments and suggestions. We have added significant verbiage in our manuscript (highlighted in red) that clarifies the practical advantages and limitations of the approximate-copula model. Detailed point-to-point responses can be found below. We hope these responses fully address the reviewer's concerns.

Reviewer 3 comments

1. In response to Comment 1, the authors wrote that "the entry γ_{ij} is neither the covariance nor the correlation between Y_i and Y_j." However, in response to Comment 2, they stated that "as mentioned in the response to the previous question, Γ is a covariance matrix." Could the authors please clarify this?

We apologize for the confusion. When we wrote "covariance" matrix in the context of responding to comment 2, we simply meant a matrix that is symmetric and positive semi-definite. In our day-to-day conversation the two has became interchangeable.

For full clarity, the previous reviewer's comment was

> *The moment calculations in Section 2.3 seem to rely on the assumption that the spectral norm of Γ is small. If Γ represents a correlation matrix, we can assume that all its diagonal entries are 1. In that case, the spectral norm of Γ depends heavily on the off-diagonal entries—that is, the correlations between outcomes. In the simplest case, where all outcomes are uncorrelated, the spectral norm is 1. However, in extreme cases with strong correlations, the spectral norm can be quite large. This implies that assuming a small spectral norm may restrict the strength of allowable correlations between outcomes. In other words, when the outcomes are highly correlated, the marginal distribution of Y could differ substantially from that of X.*

And the fully clarified response should read:

This insightful comment recalls the considerable effort we put into developing model. $\mathbf{\Gamma}$ provides a mechanism for capturing both trait correlation and over-dispersion. As mentioned in the response to the previous question, **the only requirement on $\mathbf{\Gamma}$ is that it is symmetric and positive semi-definite**. Thus, its diagonal elements can be arbitrarily large or small. Large diagonal entries of $\mathbf{\Gamma}$ suggest over-dispersion. Large off-diagonal entries suggest trait dependence. If neither complication is present in the data, then standard univariate GLM (base) models are adequate. However, it is imprudent to simply ignore the possibility of dependence or over-dispersion. In any event the equality

$$\mathrm{Cov}(Y_k, Y_l) \quad = \quad \left[1 + \frac{1}{2}\mathrm{tr}(\mathbf{\Gamma})\right]^{-1} \sigma_k \sigma_l \gamma_{kl}$$

accurately captures the added covariance when $\mathbf{\Gamma}$ enters the picture, whether $\|\mathbf{\Gamma}\|$ is large or not.

The formerly confusing paragraph now reads in part

> The virtue of the density (1) is that it overcomes the independence restriction and steers the estimated covariance matrix toward the sample covariance matrix of the residuals. Inclusion of the positive semidefinite matrix $\mathbf{\Gamma}$ is designed to achieve this goal. For example, if X_1 is Gaussian and X_2 is Bernoulli, then the density allows one to induce correlation between the two components (one continuous and the other discrete) of the binary random vector $X = (X_1, X_2)^\top$. Note that $g(\mathbf{x})$ is technically not a copula because it only approximately preserves the marginal distributions $f_i(x_i)$. Later, we will see how $g(\mathbf{x})$ tends to inflate marginal variances and accommodate correlation. This can be a blessing rather than a curse if correlation is present or the true marginal distributions are over-dispersed compared to the assumed marginal distributions.

2. I appreciate the clarification regarding the approximate copula model and its impact on preserving the marginals of X. However, it remains unclear how this approximation might affect the overall performance of the proposed method. The authors mention that the approach tends to perform well when correlations between outcomes are weak (as noted in their response to Comment 3: "this assumption may also be reasonable, especially when the observed correlation is weak"). Could the authors elaborate on what this means in practice? For instance, should practitioners consider using this method primarily when correlations are weak? The current presentation is mathematically detailed, but it would greatly benefit from clearer guidance for applied researchers regarding the strengths and limitations of the method.

This is a fine suggestion. We agree that the paper can benefit from a clearer discussion of the advantages and limitations of the proposed approximate copula model, and giving more practical guidance for applied researchers. Accordingly, we have substantially expanded the **Discussion section** to address these points. In particular, we added two new paragraphs (shown below) that clarify when

the method is most appropriate, its key strengths and weaknesses, and how it complements standard approaches such as GLMM and GEE.

> The approximate-copula model shines when GLMM and GEE are difficult to apply or yield unsatisfactory results. We illustrate this fact for longitudinal binary traits and multiple correlated traits of mixed types. To its credit, the approximate-copula model is likelihood-based, preserves base distributions approximately, and enables hypothesis testing of mean effects and correlations. However, our numerical tests suggest caution. In particular, performance may degrade when correlations between responses are strong or when cluster sizes are large. In this setting the normalizing constant $\ln[1 + \frac{1}{2}\operatorname{tr}(\boldsymbol{\Gamma})]$ grows and tends to shrink estimates of $\boldsymbol{\Gamma}$, making variance component estimates unreliable. In defense of the model, our limited evidence suggests that estimates of mean effects are hardly affected. We suspect that likelihood ratio tests of correlation are also reliable. Given the multivariate nature of the model, it also enhances statistical precision and power in the analysis of mean effects when correlations exist among the traits. In our opinion, variance components are nuisance parameters compared to mean effects in important examples such as GWAS.
>
> In any case, we recommend univariate analysis by standard statistical tools as a prelude to multivariate analysis under the approximate-copula model. Understanding the nature of complicated data is best served when the data is approached from several angles. Despite its simplifications, the approximate-copula model's balance of speed, interpretability, and flexibility make it a valuable complement to existing methods. We hope that other statisticians will agree, work to improve its performance, and apply it to their own data.

3. I appreciate the authors' comparison with univariate GWAS and their careful interpretation of results. However, from a practitioner's perspective, it is still unclear why one should choose this method over the simpler univariate GWAS approach, particularly if it may not provide higher power or stronger biological insights. It would be helpful if the authors could articulate more concretely the advantages of the proposed approach in real applications. PLOS Computational Biology values methodological innovation that provides meaningful biological or practical impact; without such clarification, the application section appears as a technical illustration rather than a demonstration of real-world utility.

We agree with the reviewer's suggestion of adding exposition to better explain the practical advantages of our method versus alternative approaches. Please see the added paragraphs to the discussion section given in the answer to the previous question.

As a concrete example of the model's value, note that our approximate-copula GWAS yielded 4 additional discoveries compared to standard univariate GWAS + Cauchy combination tests. Therefore, our proposed method may well increase power relative to univariate analysis, which in principle translates to stronger biological insights. (These results have now been incorporated in supplemental

Section 1.10 of the manuscript.) To aid the reviewer in understanding the improvement in power, recall that in the last round of revision, we followed the suggestion of running 3 univariate GWASes and subsequently combined the p-values using Cauchy's combination test. We wrote:

> The total number of independent discoveries is higher in the approximate copula model (24 discoveries) than in the Cauchy combination test (20 discoveries).

For the convenience of the reviewer, we provide below 2 Manhattan plots illustrating our findings. The difference in vertical scales is telling.

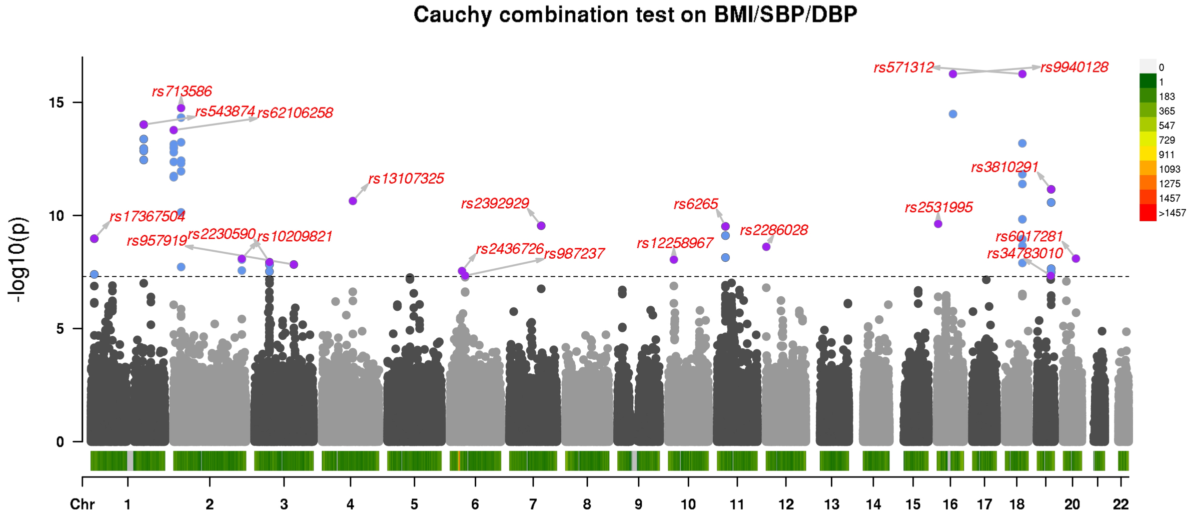

Figure 1: Applying Cauchy combination test to merge 3 univariate GWAS results. There are 20 independent discoveries.

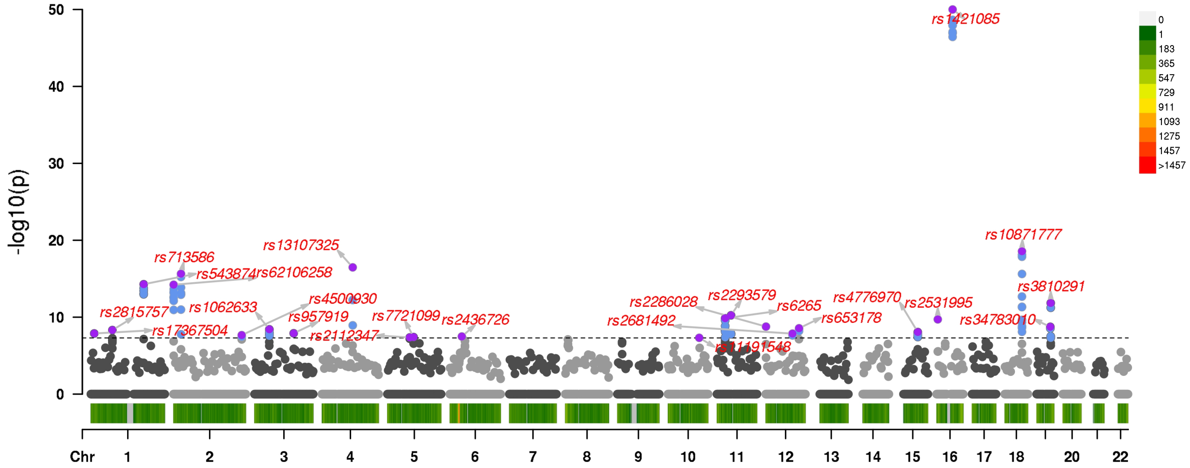

Figure 2: The 3-trait multivariate GWAS under the approximate Copula model. There are 24 independent discoveries.

4. Finally, the authors should carefully review the revised manuscript, as there are still several places where question marks remain in the text.

These mistakes have been corrected in the updated manuscript.
