# Supplementary material for: An approximate-copula distribution for statistical modeling
Source: PLoS Comput Biol. 2026 Mar 13;22(3):e1013922. doi: 10.1371/journal.pcbi.1013922 (PMC12998956; doi:10.1371/journal.pcbi.1013922)
Supplement: Attachment — Submitted filename: response_auresp_5.pdf [file pcbi.1013922.s008.pdf]

Approximate Copula Response to Editor and Reviewers

January 2026

We thank the editor and the 3 reviewers for their helpful comments and suggestions. Detailed point-to-point responses to each comment can be found below. We hope these responses fully address the editor and reviewer's concerns.

Editor

The definition of Gamma is inconsistent throughout the paper and the authors' responses. The authors stated that Gamma is neither a covariance nor a correlation matrix. However, Γ_i in sections 2.6, 2.7, 2.9, and 3.1 are stated and defined as a covariance matrix. Please clarify what Gamma is and use the term consistently throughout the paper. Also, what does m indicate in the expression of Γ_i on page 10, i.e., $\Gamma_i = \sum_{j=1}^{m} \theta_i \Omega_{ij}$?

We thank the editor for providing helpful comments and suggestions. We have made clarifications in our manuscript that clarifies Gamma is a positive semidefinite matrix and we have consistently used this term throughout the paper. The changes to the manuscript are highlighted in red.

Regarding the question about what m indicates in the expression on page 10, we have modified the text to read:

> Because each subject i can be measured at a different number d_i of time points, the choice of positive semidefinite matrices Γ_i is constrained. We explore three structured

options:

$$
\begin{aligned}
\Gamma_i &= \sum_{j=1}^{m} \theta_j \Omega_{ij} \\
\Gamma_i &= \sigma^2 \times \begin{bmatrix} 1 & \rho & \rho^2 & \rho^3 & \ldots & \rho^{d_i-1} \\ \rho & 1 & \rho & \rho^2 & \ldots & \\ & & \ldots & & & \\ & & \ldots & \rho & 1 & \rho \\ \rho^{d_i-1} & \rho^{d_i-2} & \ldots & \rho^2 & \rho & 1 \end{bmatrix} \\
\Gamma_i &= \sigma^2 \times \left[\rho \mathbf{1}_{d_i} \mathbf{1}_{d_i}^\top + (1-\rho) I_{d_i}\right].
\end{aligned}
$$

These traditional choices define the variance component (VC) model decomposing $\mathbf{\Gamma}_i$ into a linear combination of m known positive semidefinite matrices $\mathbf{\Omega}_{ij} = (\omega_{ijkl})$ against unknown nonnegative variance components $\boldsymbol{\theta} = (\theta_1, \ldots, \theta_m)$, the auto-regressive (AR1) model, and the compound symmetric (CS) model. The $\theta_i \geq 0$ must be estimated in the VC model, while $\sigma^2 \geq 0$ and ρ must be estimated in the AR1 and CS models. Section 2.9.2 describes how estimation is performed. The $\mathbf{\Gamma}_i$ parameters supplement the regression coefficients $\boldsymbol{\beta}$ of the base distributions.

Reviewer 1

The authors proposed a new statistical distribution called "approximate copula distribution" to model multivariate correlated continuous and/or categorical data. Compared to currently available tools (GLMM, GEE, and copula), the proposed distribution has computational advantage as it allows explicit calculation of moments, marginal and conditional distributions, and the score and observed information needed in maximum likelihood estimation. However, I have major concerns about the proposed method especially on its comparison with existing methods and usage in real applications.

1. It is useful to clarify the relationship between the approximate copula distribution and the copula. The authors commented that "g(x) is technically not a copula because it only approximately preserves the marginal distributions." More details or explanations will be useful for readers to better understand the similarity and difference between the approximate copula distribution and the copula.

We thank the reviewer for this comment. The main distinction between the approximate copula model and ordinary copulas is that copulas preserve the marginals of the base distribution while our model only approximately preserves marginals. Sections 2.2-2.4 of the paper make this distinction clear. Our approximate copula converges in distribution to the base model as the Γ matrix tends to zero in norm. The fact that convergence extends to first and second-order moments is explicitly mentioned in Section 2.4 of the paper.

2. In the numerical studies, the authors only compared their approach with GLMM. It is known that GEE is computationally more efficient than GLMM. In addition, the approximate copula distribution is also related to copula. The authors should consider to include GEE and copula in their numerical studies (both simulation and real data) for a more comprehensive comparison.

We thank the reviewer for the comment. Our response to this question can be broken down into 3 main points:

- For comparisons against **GEE and GLMM**, we have expanded our analysis of the NHANES data and the multivariate GWAS data. The new results appear in Sections 3.4 and 3.5 in the revised manuscript. Further details can be found in our responses to Reviewer 1's question 3 (for GEE) and question 4 (for GLMM).
- Comparison against standard **copulas** is challenging for two reasons. First, copulas are not typically used in the same regression context. As far as we know, there is no current software that supports the kind of longitudinal/multivariate regressions featured in our paper. Second, computing with copulas in even moderate dimensions is notoriously slow.
- We have omitted additional **simulation comparisons** of our model against GEEs and only provide real-data comparisons against GEEs as mentioned under the first bullet point above. First, the paper is already very long (11 tables, 17 Figures, 70+ pages). Second, because GEE's lack a well defined loglikelihood, it is unclear how to simulate under the GEE framework. If we simulate under our our model and fit using both GEE and our model, we expect our model to produce a better fit because it aligns with the data generative process. Thus, the more relevant simulation study is between GLMM and our model.

3. In Section 3.4, the authors compared the estimated coefficients, log-likelihood, and run time between the approximate copula distribution and the GLMM. The estimated coefficients are comparable. It is also interesting to compare the uncertainty of the estimates, such as the standard deviation and p-value, especially as the authors commented that the proposed distribution "tends to inflate marginal variances."

We thank the reviewer for this suggestion. In the paper, we have updated Section 3.4 to include two additional tables with these comparisons. These tables are also provided below to aid the reviewer.

	Estimate	Std. Error	z	Pr(> $|z|$)	Lower 95%	Upper 95%
**AC**						
(Intercept)	2.580	0.098	26.22	<1e-99	2.387	2.773
sex	-0.187	0.067	-2.804	0.005	-0.318	-0.056
age	-0.009	0.009	-0.961	0.336	-0.027	0.009
price	0.402	0.094	4.275	<1.91e-5	0.217	0.586
r	1.14	0.137	8.35	<1e-99	0.873	1.408
θ	≈ 0	0.052	-1.92e-7	1.0	-0.102	0.102
**GLMM**						
(Intercept)	2.580	0.117	21.92	<1e-99	2.350	2.811
sex	-0.187	0.027	-7.04	<1e-11	-0.239	-0.135
age	-0.009	0.001	-7.99	<1e-14	-0.011	-0.007
price	0.402	0.053	7.56	<1e-13	0.298	0.506
r	1.395	NA	NA	NA	NA	NA
θ	≈ 0	NA	NA	NA	NA	NA
**GEE**						
(Intercept)	2.374	0.110	21.64	<1e-99	2.16	2.59
sex	-0.186	0.032	-5.89	<1e-8	-0.248	-0.124
age	-0.009	0.001	-7.01	<1e-11	-0.011	-0.006
price	0.507	0.045	11.14	<1e-28	0.417	0.596
r	NA	NA	NA	NA	NA	NA
θ	NA	NA	NA	NA	NA	NA

Table 2: Comparisons of parameter estimates on the NHANES data under the negative binomial approximate-copula (AC) model, GLMM, and GEE. All $n = 1537$ sampling units are of size $d_i = 2$.

	Loglikelihood	Time (seconds)
AC Poisson	-20690.8	0.181
GLMM Poisson	**-15499.5**	0.529
GEE Poisson	NA	**0.021**
AC NB	**-12037.6**	0.034
GLMM NB	-12047.5	0.867
GEE NB	NA	**0.024**
AC Bernoulli	**-1938.7**	0.06
GLMM Bernoulli	-1980.9	0.821
GEE Bernoulli	NA	**0.016**

Table 1: The loglikelihood and run times for the longitudinal NHANES data under the approximate-copula (AC) model, GLMM, and GEE, using three different base distributions. The best value is bolded. Note the loglikelihoods for GEE are NA since it is not likelihood-based. All $n = 1537$ sampling units are of size $d_i = 2$.

Indeed, the variances for a few of these parameters (sex and age) are larger in our model than in GLMM and GEE. We have added these findings and explanations in Section 3.4 of the main text. In

addition, we provide another table in the supplement to explore changes in parameter values under the Poisson base distribution. The table below is almost the same as Table 2 above, with the only difference being the switch to the Poisson base from the negative binomial base. As noted in the revised text, using the Poisson base in the presence of overdispersion amounts to model misspecification.

	Estimate	Std. Error	z	Pr(> $\|z\|$)	Lower 95%	Upper 95%
**AC**						
(Intercept)	2.509	0.205	12.24	<1e-99	2.107	2.910
sex	-0.210	0.050	-4.232	<2.32e-5	-0.307	-0.113
age	-0.009	0.046	-0.193	0.845	-0.10	0.966
price	0.434	0.271	1.60	0.110	-0.010	0.966
θ	7.080	0.787	8.993	<1e-99	5.537	8.623
**GLMM**						
(Intercept)	2.040	0.084	24.22	<1e-99	1.727	2.351
sex	-0.225	0.036	-6.24	<1e-9	-0.359	-0.091
age	-0.009	0.002	-6.19	<1e-9	-0.015	-0.004
price	0.596	0.023	26.42	<1e-99	0.513	0.681
θ	0.458	NA	NA	NA	NA	NA
**GEE**						
(Intercept)	2.361	0.108	21.85	<1e-99	2.149	2.573
sex	-0.194	0.030	-6.39	<1e-9	-0.253	-0.134
age	-0.007	0.001	-6.3	<1e-9	-0.010	-0.005
price	0.486	0.044	11.14	<1e-28	0.40	0.571
θ	NA	NA	NA	NA	NA	NA

Table 3: Comparisons of parameter estimates on the NHANES data under Poisson approximate-copula (AC) model, GLMM, and GEE. All $n = 1537$ sampling units are of size $d_i = 2$. Here θ is the variance component.

Discussions of these results have also been incorporated into the main text. We trust that these expanded results clarify the strengths, weaknesses, and differences among GEE, GLMM, and our approximate copula model.

4. In Section 3.5, the authors only used the approximate copula distribution to analyze the data. Similar to previous comments, comparisons with existing methods are needed. In addition, the analysis is less appealing given that the authors purposely dichotomized two continuous traits. Are there more natural choices of continuous and categorical traits for the proposed distribution in the real application?

We thank the reviewer for the suggestion. In response, please note that dichotomizing blood pressure is a common practice in the clinical studies where patients are classified as "hypertensive" or "normotensive." Dichotomization of blood pressure in our example is done to illustrate the flexibility of

our model in analyzing mixed continuous and binary traits. In standard GLMM and GEE modeling, this cannot actually be easily accomplished. Dichotomization helps us make our point.

In an attempt to follow up on the reviewer's suggestion about expanding method comparisons to real datasets, we ran into the following roadblocks in the GWAS example.

(1) GWAS comparison with GLMM: In our 3-trait analysis, we construct a multivariate model where each of the 3 traits can have a different base distribution, link function and response to covariates (sex, age, SNP value, etc). In GLMM modeling, each trait must have the same distribution in a longitudinal context and multivariate mixed types are not supported. With this limitation in mind, we can constructed a 3-trait GLMM model with Bernoulli base and logit link function for comparison to our multivariate mixed outcome example. The BMI trait is dichotomized into a binary trait indicating whether the patient is obese (BMI is ≥ 30) or not. With this setup it takes **more than 150 minutes** to fit the GWAS model for a single SNP using the `MixedModels.jl` package under its default settings. Thus, even if we split the data into 400 parts, submit a separate cluster job for each part, and have them all run simultaneously, this analysis would still require **more than 100 days** to complete. This illustrates the computational scalability and flexibility of the approximate copula model. Indeed, we can analyze a mixture of continuous/binary traits with ease and accomplish our fully-likelihood based analysis in a single day.

(2) GWAS comparison with GEE: Our GWAS example uses likelihood ratio tests rather than Wald or score tests in testing SNPs. It is common knowledge that likelihood ratio tests deliver more accurate p-values. We omit a GWAS comparison using GEE modeling because GEEs do not have a well-defined likelihood and cannot by definition produce a likelihood ratio test. One may derive a score test based on the sandwich formula. The only known software that is scalable to biobank data is TrajGWAS (https://doi.org/10.1016/j.ajhg.2022.01.018); however it cannot handle binary trait.

Reviewer 2

This paper introduces a new family of multivariate distributions for the joint modeling of non-Gaussian data, such as those arising in longitudinal data and multi-trait GWAS. The family of distributions is derived from a Gaussian approximation of Tonda (2005) to the likelihood in GLMMs. The paper gives explicit expressions for the moments and marginal and conditional distributions, and develops maximum likelihood procedures for the family, extending the theory and inference procedures of Tonda (2005). While this family of distributions can be a useful alternative to GLMMs, I have major concerns about its connection with existing methods, motivations, and usefulness.

1. The idea is in fact similar to the Laplace approximation for inference in GLMMs. The difference is about which point the Taylor expansion is obtained. The Laplace approximation expands around the conditional mode of the random effect, while the current method expands around the mean of the random effect; see the derivations in Tonda (2005, page 335). It is not immediately clear to me which approximation strategy would be more accurate, although the latter is slightly easier to implement. It would be helpful to see a detailed and thoughtful discussion on the connection and comparison of the two methods.

We thank the reviewer for these remarks. Although the proposed model is inspired by the work of Tonda, who initially was trying to approximate the GLMM density, our goal is not the same. We present an entirely new model with its own properly defined probability density function. The approximate copula extension applies to any base distribution with finite second moments. Our purpose is to transition from independence to dependence.

Accuracy can be interpreted in two senses. A likelihood of an existing model can be approximated by a simpler function. This is what you likely mean by the phrase "expanding around the mean or mode." Mean and mode expansions coincide when a likelihood exhibits sufficient symmetry. Our approximate copula model sometimes exhibits such symmetry, for instance with a Gaussian base. Accuracy can also be interpreted to mean how well a model fits actual data. In this sense, no model is uniformly superior to another. One can assess model fit by comparing maximum loglikelihoods. If the number of parameters in two models are the same, then this comparison is equivalent to an AIC or BIC comparison. In Section 3.4 of our paper, our loglikelihood is slightly higher than that of GLMM on the NHANES longitudinal data under a Negative Binomial or Bernoulli base. Thus, our model can be considered a more accurate fit to the observed data. GLMM may result in a better fit in a different dataset where the underlying true distribution conforms more closely to GLMM assumptions.

2. The NHANES data for longitudinal analysis and the UK Biobank data for multi-trait GWAS have dimensions d = 2 and d = 3, respectively. With such a small d, a GLMM can be easily fit. What is the motivation for adopting the new modeling approach?

We thank the reviewer for raising this point. Fitting time depends on d, p (number of covariates), and n (sample size). In our real data examples, d is small for both examples. If n and p are small, such as in the NHANES longitudinal data, runtimes are modest for all models. However in scenarios where n and p are large, as shown in our response to question 4 of reviewer 1, applying GLMM to biobank-scale data results in an overall runtime exceeding 100 days. This example highlights the computational advantages of our model and its implementation, even when $d = 3$.

Furthermore, the advantages of the approximate copula model extend beyond computational efficiency. It also is flexible in handling mixed data types and fits some datasets better. In the GWAS example, standard GLMM software does not extend to multivariate mixed types from different base distributions. Our model does. The NHANES longitudinal example shows that our model results in a better loglikelihood than GLMM.

3. Page 3, "we find that the model reflects reality best when the number of components of each independent sampling unit is low or the correlations between responses within a unit are small": This seems contrastive to the motivation discussed in Section 1.1, in particular, scalability in high dimensions.

We thank the reviewer for the comment. Scalability is meant in terms of computational efficiency. Our model is scalable because it dispenses with matrix determinants and inverses. The goal of this paper is to explore both the strengths and weaknesses of the model. We frankly acknowledge that the model fits best when sampling units are small. Fortunately, many biological traits exhibit weak dependencies. Regardless of sampling unit size, parameter estimation remains efficient. The limits of the model in accommodating strong dependencies has little to its computational scalability.

4. The simulation study fits GLMMs using MixedModels.jl, mentioning only that "[w]hen the random effect term is a scalar, MixedModels.jl uses Gaussian quadrature for parameter estimation." How about the multivariate case? More information about the algorithm implemented by MixedModels.jl should be provided. In particular, it would be more interesting to compare the proposed method with GLMM fitting with Laplace approximation rather than Gaussian quadrature.

We thank the reviewer for suggesting this simulation. In `MixedModels.jl`, it is relatively easy to switch between using Gaussian quadrature or Laplace approximation by changing the `nAGQ` parameter. Setting `nAPQ`=1 runs Laplace approximation, while setting `nAPQ`>1 runs Gaussian quadrature. Based on this, we repeated the longitudinal Simulation I setup, using the Negative Binomial base, as described in Section 3.1 of the manuscript. The side-by-side comparison of Gaussian Quadrature and Laplace approximation MSE is shown in Figure 1 below and demonstrates that the overall pa-

rameter estimates are rather similar. Because of this, we decided to leave the simulation study in the paper as is.

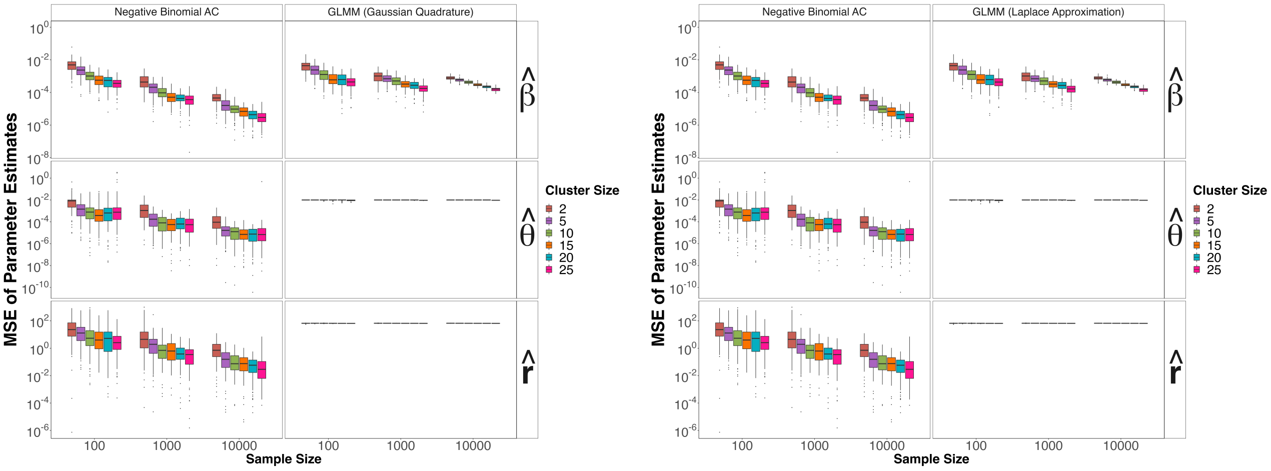

Figure 1: Simulation study under the longitudinal model. Features MSE for $\boldsymbol{\beta}$ and $\boldsymbol{\theta}$ under Simulation I setting with Negative binomial base for GLMM fitted using Gaussian quadrature on the left and Laplace approximation on the right. Here cluster size refers to d_i, the number of observations per sample. AC = Approximate Copula and GLMM = generalized linear mixed model. Visually, the MSE between GLMM using quadrature and GLMM using Laplace Approximation are indistinguishable.

5. Please comment on the convexity and convergence of the proposed algorithm. I doubt if the optimization problem is convex when both the mean and covariance parameters need to be estimated.

We thank the reviewer for this comment. We agree that our objective function is not convex. Indeed, the objective contains both positive and negative logarithms. Although we have no convergence guarantees, the base model estimates with $\boldsymbol{\Gamma} = \mathbf{0}$ gives good initial conditions for estimation of mean parameters. Full maximum likelihood estimation is started from $\boldsymbol{\Gamma} = \mathbf{0}$. In our limited experience, other starting values lead to the same or inferior local modes.

6. The derivations in Sections 2.3–2.5 are lengthy and straightforward, and it is unclear how they are used in the estimation procedures. This part seems to be mainly of theoretical interest and can be greatly shortened.

We thank the reviewer for this comment. Since there is no page limit and we are introducing a new class of distributions, we prefer to highlight both theory and applications. In our opinion, this choice improves the flow of the paper and will appeal to readers keen on understanding the nuts and bolts of the model. More impatient readers can easily skip derivations.

Reviewer 3

The authors proposed an approximate-copula model to model arbitrary correlation structures across different types of outcomes (continuous, binary, count). The marginal means of the approximate-copula model can be further linked with covariates to assess relationships between the multivariate outcomes and the covariates. I think this is a promising idea with potential applications in various fields, but I have a few major comments that I hope the authors can clarify

1. The Γ matrix appears to be the most important quantity in the proposed approximate-copula model. The authors refer to this matrix as "the target covariance matrix" in line 121, page 5. However, in line 141, page 7, the covariance between Y_k and Y_l is approximately equal to $\sigma_k \sigma_l \gamma_{kl}$. This suggests that Γ is more akin to a correlation matrix rather than a covariance matrix. It would be helpful if the authors could clarify the interpretation and terminology used.

We thank the reviewer for pointing out this potential source of confusion. The entry γ_{ij} is neither the covariance nor correlation between Y_i and Y_j. Expressions for $\text{cov}(Y_i, Y_j)$ and $\text{corr}(Y_i, Y_j)$ under our density are explicitly derived in section 2.3 as functions of γ_{ij}. We have modified the text to refer to $\boldsymbol{\Gamma}$ as "the positive semidefinite matrix" rather than the "target covariance matrix".

2. The moment calculations in Section 2.3 seem to rely on the assumption that the spectral norm of Γ is small. If Γ represents a correlation matrix, we can assume that all its diagonal entries are 1. In that case, the spectral norm of Γ depends heavily on the off-diagonal entries—that is, the correlations between outcomes. In the simplest case, where all outcomes are uncorrelated, the spectral norm is 1. However, in extreme cases with strong correlations, the spectral norm can be quite large. This implies that assuming a small spectral norm may restrict the strength of allowable correlations between outcomes. In other words, when the outcomes are highly correlated, the marginal distribution of Y could differ substantially from that of X.

This comment recalls the considerable effort we put into developing model. Please note that $\boldsymbol{\Gamma}$ provides a mechanism for capturing both dependency and over-dispersion. As mentioned in the response to the previous question, $\boldsymbol{\Gamma}$ is a ~~covariance~~ positive semi-definite matrix. Thus, its diagonal elements can be arbitrarily large or small. Large diagonal entries of $\boldsymbol{\Gamma}$ suggest over-dispersion. Large off-diagonal entries suggest trait dependence. If neither complication is present in the data, then standard univariate GLM (base) models are adequate. However, it is imprudent to simply ignore the possibility of dependence or over-dispersion. In any event the approximation

$$\text{Cov}(Y_k, Y_l) \quad = \quad \left[1 + \frac{1}{2}\text{tr}(\boldsymbol{\Gamma})\right]^{-1} \sigma_k \sigma_l \gamma_{kl} \quad \approx \quad \sigma_k \sigma_l \gamma_{kl} + \mathcal{O}(\|\boldsymbol{\Gamma}\|^2)$$

accurately captures the added covariance when $\boldsymbol{\Gamma}$ enters the picture, whether $\|\boldsymbol{\Gamma}\|$ is large or not.

The formerly confusing paragraph now reads in part

> The virtue of the density (1) is that it overcomes the independence restriction and steers the estimated covariance matrix toward the sample covariance matrix of the residuals. Inclusion of the positive semidefinite matrix $\mathbf{\Gamma}$ is designed to achieve this goal. For example, if X_1 is Gaussian and X_2 is Bernoulli, then the density (1) allows one to induce correlation between the two components (one continuous and the other discrete) of the binary random vector $X = (X_1, X_2)^\top$. Note that $g(\mathbf{x})$ is technically not a copula because it only approximately preserves the marginal distributions $f_i(x_i)$. Later, we will see how $g(\mathbf{x})$ tends to inflate marginal variances and accommodate correlation. This can be a blessing rather than curse if correlation is present or the true marginal distributions are over-dispersed compared to the assumed marginal distributions.

3. Related to #2, if the marginal distribution of Y differs considerably from that of X, then the approximate copula model may be modeling something different from the observed data. This could be particularly concerning in the regression step, especially in Equation (2), line 198, page 10, where the marginal mean of X is linked to the covariates. This seems to present a gap, as the approximate copula model focuses on Y, while Equation (2) centers on X, especially when X and Y have different marginal distributions. This appears to be a potential model-specification issue.

We thank the reviewer for the comment. Indeed, in our framework, the covariates are only linked to the base distribution X, and the dependency between elements of Y are induced by the matrix $\mathbf{\Gamma}$. We note that the approximate copula model does not preserve the marginals of X, particularly when the observed correlation is strong. This is made clear in the derivations of the moments. However, as we showed in several real data examples, this assumption may also be reasonable, especially when the observed correlation is weak. Incorporating $\mathbf{\Gamma}$ in the approximate copula framework allows us to break the independence assumption while avoiding explicit computation of determinants/integrals/inverses under a valid likelihood function.

4. In light of the issue raised in #3, it is unclear whether it is possible to construct valid confidence intervals for the MLEs derived in Section 2.9. Some clarification or justification would be appreciated.

We thank the reviewer for the comment. The approximate copula likelihood is a smooth function of the parameters and appears to satisfy standard regularity conditions (identifiability, finite Fisher information, and so forth). Thus, under standard regularity conditions for maximum likelihood estimation, the MLE is consistent and asymptotically efficient.

For confidence intervals, we rely on the sandwich (robust) variance estimator, which estimates the asymptotic covariance as $\mathbf{A}^{-1}\mathbf{M}\mathbf{A}^{-1}$ where $\mathbf{A}$ is the negative Hessian (observed information) and $\mathbf{M}$ is the empirical variance of the score function. This estimator remains valid under weaker con-

ditions than the standard Fisher information formula and is widely used in semiparametric settings. We have added the above clarification to the main text under section 1.2.

5. (Minor) The multivariate GWAS application is very interesting. An alternative approach would be to conduct univariate GWAS for each outcome separately and then apply combination tests across outcomes (e.g., the Cauchy combination test). I would be curious to see whether the proposed multivariate GWAS approach offers any statistical power gains over these univariate strategies.

We thank the reviewer for suggesting an interesting and feasible comparison. Following the reviewer's suggestion, we conducted 3 univariate GWASes via a standard GWAS pipeline. Specifically, we used the PLINK2 software, with BMI treated as a continuous trait and SBP/DBP both dichotomized in the same way as featured in our paper. Then we combined the p-values using Cauchy's combination tests, with all weights set equal to $1/3$. The resulting Manhattan plot is shown in Figure 3.

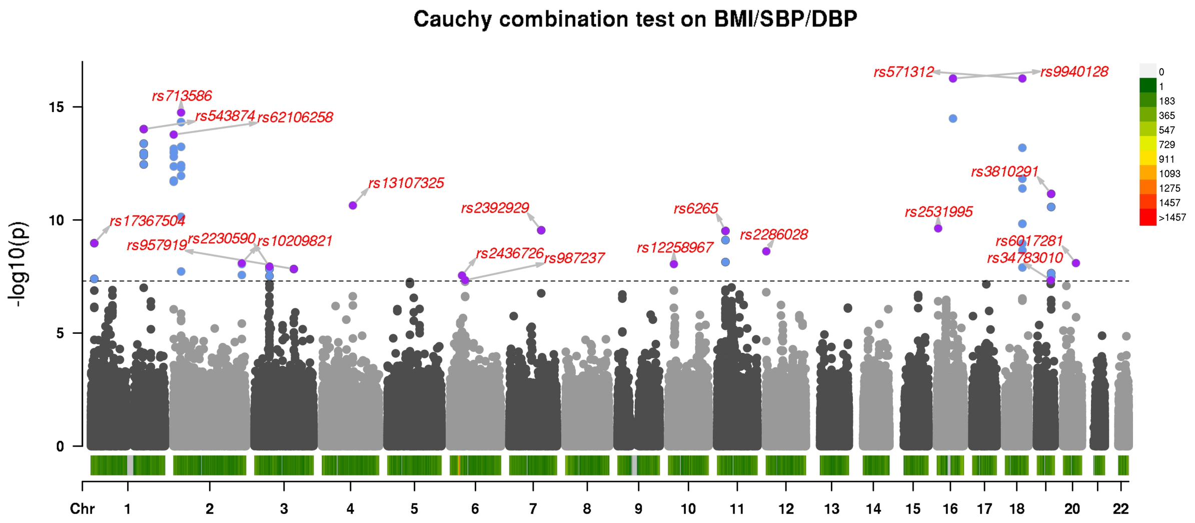

Figure 2: Applying Cauchy combination test to merge 3 univariate GWAS results.

Comparing this result to our approximate copula based GWAS (Figure 3 in main paper), we observe that:

- The total number of independent discoveries is higher in the approximate copula model (24 discoveries) than in the Cauchy combination test (20 discoveries).
- Some discoveries overlap (e.g. rs17367504 and rs173586 in chromosome 1), while a couple of SNPs do not. This is expected, since we label the most significant p-value in a 1Mb window, and the 2 methods could have picked different SNPs in the same genomic neighborhood.

Inspecting the location of the significant regions suggest that the two results agree generally where the significant regions are located.

Although this result is promising, we do not want to over claim results. In particular we do not suggest that the few discoveries missed in the Cauchy combination test but captured in our approximate copula GWAS are biologically meaningful. Furthermore the comparison itself is not perfect, since we are comparing a **standard GWAS pipeline** (standard linear/logistic regression with Wald-test p-values) to that of our approximate copula model (using likelihood ratio tests). Therefore, the results presented here is a simple proof-of-principle work, and as such we chose to include these results only in this round of revision rather than in the current manuscript.

Reviewer 3 comments - Round 2

We thank reviewer 3 for carefully going through our response and for providing helpful comments and suggestions. We have added significant verbiage in our manuscript that clarifies the practical advantages and limitations of the approximate-copula model. Detailed point-to-point responses can be found below.

1. In response to Comment 1, the authors wrote that "the entry γ_{ij} is neither the covariance nor the correlation between Y_i and Y_j." However, in response to Comment 2, they stated that "as mentioned in the response to the previous question, Γ is a covariance matrix." Could the authors please clarify this?

We apologize for the confusion. When we wrote "covariance" matrix in the context of responding to comment 2, we simply meant a matrix that is symmetric and positive semi-definite. In our day-to-day conversation the two has became interchangeable.

For full clarity, the previous reviewer's comment was

> *The moment calculations in Section 2.3 seem to rely on the assumption that the spectral norm of Γ is small. If Γ represents a correlation matrix, we can assume that all its diagonal entries are 1. In that case, the spectral norm of Γ depends heavily on the off-diagonal entries—that is, the correlations between outcomes. In the simplest case, where all outcomes are uncorrelated, the spectral norm is 1. However, in extreme cases with strong correlations, the spectral norm can be quite large. This implies that assuming a small spectral norm may restrict the strength of allowable correlations between outcomes. In other words, when the outcomes are highly correlated, the marginal distribution of Y could differ substantially from that of X.*

And the fully clarified response should read:

This insightful comment recalls the considerable effort we put into developing model. $\mathbf{\Gamma}$ provides a mechanism for capturing both trait correlation and over-dispersion. As mentioned in the response to the previous question, **the only requirement on $\mathbf{\Gamma}$ is that it is symmetric and positive semi-definite**. Thus, its diagonal elements can be arbitrarily large or small. Large diagonal entries of $\mathbf{\Gamma}$ suggest over-dispersion. Large off-diagonal entries suggest trait dependence. If neither complication is present in the data, then standard univariate GLM (base) models are adequate. However, it is imprudent to simply ignore the possibility of dependence or over-dispersion. In any event the equality

$$\mathrm{Cov}(Y_k, Y_l) \quad = \quad \left[1 + \frac{1}{2}\mathrm{tr}(\mathbf{\Gamma})\right]^{-1} \sigma_k \sigma_l \gamma_{kl}$$

accurately captures the added covariance when $\mathbf{\Gamma}$ enters the picture, whether $\|\mathbf{\Gamma}\|$ is large or not.

The formerly confusing paragraph now reads in part

> The virtue of the density (1) is that it overcomes the independence restriction and steers the estimated covariance matrix toward the sample covariance matrix of the residuals. Inclusion of the positive semidefinite matrix $\mathbf{\Gamma}$ is designed to achieve this goal. For example, if X_1 is Gaussian and X_2 is Bernoulli, then the density allows one to induce correlation between the two components (one continuous and the other discrete) of the binary random vector $X = (X_1, X_2)^\top$. Note that $g(\mathbf{x})$ is technically not a copula because it only approximately preserves the marginal distributions $f_i(x_i)$. Later, we will see how $g(\mathbf{x})$ tends to inflate marginal variances and accommodate correlation. This can be a blessing rather than a curse if correlation is present or the true marginal distributions are over-dispersed compared to the assumed marginal distributions.

2. I appreciate the clarification regarding the approximate copula model and its impact on preserving the marginals of X. However, it remains unclear how this approximation might affect the overall performance of the proposed method. The authors mention that the approach tends to perform well when correlations between outcomes are weak (as noted in their response to Comment 3: "this assumption may also be reasonable, especially when the observed correlation is weak"). Could the authors elaborate on what this means in practice? For instance, should practitioners consider using this method primarily when correlations are weak? The current presentation is mathematically detailed, but it would greatly benefit from clearer guidance for applied researchers regarding the strengths and limitations of the method.

This is a fine suggestion. We agree that the paper can benefit from a clearer discussion of the advantages and limitations of the proposed approximate copula model, and giving more practical guidance for applied researchers. Accordingly, we have substantially expanded the **Discussion section** to address these points. In particular, we added two new paragraphs (shown below) that clarify when

the method is most appropriate, its key strengths and weaknesses, and how it complements standard approaches such as GLMM and GEE.

> The approximate-copula model shines when GLMM and GEE are difficult to apply or yield unsatisfactory results. We illustrate this fact for longitudinal binary traits and multiple correlated traits of mixed types. To its credit, the approximate-copula model is likelihood-based, preserves base distributions approximately, and enables hypothesis testing of mean effects and correlations. However, our numerical tests suggest caution. In particular, performance may degrade when correlations between responses are strong or when cluster sizes are large. In this setting the normalizing constant $\ln[1 + \frac{1}{2}\operatorname{tr}(\boldsymbol{\Gamma})]$ grows and tends to shrink estimates of $\boldsymbol{\Gamma}$, making variance component estimates unreliable. In defense of the model, our limited evidence suggests that estimates of mean effects are hardly affected. We suspect that likelihood ratio tests of correlation are also reliable. Given the multivariate nature of the model, it also enhances statistical precision and power in the analysis of mean effects when correlations exist among the traits. In our opinion, variance components are nuisance parameters compared to mean effects in important examples such as GWAS.
>
> In any case, we recommend univariate analysis by standard statistical tools as a prelude to multivariate analysis under the approximate-copula model. Understanding the nature of complicated data is best served when the data is approached from several angles. Despite its simplifications, the approximate-copula model's balance of speed, interpretability, and flexibility make it a valuable complement to existing methods. We hope that other statisticians will agree, work to improve its performance, and apply it to their own data.

3. I appreciate the authors' comparison with univariate GWAS and their careful interpretation of results. However, from a practitioner's perspective, it is still unclear why one should choose this method over the simpler univariate GWAS approach, particularly if it may not provide higher power or stronger biological insights. It would be helpful if the authors could articulate more concretely the advantages of the proposed approach in real applications. PLOS Computational Biology values methodological innovation that provides meaningful biological or practical impact; without such clarification, the application section appears as a technical illustration rather than a demonstration of real-world utility.

We agree with the reviewer's suggestion of adding exposition to better explain the practical advantages of our method versus alternative approaches. Please see the added paragraphs to the discussion section given in the answer to the previous question.

As a concrete example of the model's value, note that our approximate-copula GWAS yielded 4 additional discoveries compared to standard univariate GWAS + Cauchy combination tests. Therefore, our proposed method may well increase power relative to univariate analysis, which in principle translates to stronger biological insights. (These results have now been incorporated in supplemental

Section 1.10 of the manuscript.) To aid the reviewer in understanding the improvement in power, recall that in the last round of revision, we followed the suggestion of running 3 univariate GWASes and subsequently combined the p-values using Cauchy's combination test. We wrote:

> The total number of independent discoveries is higher in the approximate copula model (24 discoveries) than in the Cauchy combination test (20 discoveries).

For the convenience of the reviewer, we provide below 2 Manhattan plots illustrating our findings. The difference in vertical scales is telling.

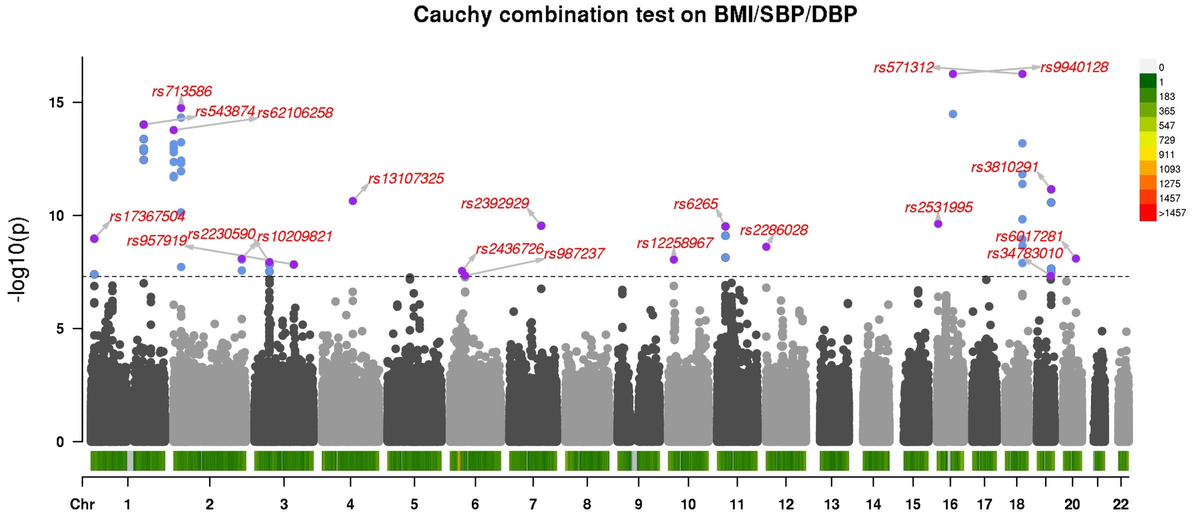

Figure 3: Applying Cauchy combination test to merge 3 univariate GWAS results. There are 20 independent discoveries.

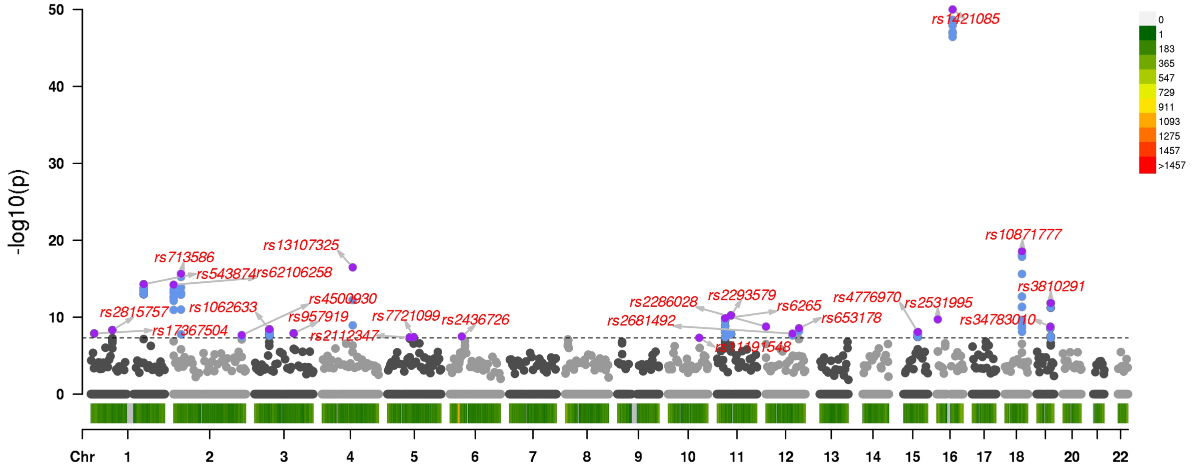

Figure 4: The 3-trait multivariate GWAS under the approximate Copula model. There are 24 independent discoveries.

4. Finally, the authors should carefully review the revised manuscript, as there are still several places where question marks remain in the text.

These mistakes have been corrected in the updated manuscript.
